# The antitumor activity of TGFβ-specific T cells is dependent on IL-6 signaling

**DOI:** 10.1038/s41423-024-01238-7

**Published:** 2024-12-09

**Authors:** Maria Perez-Penco, Mikkel Byrdal, Lucia Lara de la Torre, Marta Ballester, Shawez Khan, Majken Siersbæk, Inés Lecoq, Cecilie Oelvang Madsen, Julie Westerlin Kjeldsen, Inge Marie Svane, Morten Hansen, Marco Donia, Julia Sidenius Johansen, Lars Rønn Olsen, Lars Grøntved, Inna Markovna Chen, Luis Arnes, Morten Orebo Holmström, Mads Hald Andersen

**Affiliations:** 1 National Center for Cancer Immune Therapy (CCIT-DK), Department of Oncology, Copenhagen University Hospital, Herlev, Denmark; 2 Biotech Research and Innovation Centre, Faculty of Health and Medical Sciences, University of Copenhagen, Copenhagen, Denmark; 3 Department of Biochemistry and Molecular Biology, University of Southern Denmark, Odense, Denmark; 4 IO Biotech ApS, Copenhagen, Denmark; 5 Department of Oncology, Copenhagen University Hospital, Herlev, Denmark; 6 Department of Clinical Medicine, Faculty of Health and Medical Sciences, University of Copenhagen, Copenhagen, Denmark; 7 Department of Health Technology, Technical University of Denmark, Lyngby, Denmark; 8 Department of Immunology and Microbiology, University of Copenhagen, Copenhagen, Denmark

**Keywords:** TGFβ, IL-6, immunosuppression, tumor microenvironment, vaccines, Tumour immunology, Immunotherapy, Vaccines, Cytokines

## Abstract

Although interleukin (IL)-6 is considered immunosuppressive and tumor-promoting, emerging evidence suggests that it may support antitumor immunity. While combining immune checkpoint inhibitors (ICIs) and radiotherapy in patients with pancreatic cancer (PC) has yielded promising clinical results, the addition of an anti-IL-6 receptor (IL-6R) antibody has failed to elicit clinical benefits. Notably, a robust TGFβ-specific immune response at baseline in PC patients treated solely with ICIs and radiotherapy correlated with improved survival. Recent preclinical studies demonstrated the efficacy of a TGFβ-based immune modulatory vaccine in controlling PC tumor growth, underscoring the important role of TGFβ-specific immunity in PC. Here, we explored the importance of IL-6 for TGFβ-specific immunity in PC. In a murine model of PC, coadministration of the TGFβ-based immune modulatory vaccine with an anti-IL-6R antibody rendered the vaccine ineffective. IL-6R blockade hampered the development of vaccine-induced T-cells and tumoral T-cell infiltration. Furthermore, it impaired the myeloid population, resulting in increased tumor-associated macrophage infiltration and an enhanced immunosuppressive phenotype. In PC patients, in contrast to those receiving only ICIs and radiotherapy, robust TGFβ-specific T-cell responses at baseline did not correlate with improved survival in patients receiving ICIs, radiotherapy and IL-6R blockade. Peripheral blood immunophenotyping revealed that IL-6R blockade altered the T-cell and monocytic compartments, which was consistent with the findings in the murine model. Our data suggest that the antitumor efficacy of TGFβ-specific T cells in PC depends on the presence of IL-6 within the tumor. Consequently, caution should be exercised when employing IL-6R blockade in patients receiving cancer immunotherapy.

## Introduction

Interleukin-6 (IL-6) is a pleiotropic cytokine that significantly contributes to hematopoiesis and inflammation [1]. The role of IL-6 in immunity is context-dependent, as although it has well-documented proinflammatory properties during acute inflammation, it can also orchestrate crucial anti-inflammatory activities for the resolution of inflammation [1]. In contrast to the protective effects of acute IL-6 production, prolonged IL-6 secretion can contribute to chronic inflammation [1, 2], thereby positioning IL-6 as a central player in cancer promotion and maintenance [3]. Correlations between high levels of circulating IL-6 and poor survival have been observed in various cancers, including pancreatic cancer [4, 5]. Although IL-6 is commonly associated with tumor promotion and immunosuppression, emerging evidence suggests that it can also support antitumor immunity under certain yet not fully elucidated circumstances [2, 3]. For example, IL-6 signaling in macrophages is required for immunotherapy-driven tumor regression in murine tumor models [6]. A recent phase 2 clinical trial, known as “CheckPAC”, reported meaningful antitumor activity when nivolumab and ipilimumab were combined with stereotactic body radiotherapy in patients with metastatic pancreatic cancer [7]. Given the correlation between lower systemic levels of IL-6 and improved clinical outcomes [7], a follow-up phase II trial termed “TRIPLE-R” aimed at assessing the therapeutic benefit of incorporating an IL-6 receptor (IL-6R)-blocking antibody (tocilizumab) into the treatment [8]. Unexpectedly, no clinical responses were observed [8], suggesting that IL-6R blockade might have affected the beneficial effects of combining checkpoint inhibition with radiotherapy in patients with pancreatic cancer. Recently, we reported that high levels of transforming growth factor (TGF)-β-specific T cells at baseline were associated with clinical benefit and improved survival in patients with pancreatic cancer in the CheckPAC study [9]. Furthermore, we demonstrated that a TGFβ-based immune modulatory vaccine (“TGFβ vaccine”) induced a TGFβ-specific T-cell response that controlled tumor growth in a preclinical model of pancreatic cancer [10] through the direct and indirect modulation of tumor-associated macrophages (TAMs) and cancer-associated fibroblasts (CAFs) [10, 11]. Here, we investigated the role of IL-6 in TGFβ-specific immunity in a murine model of pancreatic cancer. The clinical significance of these findings was further explored in patients diagnosed with pancreatic cancer who were included in the CheckPAC or TRIPLE-R clinical trials.

## Results

### *IL6R* is expressed primarily by myeloid cells, whereas *IL6* is expressed predominantly by fibroblasts in pancreatic tumors

**Fig. 1:**
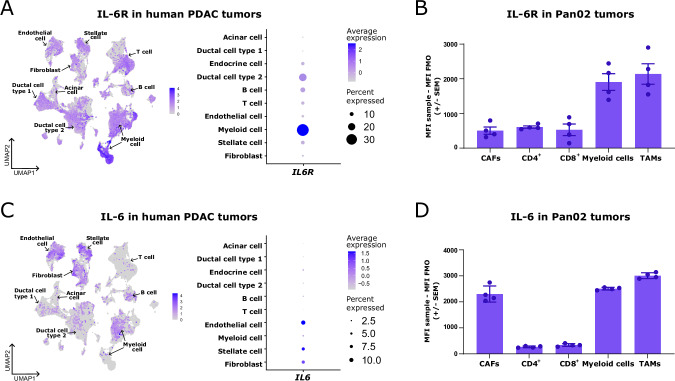
*IL6R* is expressed primarily in myeloid cells, whereas *IL6* is expressed predominantly by fibroblasts in pancreatic tumors. (**A**, left) UMAP plot of 136,163 cells from a publicly available single-cell RNAseq atlas of >70 samples from human pancreatic ductal adenocarcinoma (PDAC) [12] showing 10 different clusters (acinar cell, ductal cell type 1, ductal cell type 2, endocrine cell, B cell, T cell, endothelial cell, myeloid cell, stellate cell, and fibroblast) and the expression of IL6R across the different clusters. (**A**, right) Average normalized expression of IL6R and percentage of IL6R-expressing cells in the different clusters shown in (**A**, left). (**B**) IL-6R mean fluorescence intensity (MFI) in cancer-associated fibroblasts (CAFs), CD4^+^ T cells, CD8^+^ T cells, myeloid cells, and tumor-associated macrophages (TAMs) in Pan02 tumors from untreated mice. (**C**, left) UMAP plot of 136163 cells from a publicly available single-cell RNAseq atlas of >70 samples of human PDAC [12] showing the same 10 different clusters described in (**A**, left) and the expression of IL6 across the different clusters. (**C**, right). Average normalized expression of IL6 and percentage of IL6-expressing cells in the different clusters shown in (**C**, left). (**D**) IL-6 MFI in CAFs, CD4^+^ T cells, CD8^+^ T cells, myeloid cells, and TAMs in Pan02 tumors from untreated mice. For (**B**, **D**), CAFs were gated as live CD45^−^ CD90.2^+^ cells. CD4^+^ T cells were gated as live CD45^+^ CD3^+^ CD4^+^ cells. CD8^+^ T cells were gated as live CD45^+^ CD3^+^ CD8^+^ cells. Myeloid cells were gated as live CD45^+^ CD11b^+^ cells. TAMs were gated as live CD45^+^ CD11b^+^ F4/80^+^ cells. The data are presented as the average ±  SEM. Dots represent individual mice. The IL-6R or IL-6 MFI was calculated by subtracting the MFI in the fluorescence minus one (FMO) control for IL-6R or IL-6 from the MFI in the fully stained sample for each sample

To discern the cellular subsets susceptible to IL-6R blockade that could compromise the responsiveness to immune checkpoint inhibition and radiotherapy, we assessed the expression of *IL6R* in human pancreatic tumors. We used a publicly available single-cell RNA sequencing (scRNAseq) atlas of human pancreatic ductal adenocarcinoma (PDAC) containing over 70 samples [12]. The myeloid cells presented the highest expression of *IL6R* (Fig. 1A). Using flow cytometry, we validated myeloid cells and TAMs as the main IL-6R^+^ cell subsets in Pan02 cells, a murine model of pancreatic cancer [13] (Fig. 1B). We subsequently examined the expression of *IL6* in human PDAC tumors and identified endothelial cells, fibroblasts and pancreatic stellate cells, which are quiescent fibroblasts that can give rise to CAFs [14], as the primary *IL6*-expressing cells (Fig. 1C). We confirmed that CAFs were one of the principal sources of IL-6 in murine Pan02 tumors (Fig. 1D). Intriguingly, myeloid cells and TAMs also emerged as producers of IL-6 (Fig. 1D).

### The TGFβ vaccine increases the IL-6 concentration in tumors and IL-6 expression in cancer-associated fibroblasts

**Fig. 2:**
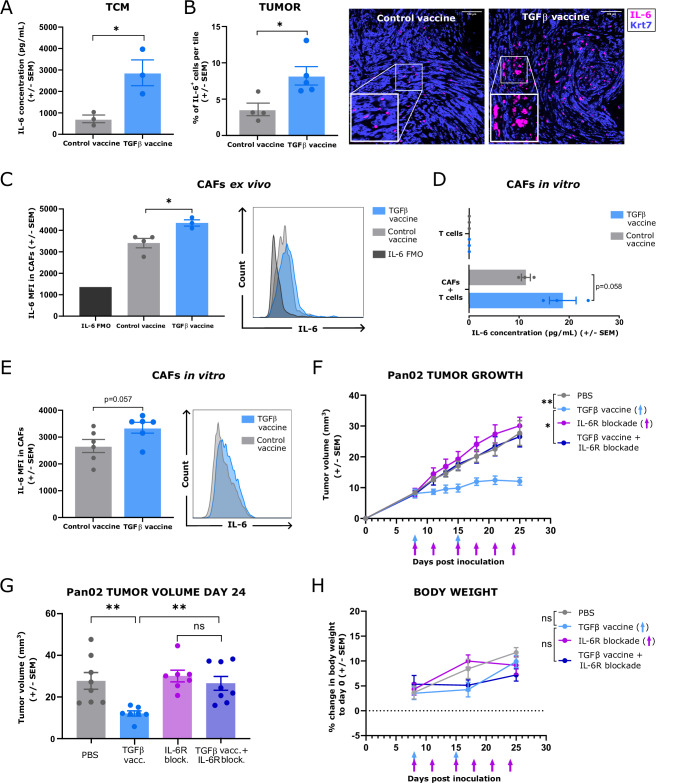
The general increase in IL-6 in the tumor microenvironment, and specifically in cancer-associated fibroblasts, induced by the TGFβ vaccine is necessary for the antitumor activity of the vaccine in the Pan02 murine model of pancreatic cancer. (**A**) Concentration of IL-6 in tumor-conditioned media (TCM) derived from Pan02 tumors from mice that were treated with a control vaccine or with the TGFβ vaccine on days 8 and 15 postinoculation, as quantified by ELISA (*n* = 3 different batches of TCM). (**B**, left) Percentage of IL-6^+^ cells per tile in Pan02 tumors from mice that received either a control vaccine or the TGFβ vaccine (*n* = 4–5 mice per group), as assessed by immunofluorescence staining for IL-6 (pink) and the cancer cell marker keratin-7 (Krt7, blue). Representative immunofluorescence microscopy images are shown in (**B**, right). Scale bar = 100 µm. (**C**, left) Ex vivo mean fluorescence intensity (MFI) of intracellular IL-6 in cancer-associated fibroblasts (CAFs) in the tumors of Pan02 tumor-bearing mice that received either a control vaccine or the TGFβ vaccine, as assessed by flow cytometry (*n* = 3–4 mice per group). The mice were treated with a control vaccine or the TGFβ vaccine on days 8 and 15 postinoculation. The IL-6 MFI was assessed on day 25 post-inoculation. (**C**, right) Representative histograms of intracellular IL-6 expression in the CAFs shown in (**C**, left). (**D**) Concentration of IL-6 in the supernatants of a coculture of CAFs sorted from KPC pancreatic tumors that were cultured at a 1:20 fibroblast: T-cell ratio for 48 h with T cells isolated from the spleens of mice that received two shots, one week apart, of either a control vaccine or the TGFβ vaccine, as assessed by ELISA (*n* = 3 per group). (**E**, left) In vitro MFI of intracellular IL-6 in CAFs from the coculture described in (**D**), as assessed by flow cytometry (*n* = 6 per group). (**E**, right) Representative histograms of intracellular IL-6 expression in the CAFs shown in (**E**, left). For (**C**, **E**), CAFs were gated as live CD45^−^ CD90.2^+^ cells. For (**A**–**E**), the data are presented as the means ± SEMs. Dots represent individual mice. (**F**) Pan02 tumor growth in mice treated with PBS, the TGFβ vaccine, an anti-IL-6R blocking antibody, or the combination of both the TGFβ vaccine and an anti-IL-6R blocking antibody. The TGFβ vaccine was administered subcutaneously (s.c.) in the flank on days 8 and 15 postinoculation. PBS or the anti-IL-6R blocking antibody (200 µg/mouse) was administered s.c. next to the tumor on day 8 postinoculation and every 3^rd^–4^th^ day for a total of six injections. The data are representative of 4 independent experiments and are presented as the means ± SEMs. (**G**) Pan02 tumor volume on day 24 post-inoculation for the different treatment groups shown in (**F**). The data are shown as the means ± SEMs. Dots represent individual mice. (**H**) Changes in body weight over time in Pan02 tumor-bearing mice across the different treatment groups shown in (**F**). The data are presented as the means ± SEMs. For (**F**–**H**), (*n* = 7–8 mice per group). ns, not significant; *p < 0.05 and **p < 0.01 according to unpaired two-tailed *t*-test for (**A**–**E**, **G**) and according to TumGrowth software [42] for (**F**, **H**)

When the cytokine composition of the supernatant of a 48 h culture of digested Pan02 tumors, referred to as “tumor-conditioned media” (TCM), was assessed, we previously inferred that IL-6 levels were higher in TCM from mice treated with the TGFβ vaccine than in TCM from control-vaccinated mice [11]. Here, we confirmed these findings across different batches of TCM (Fig. 2A). The increase in IL-6 levels within the tumor following TGFβ vaccine treatment was corroborated by immunofluorescence, providing direct visualization of elevated IL-6 in the tumor microenvironment (Fig. 2B). Since fibroblasts are key IL-6 producers in pancreatic cancer (Fig. 1C, D), we investigated whether treatment with the TGFβ vaccine could increase IL-6 production in this population, driving the elevated IL-6 levels in TCM. We detected significantly higher levels of IL-6 in CAFs from mice treated with the TGFβ vaccine than in those from control-vaccinated mice (Fig. 2C). Given the ability of TGFβ-specific T cells to modulate the phenotype of CAFs in vitro [11] and in vivo [10], we assessed whether this finding was a direct effect of TGFβ-specific T cells. CAFs were sorted from subcutaneous KPC pancreatic tumors and cocultured with splenic T cells from vaccinated mice. Compared with those from control mice, CAFs cultured with T cells from mice treated with the TGFβ vaccine tended toward increased IL-6 secretion (Fig. 2D) and higher intracellular IL-6 levels (Fig. 2E). These findings imply that IL-6 levels within the tumor microenvironment (TME) may be increased upon treatment with the TGFβ vaccine, potentially due to the polarization of CAFs by TGFβ-specific T cells toward an increased IL-6-secreting phenotype.

### IL-6 is required for the antitumor activity of the TGFβ vaccine

To assess whether the increase in IL-6 in the TME induced by the TGFβ vaccine has any relevant implications for the immunotherapeutic effect of the vaccine, we impaired the IL-6 axis via the use of an anti-IL-6R blocking antibody. Mice were subcutaneously inoculated with Pan02, a model of PDAC characterized by high *Tgfb1* expression [15], and treated with the TGFβ vaccine as previously described [10]. The anti-IL-6R blocking antibody was administered locally next to the tumor on day 8 postinoculation and every 3^rd^–4^th^ day for a total of six injections, following the schedule described by Nejad et al [6]. Interestingly, we observed that blocking IL-6R completely abolished the antitumor effect of the TGFβ vaccine in four independent experiments (Fig. 2F, G). None of the treatments affected the evolution of body weight with time (Fig. 2H).

### Disruption of IL-6 signaling impairs the development of CD4^+^ vaccine-specific T cells and T-cell infiltration in tumors

**Fig. 3:**
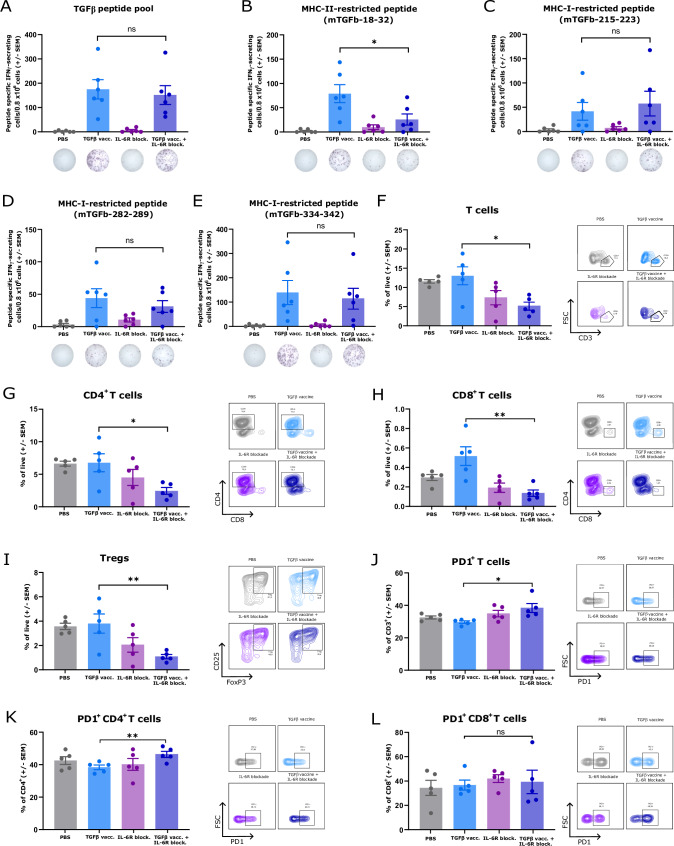
IL-6 signaling blockade hampers the development of CD4^**+**^ vaccine-specific T cells and impairs T-cell infiltration in tumors. (**A**) Specific IFNγ response against the pool of TGFβ-derived peptides that constitute the TGFβ vaccine in the spleen of Pan02 tumor-bearing mice that were treated with PBS, the TGFβ vaccine, IL-6R blockade or the combination of the TGFβ vaccine and IL-6R blockade, as described in Fig. 2, as assayed by IFNγ ELISpot at the endpoint (day 25 postinoculation). (**B**–**E**) Specific IFNγ response against the individual peptides that constitute the TGFβ vaccine: (**B**) mTGFb-18-32, (**C**) mTGFb-215-223, (**D**) mTGFb-282-289 and (**E**) mTGFb-334-342 in the spleens of Pan02 tumor-bearing mice across the different treatment groups, as assayed by IFNγ ELISpot at the endpoint. For (**A**–**E**), a representative example of IFNγ ELISpot responses can be found at the bottom of each panel. *n* = 6 mice per group. (**F**–**L**) Characterization of T-cell subsets in Pan02 tumors from mice that were treated with PBS, the TGFβ vaccine, IL-6R blockade, or the combination of the TGFβ vaccine and IL-6R blockade, as described in Fig. 2, as assayed by flow cytometry at the endpoint (day 25 postinoculation). Bar plots showing (**F**) T cells gated as CD45^+^ CD3^**+**^; (**G**) CD4^+^ T cells gated as CD45^+^ CD3^+^ CD4^+^; (**H**) CD8^+^ T cells gated as CD45^+^ CD3^+^ CD8^+^ and (**I**) regulatory T cells (Tregs) gated as CD45^+^ CD3^+^ CD4^+^ Foxp3^+^ CD25^+^ as a percentage of live cells; (**J**) PD1^+^ T cells; (**K**) PD1^+^ CD4^+^ T cells and (**L**) PD1^+^ CD8^+^ T cells as a percentage of the parent gate. All populations were gated on single live cells. For (**F**–**L**), representative contour plots can be found on the right of each panel. *n* = 5 mice per group. The data in (**A**–**L**) are presented as the means ± SEMs. Dots represent individual mice. ns, not significant; *p < 0.05 and **p < 0.01 according to the unpaired two-tailed *t* test. The data are representative of 3 independent experiments

We explored whether blockade of IL-6 signaling in tumors could affect the development of TGFβ vaccine-induced immunity, which could explain the loss of antitumor efficacy of the vaccine. Using an enzyme-linked immunosorbent spot (ELISpot) assay, we found that IL-6R blockade did not affect the overall TGFβ-specific T-cell response generated by the TGFβ vaccine (Fig. 3A). However, it significantly reduced the number of T cells specific for the major histocompatibility complex (MHC-II)-restricted peptide mTGFb-18-32 (Fig. 3B), with no changes in the immune response to the remaining MHC-I-restricted peptides included in the vaccine (Fig. 3C–E). Next, we used flow cytometry to evaluate the impact of IL-6R blockade on the T-cell compartment within the TME. When comparing mice treated with the TGFβ vaccine to those receiving the vaccine alongside an anti-IL-6R antibody, we found that inhibition of IL-6 signaling reduced overall T-cell infiltration within the tumor (Fig. 3F), including decreases in CD4^+^ T cells (Fig. 3G), CD8^+^ T cells (Fig. 3H) and regulatory T cells (Fig. 3I). Furthermore, a lack of IL-6 signaling resulted in a greater percentage of programmed cell death protein-1 (PD1)^+^ T cells (Fig. 3J), which were specifically found in the CD4^+^ population (Fig. 3K) but not in the CD8^+^ T-cell population (Fig. 3L). These results establish that disrupting IL-6 signaling in Pan02 tumors during active immune modulation by the TGFβ vaccine impairs the development of CD4^+^ TGFβ-specific T cells, hampers tumor T-cell infiltration, and contributes to CD4^+^ T-cell exhaustion.

### Tumor-associated macrophages become more abundant, less proinflammatory, and more immunosuppressive in the absence of IL-6 signaling

**Fig. 4:**
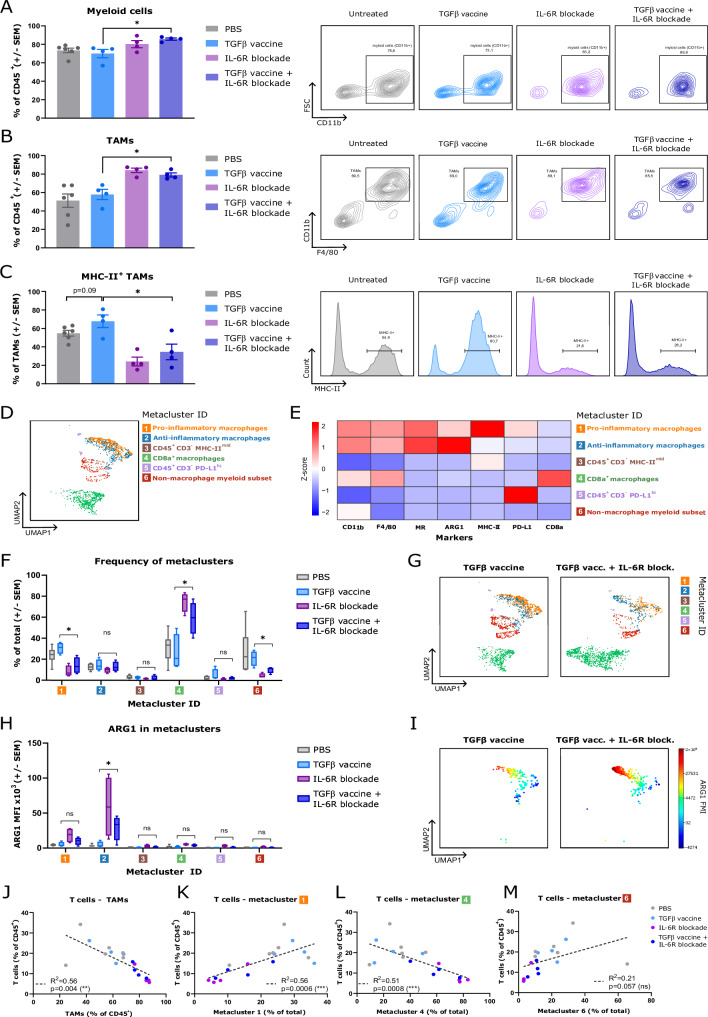
Disruption of IL-6 signaling in the tumor results in a greater abundance of tumor-infiltrating myeloid cells, higher levels of tumor-associated macrophages, an increase in their suppressive phenotype, and a reduction in the proinflammatory TAM subset. (**A**, left) Percentages of myeloid cells (gated as live, CD45^+^ CD3^−^ CD11b^+^ cells) among CD45^+^ cells in the tumors of Pan02 tumor-bearing mice that were treated with PBS, the TGFβ vaccine, IL-6R blockade, or the combination of the TGFβ vaccine and IL-6R blockade, as described in Fig. 2, were assayed via flow cytometry. (**A**, right) Representative contour plots for the data shown in (**A**, left). (**B**, left) Percentage of tumor-associated macrophages (TAMs) among CD45^+^ cells across treatment groups. TAMs were gated as live CD45^+^ CD3^−^ CD11b^+^F4/80^+^ cells. (**B**, right) Representative contour plots for the data shown in (**B**, left). (**C**, left) Percentages of MHC-II^+^ TAMs among total TAMs across treatment groups. (**C**, right) Representative histograms for MHC-II expression for the data shown in (**C**, left). For (**A**–**C**), the data are presented as the means ± SEMs. Dots represent individual mice. Data were collected at the endpoint (day 25 post-inoculation). (**D**) UMAP displaying the meta clusters identified via the FlowSOM unsupervised clustering algorithm in the Cytobank platform on the live CD45^+^ CD3^−^ population of 18 samples (*n* = 4–6 mice per group) identified via flow cytometry and assessed at the endpoint (day 25 postinoculation). (**E**) Heatmap showing the normalized expression by column of CD11b, F4/80, mannose receptor (MR), arginase-1 (ARG1), MHC-II, programmed death-ligand 1 (PD-L1) and CD8a using the Z score across the six different metaclusters identified in the FlowSOM analysis shown in (**D**). (**F**) Frequencies of the six different metaclusters identified in the FlowSOM analysis across treatment groups. The data are presented in a box-and-whisker plot. *n* = 4–6 mice per group. (**G**) Representative UMAPs of a sample derived from a mouse treated with the TGFβ vaccine and a sample derived from a mouse treated with both the TGFβ vaccine and an anti-IL-6R antibody, showing how the metaclusters identified via FlowSOM changed between the treatment groups. (**H**) ARG1 mean fluorescence intensity (MFI) in the six metaclusters identified via FlowSOM across treatment groups. The data are presented in a box-and-whisker plot. *n* = 4–6 mice per group. (**I**) Representative UMAPs of a sample derived from a mouse treated with the TGFβ vaccine and a sample derived from a mouse treated with both the TGFβ vaccine and an anti-IL-6R antibody, displaying the ARG1 MFI for metacluster 2. (**J**) Correlation between the percentage of T cells of total CD45^+^ cells and the percentage of TAMs of total CD45^+^ cells in Pan02 tumors. (**K**–**M**) Correlations between the percentage of T cells of total CD45^+^ cells and the frequencies of (**K**) metacluster 1, (**L**) metacluster 4, and (**M**) metacluster 6 as a percentage of the total. For (**J**–**M**), the dots represent individual mice. *n* = 4–6 mice per group. The treatment groups are color-coded. Correlations were performed with data collected at the endpoint (day 25 postinoculation). ns, not significant; *p < 0.05 and **p < 0.01 according to an unpaired two-tailed *t-*test for (**A**–**C**, **F,** **H**) and linear regression for (**J**–**M**)

Given the elevated expression of IL-6R in myeloid cells in pancreatic tumors (Fig. 1A, B), we hypothesized that myeloid cells, specifically macrophages, could be notably affected by IL-6R blockade. Using flow cytometry, we found that blocking IL-6 signaling increased the abundance of myeloid cells in Pan02 tumors (Fig. 4A), which was accompanied by an increase in the percentage (Fig. 4B) and absolute quantity of TAMs (Supplementary Fig. 1A). In murine PDAC models, approximately two-thirds of TAMs originate from circulating monocytes, while one-third are embryonically derived [16]. We explored whether disrupting IL-6 signaling in the TME could increase monocyte migration from the bone marrow into the tumor. We found that this was not the case, as monocytes isolated from the bone marrow of mice treated with an IL-6R blocking antibody exhibited equivalent migratory capabilities toward a chemoattractant compared with those from the PBS-treated group (Supplementary Fig. 1B).

Notably, blocking IL-6R reduced the abundance of MHC-II^+^ TAMs in mice subjected to the combination treatment compared with those treated with only the TGFβ vaccine (Fig. 4C). To further characterize changes in the intratumoral myeloid population, we used FlowSOM to identify different cell subsets in the CD45^+^ CD3^−^ population via single-cell flow data. The analysis yielded 6 metaclusters (MCs, Fig. 4D, E), which were annotated as described in the methods section. Within metaclusters annotated as macrophages (MC1, MC2, and MC4), we identified a proinflammatory (MC1), an anti-inflammatory (MC2), and a CD8a^+^ (MC4) cluster (Fig. 4D, E). Hampering IL-6 signaling reduced the percentage of proinflammatory macrophages (MC1) and nonmacrophage myeloid subset (MC6) while increasing the frequency of CD8a^+^ macrophages (MC4) in mice treated with the TGFβ vaccine and anti-IL-6R blocking antibody compared with those treated with the vaccine alone (Fig. 4F, G). Although no significant changes in the percentage of anti-inflammatory macrophages (MC2) were detected (Fig. 4F, G), IL-6R blockade increased the expression of arginase-1 (ARG1) in MC2 cells (Fig. 4H, I). Next, we evaluated whether these alterations were IL-6-specific or indirect outcomes of the lack of IL-6 signaling in the tumor. To do so, macrophages were differentiated from bone marrow progenitors in the presence or absence of IL-6, mimicking the condition in the tumor following IL-6R blockade, and the mean fluorescence intensity (MFI) of macrophage-related markers was assessed. In the absence of IL-6, bone marrow-derived macrophages (BMDMs) were smaller (Supplementary Fig. 1C) and, in contrast to our in vivo observations, had a lower mannose receptor MFI (Supplementary Fig. 1D). However, differentiating BMDMs in the absence of IL-6 resulted in mature macrophages with a reduced MHC-II MFI (Supplementary Fig. 1E) and an increased ARG1 MFI (Supplementary Fig. 1F), as observed in vivo. No changes in *H2-D1/K1* (MHC-I) expression were detected (Supplementary Fig. 1G). These data suggest that the changes in TAMs observed after IL-6R blockade are IL-6-specific. We identified a negative correlation between the frequency of T cells and TAMs in tumors across all treatment groups (Fig. 4J). When the correlation with metaclusters that were significantly altered by IL-6R blockade was examined, we found that MC4 (CD8a^+^ macrophages) was negatively correlated with T-cell frequency, whereas MC1 (proinflammatory macrophages) and MC6 (nonmacrophage myeloid subset) were positively correlated with T-cell abundance in tumors (Fig. 4K–M). These findings suggest that the absence of IL-6 signaling in the TME might impair the antitumor efficacy of the TGFβ vaccine by increasing the infiltration of myeloid cells and TAMs into the tumor, reducing the frequency of proinflammatory TAMs and increasing the suppressive phenotype of anti-inflammatory TAMs.

### IL-6R blockade promotes changes in the gene expression profile of tumors related to myeloid migration, myeloid immunity, and T-cell inhibition

**Fig. 5:**
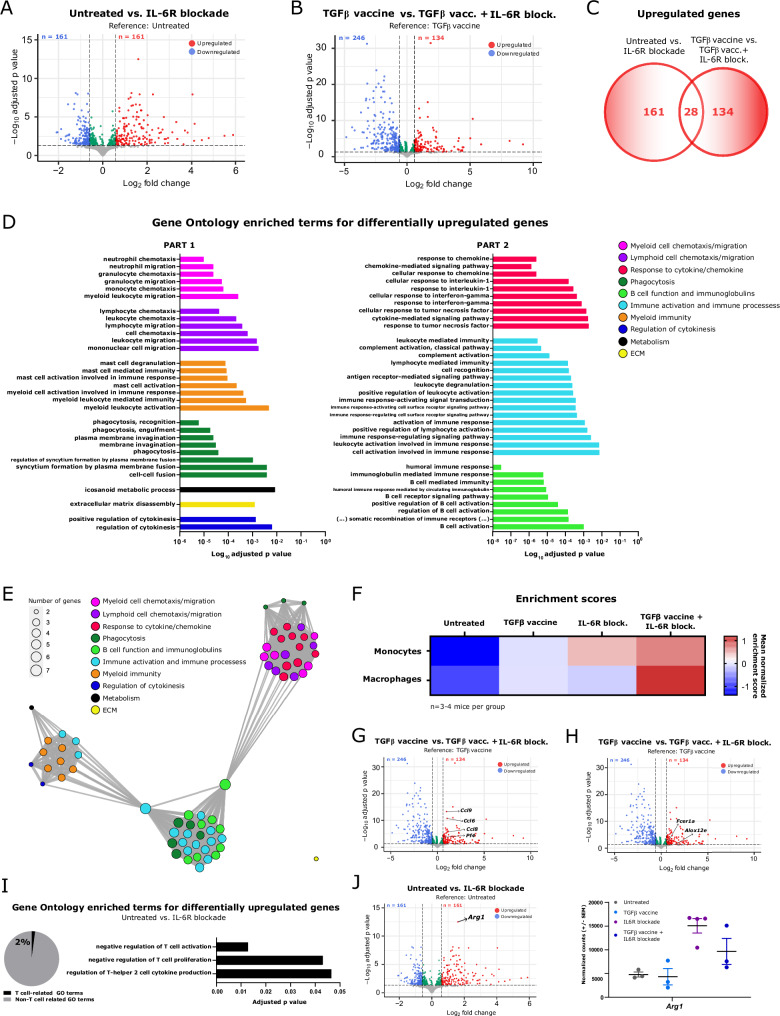
Changes in intratumoral gene expression induced by IL-6R blockade are associated mainly with myeloid migration and immunity, as well as with T-cell inhibition. (**A**) Volcano plot showing differentially expressed genes in Pan02 tumors from mice treated with an anti-IL-6R blocking antibody compared with those in tumors from untreated mice (*n* = 3–4 per group). *n* = 161 upregulated genes and *n* = 161 downregulated genes. (**B**) Volcano plot showing differentially expressed genes in Pan02 tumors from mice treated with the TGFβ vaccine in combination with an anti-IL-6R blocking antibody compared with those in tumors from mice treated with the TGFβ vaccine as monotherapy (*n* = 3 per group). *n* = 134 upregulated genes and *n* = 246 downregulated genes. For (**A**, **B**) False discovery rate (FDR) < 0.05 and absolute log2-fold-change > 0.585. (**C**) Venn diagram showing the overlap in the lists of differentially upregulated genes described in (**A**, **B**). A total of 28 genes were identified as differentially upregulated genes in both comparisons. (**D**) Gene Ontology (GO) enrichment analysis for biological processes associated with the 28 significantly upregulated genes described in (**C**). The 66 GO terms related to cancer immunity, of a total of 93 identified GO terms, are shown. The GO terms are classified into 10 different categories. (**E**) Enrichment map of the 66 GO terms related to cancer immunity showing five functional modules: 1) myeloid and lymphoid cell chemotaxis and phagocytosis, 2) myeloid immunity, 3) immune activation and B-cell function, 4) the extracellular matrix, and 5) metabolism signaling. (**F**) Mean normalized enrichment scores for monocytes and macrophages across treatment groups inferred via cell type deconvolution analysis (*n* = 3-4 mice per group). (**G**) Volcano plot showing differentially expressed genes in Pan02 tumors from mice treated with the TGFβ vaccine in combination with an anti-IL-6R blocking antibody compared with those in tumors from mice treated with the TGFβ vaccine as a monotherapy, where *Ccl6*, *Ccl8,* *Ccl9,* and *Pf4* are highlighted. (**H**) Volcano plot showing differentially expressed genes in Pan02 tumors from mice treated with the TGFβ vaccine in combination with an anti-IL-6R blocking antibody compared with those in tumors from mice treated with the TGFβ vaccine as a monotherapy, where *Alox12e* and *Fcer1a* are highlighted. **I** (left) Pie chart displaying the percentage of T-cell-related GO terms (*n* = 3) of total cancer immunity-related GO terms (*n* = 144) identified in the GO enrichment analysis for biological processes associated with the 161 upregulated genes identified in the differential gene expression analysis comparing tumors from mice that received IL-6R blockade to those from untreated mice. GO terms unrelated to cancer immunity (*n* = 49) were excluded from the data visualization. (**I**, right) T-cell-related GO terms identified in the GO enrichment analysis described in (**I**, left). (**J**, left) Volcano plot showing differentially expressed genes in Pan02 tumors from mice treated with an anti-IL-6R blocking antibody compared with those in tumors from untreated mice, where *Arg1* is highlighted. (**J**, right) Expression levels were assessed by RNA-seq and are presented as VST-normalized counts of *Arg1* in Pan02 tumors across treatment groups. The data are presented as the means ± SEMs. The dots represent individual mice (*n* = 3–4 per group)

To gain deeper insights into the mechanism by which disruption of IL-6 signaling abolishes the antitumor activity of the TGFβ vaccine, Pan02 tumors were harvested, and bulk RNAseq was performed. Differential gene expression analysis revealed 322 differentially expressed genes (DEGs) in mice that received the IL-6R blockade compared with those in untreated mice (Fig. 5A and Supplementary Table 1) and 383 DEGs in mice that received the TGFβ vaccine compared with those in mice that received the TGFβ vaccine together with the IL-6R blockade (Fig. 5B and Supplementary Table 2). We selected genes whose expression was upregulated in both comparisons (*n* = 28) (Fig. 5C and Supplementary Table 3) and performed Gene Ontology (GO) enrichment analysis for biological processes. The analysis returned 93 GO terms, which were classified into different categories (Supplementary Table 4). We identified 66 GO terms within categories related to cancer immunity (Fig. 5D, E). IL-6R blockade upregulated genes linked to myeloid cell chemotaxis/migration and myeloid immunity (Fig. 5D, E). To determine the predominant myeloid populations that infiltrated the tumor in the absence of IL-6 signaling, we performed deconvolution analysis to calculate the relative enrichment of different myeloid subsets. This analysis revealed an increased frequency of tumor-infiltrating monocytes and macrophages following IL-6R blockade (Fig. 5F), which was consistent with the flow cytometry data. We examined specific genes linked to the GO terms enriched under the “myeloid cell chemotaxis/migration” category and found that *Pf4* (*Cxcl4*), *Ccl6*, *Ccl8*, and *Ccl9* were significantly upregulated upon IL-6R blockade (Fig. 5G). This finding was confirmed by qRT-PCR (Supplementary Fig. 1H). These findings suggest that the increased tumoral expression of these chemokines in the absence of IL-6 signaling might act as chemoattractants for myeloid cells. As TAMs are among the main *Il6r*-expressing cells in the Pan02 TME, we hypothesized that IL-6 signaling in TAMs might downregulate the expression of previously identified cytokines/chemokines, thereby preventing additional myeloid cell recruitment. To assess this, macrophages were differentiated from bone marrow progenitors in the presence or absence of IL-6, with the aim of mirroring the conditions that monocytes that infiltrate the tumor and differentiate into macrophages would encounter upon IL-6R blockade. We found that the lack of IL-6 upregulated the expression of *Ccl9* (Supplementary Fig. 1I), a chemokine with well-described monocyte/macrophage chemoattractant properties [17–19]. These data suggest that the absence of IL-6 signaling in TAMs increases *Ccl9* expression, which may induce monocyte chemotaxis and migration into tumors.

Recently, the presence of intratumoral lipid-loaded macrophages has been reported, establishing an association with immunosuppression and cancer progression [20, 21]. Notably, the upregulation of genes involved in metabolism was also observed following the impairment of IL-6 signaling (Fig. 5D, E). In association with the GO term “icosanoid metabolic process” in the “metabolism” category, we identified *Alox12e* and *Fcer1a* as upregulated genes (Fig. 5H), a finding that was confirmed by qRT‒PCR (Supplementary Fig. 1H). Given the pronounced impact of IL-6R blockade on macrophages, these findings might indicate that the lack of IL-6 signaling in the TME could influence lipid metabolism in macrophages.

Among the 28 common upregulated genes, no genes were associated with T-cell function-related GO processes (Fig. 5D). However, when performing GO enrichment analysis for biological processes with the 161 upregulated genes in tumors from mice that received IL-6R blockade compared with those from untreated mice, we found 193 GO terms, 49 of which were not related to cancer immunotherapy (Supplementary Table 5). Among the remaining terms, three were associated with T-cell biology, predominantly reflecting processes related to the inhibition of T-cell activation and proliferation (Fig. 5I). Furthermore, differential gene expression analysis revealed significant upregulation of *Arg1* expression in tumors upon impaired IL-6 signaling (Fig. 5J), which was confirmed by qRT‒PCR (Supplementary Fig. 1H). This finding is in line with the increased levels of ARG1 in anti-inflammatory macrophages identified through FlowSOM analysis. Taken together, our preclinical studies demonstrate that the lack of IL-6 signaling in tumors strongly impairs the antitumor activity of the TGFβ vaccine, as illustrated in Supplementary Fig. 2.

### The incorporation of IL-6R blockade into the treatment regimen renders patients with pancreatic cancer with high baseline TGFβ-specific immunity unresponsive to immune checkpoint inhibitors and radiotherapy

The CheckPAC trial demonstrated a clinical benefit in patients with pancreatic cancer treated with nivolumab, ipilimumab, and radiotherapy compared with those receiving only nivolumab and radiotherapy [7]. We observed that enhanced survival was correlated with a strong TGFβ-specific T-cell response in peripheral blood mononuclear cells (PBMCs) at baseline, defined as more than 50 specific T cells against the human TGFβ-derived peptide TGFβ-15 [22] per 2.5×10^5^ cells [9]. Surprisingly, the addition of an IL-6R blocking antibody (tocilizumab) in combination with nivolumab, ipilimumab and radiotherapy failed to induce clinical responses in patients in the TRIPLE-R clinical trial [8]. Given the detrimental effects of IL-6R blockade on TGFβ-specific immunity in pancreatic cancer observed in our preclinical studies and the sole distinction between patients in the CheckPAC and TRIPLE-R trials being tocilizumab treatment, we compared patients across the two studies to elucidate any impact of IL-6R blockade.

**Fig. 6:**
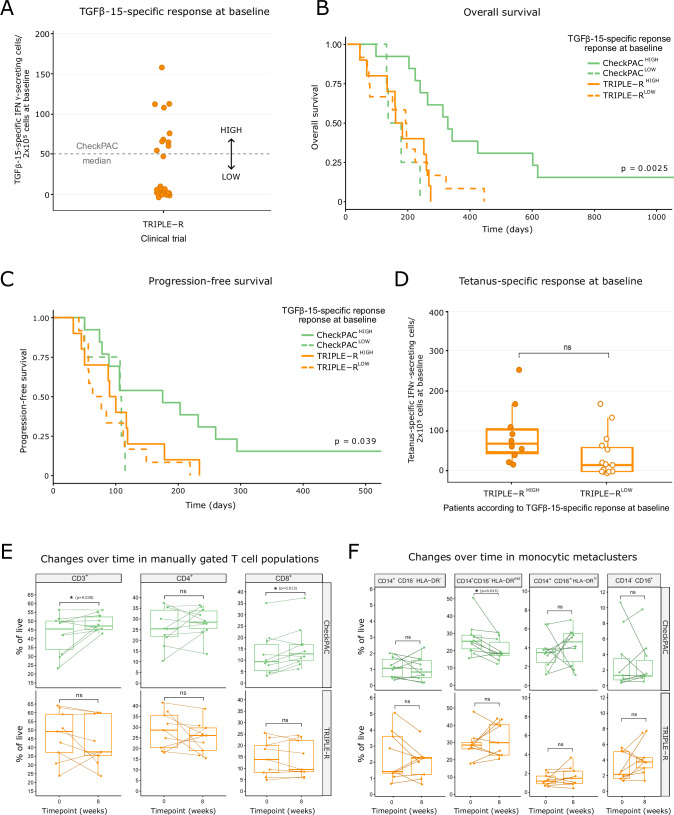
Patients with pancreatic cancer with high TGFβ-specific immunity at baseline do not benefit from immune checkpoint inhibitors and radiotherapy if IL-6R blockade is added to the treatment regimen. (**A**) Number of specific T-cell responses to the peptide TGFβ-15 per 2.5×10^5^ peripheral blood mononuclear cells (PBMCs) from patients in the TRIPLE-R study (*n* = 25 patients), as assayed via IFNγ ELISpot. The median IFNγ response for the CheckPAC study (50 spots) is shown. Dots represent individual patients. (**B**) Kaplan‒Meier curve of overall survival (OS) for patients in the CheckPAC study treated with radiotherapy and both immune checkpoint inhibitors (ipilimumab and nivolumab) with high (*n* = 13 patients) and low (*n* = 4 patients) TGFβ-15-specific T-cell responses and for patients in the TRIPLE-R study with high (*n* = 10 patients) and low (*n* = 12 patients) TGFβ-15-specific T-cell responses. Survival analysis for all patients in the CheckPAC trial on the basis of TGFβ-15-specific immunity at baseline was previously published [9]. Here, we assessed survival on the basis of TGFβ-15-specific immunity at baseline only for patients in the CheckPAC trial who received radiotherapy, nivolumab, and ipilimumab to enable comparison with those in the TRIPLE-R trial. (**C**) Kaplan‒Meier curve of progression-free survival (PFS) for the patients described in (**B**). For (**B**, **C**), statistical significance was calculated via a log-rank test. (**D**) Number of specific T cells against a tetanus-derived peptide per 2.5 × 10^5^ PBMCs from patients in the TRIPLE-R study with high (*n* = 10 patients) and low (*n* = 15 patients) TGFβ-15-specific T-cell responses. The data are presented in a box-and-whisker plot, with dots representing individual patients. *p < 0.05 according to an unpaired two-tailed *t*-test. (**E**) Percentages of CD3^+^ cells, CD4^+^ T cells and CD8^+^ T cells of live cells in PBMCs at baseline and 8 weeks post-treatment initiation for patients who were treated with both immune checkpoint inhibitors (ipilimumab and nivolumab) and who had a high TGFβ-15-specific T-cell response at baseline in the CheckPAC trial (*n* = 10 patients) and for patients with a high TGFβ-15-specific T-cell response at baseline in the TRIPLE-R trial (*n* = 9 patients). (**F**) Percentages of the following monocytic metaclusters: CD14^+^ CD16^−^ HLA-DR^−^ (HLA-DR^−^ classical monocytes), CD14^+^ CD16^−^ HLA-DR^mid^ (HLA-DR^mid^ classical monocytes), CD14^+^ CD16^+^ HLA-DR^hi^ (monocytes in an intermediate state) and CD14^−^ CD16^+^ (nonclassical monocytes) of total live cells in PBMCs at baseline and 8 weeks post-treatment initiation for the patients described in (**E**). For (**E**, **F**), the data are presented in a box-and-whisker plot, with dots representing individual patients and *p < 0.05 according to a paired two-tailed *t*-test. Characterization of human PBMCs via flow cytometry at baseline and 8 weeks posttreatment (w8) was performed on all patients from whom PBMCs were available at both timepoints (*n* = 10 patients for the CheckPAC trial and *n* = 9 for the TRIPLE-R trial)

First, we confirmed that a TGFβ-15-specific T-cell response at baseline in PBMCs could also be detected in patients in the TRIPLE-R trial and that these patients could be stratified into high and low responders according to the same cutoff that was used for the CheckPAC trial [9] (Fig. 6A). Next, we compared the survival rates among participants in the CheckPAC and TRIPLE-R trials on the basis of their TGFβ-15-specific immunity at baseline, as previously reported by Mortensen et al. in all patients in the CheckPAC study [9]. Here, we exclusively selected patients in the CheckPAC trial who were treated with radiotherapy, nivolumab, and ipilimumab to enable comparison with those in the TRIPLE-R trial. We confirmed that high TGFβ-15-specific immunity was correlated with increased survival in patients in the CheckPAC trial (Fig. 6B, C). Conversely, a high TGFβ-15-specific T-cell response at baseline did not provide additional survival benefits for patients in the TRIPLE-R trial (Fig. 6B, C). To ensure that differences in TGFβ-15-specific immunity in the TRIPLE-R trial were not explained by a general state of immune suppression, we quantified the immune response to a *Clostridium tetani-*derived peptide (referred to as “tetanus”) at baseline in PBMCs from patients in the TRIPLE-R trial, as previously performed for the CheckPAC trial [9]. We observed no significant differences in the tetanus-specific immune response (Fig. 6D), which suggests that patients with weak TGFβ-15-specific T-cell responses were not generally immunocompromised. To control for any potential variables that could confound the differences in survival among patients with high TGFβ-15-specific T immunity at baseline in the CheckPAC and TRIPLE-R trials, we compared clinical parameters between the two patient cohorts and found no statistically significant differences (Supplementary Table 6).

Next, we assessed whether treatment with IL-6R blockade could alter the immune system, potentially compromising the advantageous antitumor effects of TGFβ-15-specific T cells. We used flow cytometry to phenotype immune subsets in PBMCs from patients in the TRIPLE-R trial and from patients treated with both immune checkpoint inhibitors in the CheckPAC trial. From these two cohorts, patients with a high TGFβ-15-specific immunity at baseline were selected. Phenotyping was performed at baseline and after 4 cycles of treatment (week 8). When examining the T-cell compartment, we verified that patients in both studies had similar T-cell frequencies at baseline (Supplementary Fig. 3A). Interestingly, we found that while patients in the CheckPAC trial experienced an increase in CD3^+^ cells and CD8^+^ T cells in the blood at 8 weeks posttreatment initiation, the frequency of these populations did not change for patients in the TRIPLE-R trial (Fig. 6E). Next, we focused on monocytes, as these cells seem to be particularly susceptible to the immune modulatory properties of IL-6, as shown in our preclinical data. We used FlowSOM to identify different cell subsets among the CD3^-^ population. Monocytic metaclusters, annotated as described in the “Methods” section, were selected for analysis (Supplementary Fig. 3B, C). Patients in both studies had comparable frequencies of human leukocyte antigen (HLA)-DR^mid^ classical monocytes (CD14^+^ CD16^-^ HLA-DR^mid^) and nonclassical monocytes (CD14^−^ CD16^+^) at baseline (Supplementary Fig. 3D). However, the frequency of HLA-DR^-^ classical monocytes (CD14^+^ CD16^−^ HLA-DR^-^) at baseline was greater in the TRIPLE-R study, whereas the frequency of monocytes in an intermediate state (CD14^+^ CD16^+^ HLA-DR^hi^) at baseline was greater in the CheckPAC study (Supplementary Fig. 3D). Our analysis revealed that patients with high TGFβ-15-specific immunity at baseline in the CheckPAC study experienced a decrease in HLA-DR^mid^ classical monocytes after 8 weeks of treatment, whereas the frequency of this subset did not change over time for TRIPLE-R patients (Fig. 6F). These data suggest that the potential therapeutic benefit that strong TGFβ-specific immunity at baseline could confer in patients with PDAC treated with immune checkpoint inhibition and radiotherapy might be impaired due to alterations in the T-cell and monocytic compartments induced by IL-6R blockade.

## Discussion

Here, we demonstrated the importance of IL-6 for TGFβ-specific immunity in pancreatic cancer. Due to the previously described immune modulatory effects of TGFβ-specific T cells on fibroblasts in the tumor [10, 11], CAFs acquired an increased IL-6-secreting profile following treatment with the TGFβ vaccine. Interestingly, the therapeutic effect of the TGFβ-based immune modulatory vaccine was dependent on the presence of IL-6 in the TME. We propose that, under circumstances of vaccine-induced tumor cell destruction, IL-6 might have beneficial effects on the antitumor immune response. For example, when IL-6 signaling is compromised by IL-6R blockade, both CD4^+^ vaccine-specific T-cell immunity and the trafficking of T cells into tumors are impaired. The absence of IL-6 signaling in macrophages triggered the secretion of CCL9, resulting in the recruitment of myeloid cells, specifically monocytes, to the tumor. Consequently, myeloid cells became the predominant population within the TME. The frequency of proinflammatory TAMs decreased, whereas alternatively, activated TAMs became more suppressive and less prone to antigen presentation. We suggest that, consequently, the action of TGFβ-specific T cells within the TME became severely compromised, leading to the inability of the TGFβ vaccine to control tumor growth. We also addressed the importance of IL-6 for TGFβ-specific immunity in patients with pancreatic cancer. Individuals with high TGFβ-specific T-cell responses at baseline in the CheckPAC trial showed improved survival [7], which was accompanied by a decrease in classical monocytes and an increase in CD8^+^ T cells in peripheral blood after 8 weeks of treatment. However, when an anti-IL-6R antibody was added to the regimen in the TRIPLE-R trial, patients with a high TGFβ-specific T-cell response did not experience improved survival or changes in PBMC composition over time. We speculate that in the context of active immunotherapy-driven tumor destruction, blocking IL-6 signaling might compromise the potential therapeutic advantages associated with high baseline TGFβ-specific immunity in patients with PDAC receiving immune checkpoint inhibition and radiotherapy.

Extensive evidence supports the role of IL-6 in driving tumor progression by inducing cancer cell proliferation and survival while simultaneously suppressing antitumor immunity [3]. Preclinical studies have demonstrated that treatment with anti-IL-6 [23–28] or anti-IL-6R blocking antibodies [29] can increase the efficacy of immunotherapy, reduce tumor progression, and increase T-cell infiltration in several murine tumor models, including those of pancreatic cancer [23, 24]. Here, we demonstrated a completely opposite outcome of IL-6 targeting, as impairing IL-6 signaling in the TME with an IL-6R blocking antibody abolished the antitumor activity of the TGFβ vaccine in a murine model of pancreatic cancer. In line with our findings, Nejad et al. reported that the effect of an HPV16 E7-derived vaccine in a TC-1 tumor model was lost when IL-6 signaling was blocked with an anti-IL-6 antibody, an anti-IL-6R antibody, or both [6]. Similarly, a reduction in tumor growth in the B16-OVA tumor model was observed only when adoptive cell transfer of OT-1 effector T cells was combined with the administration of recombinant IL-6 [30]. These results contribute to the mounting evidence that demonstrates the dual role of IL-6 in cancer immunity and stresses that the contexts in which it promotes or suppresses antitumor immunity are not yet fully understood [3].

We showed that while vaccine-specific CD8^+^ responses were unaffected upon IL-6R blockade, the frequency of vaccine-specific CD4^+^ T cells in the spleen was considerably impaired. This could be attributed to the important role of IL-6 produced by dendritic cells in lymphoid organs in T-cell activation, expansion, and survival [3]. Moreover, an effective CD4^+^ T-cell priming and IFNγ response were shown to be dependent on IL-6 signaling [31, 32]. In the context of the TGFβ vaccine, IL-6R blockade reduced the abundance of tumor-infiltrating T cells and resulted in increased T-cell exhaustion. Evidence suggests that IL-6 signaling can promote T-cell infiltration into tumors by upregulating the expression of intercellular adhesion molecule (ICAM)-1 by endothelial cells in tumors [30].

It is generally believed that IL-6 modulates myeloid cells toward an immunosuppressive phenotype [5]. For example, IL-6 seems to increase mannose receptor and arginase-1 expression in macrophages [5, 33]. In addition, tumor-produced IL-6 has been shown to reduce the expression of MHC-II in myeloid cells in the TME [34], which can be recovered by IL-6R blockade [6]. In contrast, our data suggested that the absence of IL-6 signaling through IL-6R blockade led to a reduction in the population of proinflammatory TAMs and enhanced the suppressive phenotype of anti-inflammatory TAMs. Importantly, these changes seemed to occur within the context of active immune modulation and tumor destruction induced by the TGFβ vaccine. IL-6 sustains the expression of suppressor of cytokine signaling 3 (SOCS3) in macrophages, which retains the proinflammatory properties of IL-6 in this myeloid subset [35]. In line with our findings, preclinical studies have shown that tumor regression induced by cancer vaccines is dependent on IL-6 signaling in macrophages [6], as the reduction in SOCS3 expression in the absence of IL-6 polarized TAMs toward an anti-inflammatory state that significantly impaired the efficacy of cancer vaccines [6]. Interestingly, we found that IL-6R blockade drastically increased the frequency of CD8^+^ macrophages in tumors, which was negatively correlated with tumor T-cell infiltration. CD8^+^ macrophages have been identified in various central nervous system diseases, and although their specific properties and functions remain elusive, evidence suggests that they may contribute to the pathogenesis of inflammatory tissue damage [36–38]. Nevertheless, the role of CD8^+^ macrophages in tumor biology and within the context of IL-6 signaling remains to be elucidated.

Overall, further research is needed to elucidate the precise conditions under which IL-6 either boosts or impairs tumor immunity, with the ultimate aim of limiting IL-6-targeting therapies to scenarios in which it enhances the response to cancer immunotherapy.

## Material and methods

### Patients, patient characteristics, and patient samples

Patents with metastatic pancreatic cancer assessed in this study were included in the CheckPAC (NCT02866383) [7] or TRIPLE-R (NCT04258150) [8] phase 2 clinical trials conducted at the Department of Oncology, Copenhagen University Hospital, Herlev, Denmark. Patients in the CheckPAC study received nivolumab and ipilimumab in combination with stereotactic body radiotherapy (SBRT) [7]. Patients in the TRIPLE-R cohort were treated with nivolumab, ipilimumab, SBRT, and tocilizumab [8]. The clinical trials received approval from the relevant Ethics Committee and were conducted in accordance with the Declaration of Helsinki and Good Clinical Practice. The CheckPAC trial was approved by the Danish Ethics Committee (H-16031247) and the Danish Data Protection Agency (j.nr. 2012-58-0004; HGH-2016-112; I-Suite j.nr. 05088) [7]. The TRIPLE-R trial was approved by the Danish Ethics Committee (H-19087729) and the Danish Data Protection Agency (j.nr. P-2020-398) [8]. All patients provided written informed consent. The following clinical parameters at baseline were available for both studies: sex (female/male), age, Eastern Cooperative Oncology Group (ECOG) performance score (0/1), C-reactive protein (mg/L), number of leukocytes per mL, number of lymphocytes per mL, neutrophil-lymphocyte ratio, albumin (g/L), hemoglobin (mM) and prior treatments (≤2/>2). Peripheral blood mononuclear cells (PBMCs) from patients were isolated and cryopreserved as previously described [9]. The samples used for this study were collected at baseline and 8 weeks posttreatment initiation.

### Mice

Animal experiments were carried out at the animal facility of the Department of Oncology, Copenhagen University Hospital, Herlev, Denmark, adhering to guidelines set by the Federation of European Laboratory Animal Science Association (FELASA) and conducted under a license granted by the Danish Animal Experimentation Inspectorate (2021-15-0201-01001). Female C57BL/6 mice, aged 10–20 weeks, were either purchased from Taconic or bred in-house from a C57BL/6JBomTac lineage.

### Murine pancreatic cell lines and tumor models

The Pan02 cell line was obtained from the cell line biobank at the National Center for Cancer Immune Therapy (Denmark) and cultured in RPMI-1640 GlutaMAX (Gibco) supplemented with 10% heat-inactivated fetal bovine serum (FBS, Gibco) and 1% penicillin/streptomycin (P/S, Gibco). The KPC cell line, which was established from primary tumors from KPCY mice, was purchased from Kerafast (2838c3) and cultured in high-glucose DMEM with GlutaMAX pyruvate (Gibco) supplemented with 10% FBS and 1% P/S. Cancer cells were expanded in vitro, and then, the mice were subcutaneously (s.c.) inoculated with 5 × 10^5^ cells in the right flank. Upon tumor palpability, the mice were assigned to treatment groups by stratified randomization on the basis of tumor volume. A digital caliper was used to measure the tumor dimensions three times a week. The tumor volume was calculated as 0.5 × length × width^2.

### Peptides

The following murine TGFβ1-derived peptides were used for this study: mTGFβ (mTGFβ)−18–32 (15mer, LLVLTPGRPAAGLST), mTGFβ − 215–223 (9mer, QGFRFSAHC), mTGFβ − 282–289 (8mer, TNYCFSST) and mTGFβ − 334–342 (9mer, TQYSKVLAL), which constitute the TGFβ vaccine [10]. Peptides were dissolved in 20 mM dimethyl sulfoxide (DMSO), with the exception of mTGFβ (mTGFβ)−18–32, which was reconstituted in 2 mM H_2_O. For experiments with human PBMCs, the TGFβ1-derived peptide TGFβ-15 (REAVPEPVLLSRAELRLLRL) and the *Clostridium tetani-*derived “tetanus” peptide (AQYIKANSKFIGITEL) were used following reconstitution with 10 mM dimethyl sulfoxide (DMSO). Peptides were purchased from Schäfer (>90% purity).

### Treatments

The mice were treated with the TGFβ vaccine as previously described [10]. In brief, the mice were vaccinated s.c. at the base of the tail, with two emulsions per treatment point. One contained 100 µg of the murine TGFβ1-derived peptide mTGFβ-18–32. The other group comprised 50 µg of each of the following peptides: mTGFβ − 215–223, mTGFβ − 282–289, and mTGFβ − 334–342. The vaccines were formulated by creating an emulsion of the peptide solution with Montanide ISA 51 VG (Seppic) at a 1:1 ratio. For control vaccinations, mice were treated s.c. at the base of the tail with an emulsion generated by emulsifying water with Montanide ISA 51 VG at a 1:1 ratio. For the tumor studies, the mice were vaccinated when the tumors emerged (day 8 postinoculation) and 7 days later (day 15 postinoculation). For cocultures with T cells from tumor-free, vaccinated mice, vaccinations were performed on days 0 and 7. To block IL-6R in vivo, the InVivoMAb anti-mouse IL-6R blocking antibody was purchased from BioXCell (clone 15A7). Each mouse received 200 µg of antibody in 50 µL of PBS s.c. next to the tumor per mouse on day 8 postinoculation and every 3rd, 4th day for a total of six injections. As a control, 50 µL of PBS was administered at the same location and following the same schedule.

### Generation of tumor-conditioned media

Tumor-conditioned media (TCM) was generated as previously described [10]. Briefly, Pan02 tumor-bearing mice that were either untreated or treated with the TGFβ vaccine were euthanized on days 25–30 postinoculation. Tumors were collected, and single-cell suspensions were generated. A total of 1 × 10^5^ cells were seeded per well in a U-shaped bottom plate. The cell culture supernatant, termed TCM, was collected after a 48 h incubation.

### Cell sorting

Cancer-associated fibroblasts (CAFs) were isolated from KPC tumors. In brief, KPC tumor-bearing mice were euthanized 14–18 days postinoculation. Tumors were harvested, cut into smaller pieces, and digested for 30 min at 37 °C and 300 rpm in digestion buffer (RPMI-1640 GlutaMAX supplemented with 1% P/S, 1 mg/mL collagenase type IV (Sigma‒Aldrich), 75 µg/mL DNase I (Sigma‒Aldrich), and 5 mM CaCl_2_). CAFs were sorted on the basis of the marker CD90.2 via the Mouse Tumor-Associated Fibroblast Isolation Kit from Miltenyi Biotec, according to the manufacturer´s instructions. T cells were isolated from the spleens of vaccinated mice. Briefly, 1 week after the last vaccination, the mice were sacrificed, and the spleens were harvested. Single-cell suspensions were generated by processing the spleens through a 70 µm cell strainer and lysing red blood cells with RBC lysis buffer (QIAGEN). T cells were sorted with the Mouse Pan T-Cell Isolation Kit II from Miltenyi Biotec following the manufacturer’s instructions. Monocytes were sorted from the bone marrow of untreated, tumor-free mice. In brief, the mice were euthanized, and the femurs and tibias were collected. The bone marrow was harvested by flushing the bones with PBS. Single-cell suspensions were generated by processing the sample through a 70 µm cell strainer. Monocytes were isolated from Miltenyi Biotec via the Mouse Monocyte Isolation Kit (BM) according to the manufacturer´s guidelines.

### Cocultures

Sorted CAFs were cultured in DMEM supplemented with 10% FBS and 2% P/S for in vitro expansion. A total of 8.5 × 10^4^ CAFs were cultured with 1 × 10^6 ^T cells (1:12 CAF:T-cell ratio) per well in a final volume of 200 µL of DMEM, 10% FBS, and 1% P/S in a U-shaped bottom 96-well plate. Following a 48 h incubation, the cells were collected for flow cytometry, and the supernatant was saved for enzyme-linked immunosorbent assay (ELISA).

### ELISA

The IL-6 concentration in the medium and in the coculture supernatant was determined via mouse IL-6 DuoSet ELISA (R&D Systems, DY406) following the manufacturer´s instructions. The optical density was determined with an Epoch Microplate Spectrophotometer (BioTek Instruments) via Gen5 software.

### ELISpot

The presence of peptide-specific T cells in murine spleens or in human PBMCs was assessed via IFNγ enzyme-linked immunospot (ELISpot), as previously described [9, 10]. ELISpot plates (Mabtech) were coated with an unconjugated anti-IFNγ antibody (Mabtech, AN18 for mouse, 1-D1K for human) diluted to 12 µg/mL or 7.5 µg/mL for mouse or human, respectively, in PBS and incubated overnight. A total of 8 × 10^5^ murine splenocytes in 200 µL of RPMI-1640 supplemented with 10% FBS, 1% P/S, or 2 × 10^5^ PBMCs in 200 µL of X-VIVO 15 (Life Science) were plated per well in triplicate. Human PBMCs were stimulated in vitro with TGFβ-15 and cultured for approximately 10 days before being used in the assay, as previously described [9, 22]. Following a 24 h incubation at 37 °C, the plates were rinsed with PBS. The cells were stimulated with peptide solution at a working concentration of 5 µM. The general response to the TGFβ vaccine was evaluated by stimulation with a peptide pool comprising all five peptides that conform the TGFβ vaccine to a working concentration of 5 µM per peptide. Biotinylated anti-IFNγ antibodies (Mabtech, R4-6A2 for mouse, 7-b6-1 for human) diluted to 1 µg/mL or 0.75 µg/mL for mouse or human, respectively, in ELISpot buffer (PBS, 0.5% bovine serum albumin, and NaN3) were used as secondary antibodies. The plates were incubated for 2 h at room temperature, followed by a PBS-washing step. Streptavidin-ALP (Mabtech) diluted 1:1000 in ELISpot buffer was added, and the plates were incubated for 1 h at room temperature. Unbound enzyme was washed off with PBS. The assay was developed following a 1–5 min incubation at room temperature with the enzyme-substrate BCIP/NBT (Mabtech). The reaction was stopped with tap water. A CTL ImmunoSpot S6 Ultimate-V analyzer with ImmunoSpot software (v5.1) was used to count the spots. Specific responses are expressed as the difference between the average number of spots in peptide-stimulated wells and those in unstimulated wells. TGFβ-15-specific responses at baseline were classified as high (TGFβ-15^high^) or low (TGFβ-15^low^) following the cutoff of 50 specific cells per 250,000 plated cells, as described by Mortensen et al. [9]. We assessed TGFβ-15-specific and tetanus-specific responses in all 25 patients (10 with TGFβ-15^high^ and 15 with TGFβ-15^low^) included in the TRIPLE-R study [8] from whom PBMCs at baseline were available.

### Flow cytometry

Zombie Aqua (BioLegend, 423101) was used as a viability marker for flow cytometry of murine samples. Fc receptors were preblocked with mouse FcR blocking reagent (1:10; Miltenyi Biotec). The following antibodies were used to assess the mean fluorescence intensity (MFI) of IL-6 and IL-6R in CAFs from untreated Pan02 tumors: CD45-FITC (BioLegend, 103108), CD90.2-BV605 (BioLegend, 140317), CD8-BB700 (BD Pharmingen, 566409), CD4-BV421 (BioLegend, 100438), CD11b-APC/Cy7 (BioLegend, 101226), F4/80-PE (BioLegend, 123110), CD126 (IL6Ra)-APC (BioLegend, 160408) and IL-6-APC (BioLegend, 504508). CAFs were gated as live CD45^−^ CD90.2^+^ cells. CD4^+^ T cells were gated as live CD45^+^ CD4^+^ cells. CD8^+^ T cells were gated as live CD45^+^ CD8^+^ cells. Myeloid cells were gated as live CD45^+^ CD11b^+^ cells. Tumor-associated macrophages (TAMs) were gated as live CD45^+^ CD11b^+^ F4/80^+^ cells. The following antibodies were used to evaluate changes in the IL-6 MFI in CAFs after culture with T cells: CD45-PE/Cy7 (BioLegend, 103114), CD90.2-BV605 (BioLegend, 140317) and IL-APC (BioLegend, 504508). CAFs were gated as live CD45^−^ CD90.2^+^ cells. The following antibodies were used to investigate changes in the T-cell compartment in Pan02 tumors across treatment groups: CD45-BV605 (BioLegend, 103140), CD3-AF700 (BioLegend, 100216), CD8-BB700 (BD Pharmingen, 566409), CD4-BV421 (BioLegend, 100437), FoxP3-APC (eBioscience, 17-5773-82), CD25-BV786 (BD Horizon, 564023) and PD-1-PE (BioLegend, 109104). T cells were gated as CD45^+^ CD3^+^ T cells. CD4^+^ T cells were gated as CD45^+^ CD3^+^ CD4^+^ T cells. CD8^+^ T cells were gated as CD45^+^ CD3^+^ CD8^+^ T cells. Regulatory T cells (Tregs) were gated as CD45^+^ CD3^+^ CD4^+^ Foxp3^+^ CD25^+^ cells. The following antibodies were used to assess changes in the myeloid subset in Pan02 tumors across treatment groups and changes in the phenotype of BMDMs differentiated in the absence or presence of IL-6: CD45-BV605 (BioLegend, 103140), CD3-AF700 (BioLegend, 100216), CD11b-Pacific blue (BioLegend, 101223), F4/80-FITC (BioLegend, 123108), MR (CD206)-PE/Cy7 (BioLegend, 141719), MHC-II-APC/Cy7 (BioLegend, 107627), Arg1-PE (R&D Systems, IC5868P), PD-L1-APC (BD Biosciences, 564715) and CD8a-PerCP/Cy5.5 (Pharmingen, 551162). Myeloid cells were gated as live CD45^+^ CD3^−^ CD11b^+^ cells. TAMs or BMDMs were gated as live CD45^+^ CD3^−^ CD11b^+^ F4/80^+^ cells.

Characterization of human PBMCs with flow cytometry at baseline (w0) and 8 weeks posttreatment (w8) was performed on all patients from whom PBMCs were available at both timepoints (*n* = 10 patients for the CheckPAC trial and *n* = 9 for the TRIPLE-R trial). NIR (Invitrogen, L34993) was used as a live marker for flow cytometry with human PBMCs. The following antibodies were used to assess changes in the T-cell compartment in human PBMCs: CD3-BV786 (BD Biosciences, 563800), CD8-PE-CF594 (BD Biosciences, 566850) and CD4-BV510 (BD Biosciences, 562970). T cells were gated as live CD3^+^ cells. The following antibodies were used to assess changes in myeloid subsets in human PBMCs: CD3-BV786 (BD Biosciences, 563800), CD14-PE-CF594 (BD Biosciences, 562335), CD16-BV650 (BD Biosciences, 563692), HLA-DR-PerCP-Cy5.5 (BD Biosciences, 560652), CD123-BV605 (BD Biosciences, 564197), CD1c-APC (BioLegend, 331524), CD11c-PE (BioLegend, 371504), CD33-BV510 (BD Biosciences, 563257), CD56-FITC (BD Biosciences, 345811), NKG2a-BV421 (BD Biosciences, 747924), and CD19-BV711 (BD, 563038). Monocytic populations were identified via high-dimensional analysis of flow cytometry data from human PBMCs with Cytobank. For the staining of intracellular proteins, the samples were fixed and permeabilized with eBioscience Fixation/Permeabilization Concentrate, Diluent, and 10X Buffer (Invitrogen) following the manufacturers’ instructions. Data acquisition was performed on an ACEA NovoCyte Quanteon (Agilent), and the data were analyzed via FlowJo V.10.6.1 (Tree Star).

### High-dimensional data analysis of flow cytometry data

High-dimensional analysis of flow cytometry data from human PBMCs stained with a myeloid panel and murine tumors stained with a myeloid panel was performed via Cytobank (https://cytobank.org) [39]. For data from human PBMCs, uniform manifold approximation and projections (UMAP) analysis was performed on manually gated live CD3^−^ cells, with 50,000 events per sample, 15 neighbors, a minimum distance of 0.01 and collapsed outliers (Z score > 3) with normalized scales. The following markers were used to generate the UMAP: CD14, CD16, HLA-DR, CD123, CD1c, CD11c, CD33, CD56, NKG2a and CD19. The data were then fed into the FlowSOM clustering algorithm, which samples 50,000 events per sample. Hierarchical consensus clustering with 10 iterations and 100 clusters was used after scale normalization, resulting in a total of 30 metaclusters. Metaclusters were annotated on the basis of median fluorescence intensity (MFI) heatmaps generated in Cytobank. Clusters with less than 1% frequency were excluded from the analysis, and clusters that would receive the same annotation on the basis of heatmap marker expression were combined if one of them constituted <5% of the live cells. The CD14^+^ CD16^−^ HLA-DR^-^ clusters were annotated as HLA-DR^−^ classical monocytes, the CD14^+^ CD16^−^ HLA-DR^mid^ clusters were annotated as HLA-DR^mid^ classical monocytes, the CD14^+^ CD16^+^ HLA-DR^hi^ clusters were annotated as monocytes in an intermediate state, and the CD14^−^ CD16^+^ clusters were annotated as nonclassical monocytes. Clusters corresponding to the monocyte population (*n* = 4) were selected for downstream analysis. For data from murine tumors, UMAP analysis was performed on manually gated live CD45^+^ CD3^−^ cells, sampling 1930 events per sample, with 15 neighbors and a minimum distance of 0.01. The following markers were used to generate the UMAP: CD11b, F4/80, MR (CD206), ARG1, MHC-II and PD-L1. The data were then fed into the FlowSOM clustering algorithm, which samples 1930 events per sample. Hierarchical consensus clustering with 10 iterations and 49 clusters was used after scale normalization, resulting in a total of 6 metaclusters. Metaclusters were annotated on the basis of MFI heatmaps generated via the pheatmap (V1.0.12) R package. CD11b^−^ clusters were annotated as non-myeloid cells. CD11b^+^ clusters were annotated as myeloid cells. F4/80^+^CD11b^+^ clusters were annotated as macrophages. Macrophage clusters characterized as MR^low^ ARG1^low^ MHC-II^+^ or MR^high^ ARG1^high^ MHC-II^-^ were annotated as proinflammatory or anti-inflammatory macrophages, respectively.

### Immunofluorescence

Pancreases were fixed in 4% paraformaldehyde (PFA) for 24 h at room temperature (RT) and subsequently incubated with 70% ethanol (VWR Chemicals; 20824.365) until paraffin embedding. Sections (4 μm) were mounted on Superfrost Plus slides (Fisher Scientific; 10149870). The paraffin-embedded samples were rehydrated with xylene and ethanol with increasing concentrations of water. After deparaffinization, 0.5% PBS-Tween-20 was used in all washing steps. Antigen retrieval was performed by boiling for 20 min with Tris-EDTA buffer at pH 9.0. The samples were blocked with 1% normal donkey serum (Jackson ImmunoResearch, 017-000-121) and later incubated with primary antibodies (rabbit anti-Keratin7 (Abcam, ab181598, 1:200) and goat anti-IL6 (Santa Cruz, sc-1265, 1:200)) overnight at 4 °C. The sections were then incubated with secondary antibodies (Cy3 donkey anti-rabbit (Jackson ImmunoResearch, 711-166-152, 1:500), FITC donkey anti-goat (Jackson ImmunoResearch, 705-546-147, 1:500)) and DAPI (Sigma‒Aldrich; D9542) for 1 h at room temperature. The samples were mounted with Vectashield® Mounting medium (Vector Laboratories; H-1000). An Olympus® ScanR screening microscope was used for imaging. Image processing and quantification were performed via QuPath software (v.0.4.0) (https://qupath.github.io/). For each sample, 2 or 3 cuts at different depths were stained and imaged (9 tiles per cut). Tiles with staining artifacts or a low number of cells were excluded from the analysis. The cells were detected with the built-in “Cell detection” tool using DAPI. Positive cells were classified with the “Set intensity classification” built-in tool.

### RNA extraction

RNA was isolated from bone marrow-derived macrophages and Pan02 tumors. The macrophages were allowed to stand down, the supernatant was removed, and the pellet was stored at −80 °C. Pan02 tumors (≤20 mg) were stored in RNAlater (Invitrogen) at −80 °C. For RNA extraction from Pan02 tumors, the material was transferred to RLT buffer (QIAGEN) and mechanically homogenized via a Tissue Lyser (QIAGEN). RNA extraction was performed via the RNeasy Plus Mini Kit (QIAGEN) according to the manufacturer’s protocol. The RNA concentration was quantified with a NanoDrop 2000 Spectrophotometer (Thermo Scientific).

### qRT‒PCR

Complementary DNA (cDNA) synthesis from 1 µg of RNA was performed via an iScript cDNA synthesis kit (Bio-Rad) following the manufacturer’s instructions. cDNA was diluted 1:3. Reverse transcription‒quantitative PCR (RT‒qPCR) was performed in technical triplicate in an AriaMX Real-Time PCR System with a LightCycler 480 Probes Master (Roche Diagnostics) and the following TaqMAn gene expression assay probes (Life Technologies): *Hprt1* (Mm00446968_m1), MCH-I *H2-D1/K1* (Mm04208017_mH), MHC-II *H2-Ab1* (Mm00439216_m1), *Ccl6* Mm01302419_m1), *Ccl8* (Mm01297183_m1), *Ccl9* (Mm00441260_m1), *Pf4(*Mm00451315_g1), *Alox12e* (Mm00521331_m1), *Fcer1a* (Mm00438867_m1) and *Arg1* (Mm00475988_m1). The data were normalized to Hprt1 as a housekeeping gene and analyzed via the 2−dCT method.

### RNA sequencing

Bulk RNA sequencing (RNAseq) was performed as previously described10. Briefly, 500 ng of RNA from Pan02 tumors with an RNA integrity number (RIN) score above 7 was enriched for polyadenylated mRNA via Oligo dT beads (NEBNext). The subsequent steps included fragmentation, random-primed cDNA synthesis (NEBNext), PCR-mediated indexing (NEBNext), size selection, and quantification (KAPA, Roche). The Illumina NovaSeq 6000 platform was utilized for cDNA library paired-end sequencing. The alignment to the GRCm39 mouse reference genome and quantification of reads were conducted following previously described procedures10, employing STAR (V.2.7.9), featureCounts within the subread package (V.2.0.3), and Ensembl gene transcripts (GRCm39.104.gtf). The RNAseq counts were VST (variance stabilizing transformation)-normalized. Differential gene expression was performed via the DESeq2 package (V.1.30) with a cutoff of adjusted *p*-value < 0.05 and an absolute log2-fold change >0.585. The RNA-seq data have been deposited in the GEO repository under the accession number GSE263363. The enhanced Volcano R package (V.1.8.0) was used to generate volcano plots. Gene Ontology (GO) analysis for biological processes of the differentially expressed genes was performed via the enrichGO functions of the cluster profile (V.4.6.2) package in R. GO terms related to cancer immunity was selected and manually classified into the following 10 categories: myeloid cell chemotaxis/migration, lymphoid cell chemotaxis/migration, response to cytokines/chemokines, phagocytosis, B-cell function, and immunoglobulins, immune activation and immune processes, myeloid immunity, regulation of cytokinesis, metabolism, ECM, and categories related to cancer immunity. GO terms were organized into a network and subsequently plotted via the emapplot function of the enrich plot (V.1.18.4) package in R. Cell-type deconvolution of the bulk samples to infer enrichment scores was performed on transcripts per million normalized counts via mMCP-counter [40], implemented in the R package immunedeconv (v2.1.0) [41].

### scRNAseq data from public repositories

Single-cell RNA sequencing (scRNAseq) analyses were performed via a publicly available scRNAseq atlas of >70 samples and 136,163 cells from human PDAC tumors generated by Chijimatsu et al. [12]. We downloaded the Seurat object containing normalized expression data and the annotated metadata, which was used to show the clusters identifying different cell types. Visualization of the scRNA-seq data was performed via the FeaturePlot and DotPlot functions from the Seurat R package (V.5.0.1).

### Generation of bone marrow-derived macrophages

The mice were sacrificed, and the femurs and tibias were retrieved. The bone marrow was collected by flushing the bones with PBS. Single-cell suspensions were obtained by passing the sample through a 70 µm cell strainer. To generate bone marrow-derived macrophages (BMDMs), 4 × 10^6^ bone marrow-derived cells were cultured in 10 mL of DMEM with 20% FBS and 1% P/S supplemented with 20 ng/mL human M-CSF (PeproTech) in a Petri dish. On days 3 and 5, the medium was completely removed, and 10 mL of fresh medium supplemented with 20 ng/mL human M-CSF was added to each Petri dish. On day 6, the BMDMs were harvested for downstream analysis. To evaluate the effect of IL-6 on BMDMs, IL-6 (PeproTech) was added to the medium at a concentration of 20 ng/mL.

### Monocyte migration assay

A total of 900 µL of DMEM supplemented with 10% FBS and 1% P/S containing 70 ng/mL CCL2 (PeproTech) was added to each well of a 24-well plate. A 5 µm membrane insert (Millicell Sigma, PTMP24H48) was placed hanging in each well. A total of 1.25 × 10^5^ monocytes isolated from the bone marrow of untreated, tumor-free mice were placed in 200 µL of DMEM supplemented with 10% FBS and 1% P/S inside the insert. The cell cultures were incubated and allowed to migrate for 2 h or overnight at 37 °C. After this time, 800 µL of the media outside the chamber were harvested, passed through, and resuspended in 100 µL of FACS buffer. A total of 90 µL was acquired on an ACEA NovoCyte Quanteon (Agilent), and the number of events was recorded. The results are expressed as the percentage of migrated cells to the initial cell count in the insert.

### Cell length measurement

BMDM length was determined via QuPath. Microscopy images were imported into the program, and an area of 2100 pixels was randomly selected. Lines across the length of each cell within the selected area were manually drawn. The length of the pixel corresponding to each line was exported.

### Data visualization

The data were visualized via GraphPad Prism (V.8) or the following R packages: ggplot2 (V.3.3.5) and pheatmap (V1.0.12). ggpubr (V.0.6.0), survival (V.3.5.5), ggsurvfit (V.1.0.0), survminer (V.0.4.9) and gtsummary R packages (V.1.7.2).

### Statistical analysis

For preclinical data, unpaired, two-tailed *t*-tests performed in GraphPad Prism (v8) were used to assess statistical significance, unless otherwise stated. Statistical comparisons of the tumor growth curves were conducted with TumGrowth software [42] (https://kroemerlab.shinyapps. io/TumGrowth), default settings, and a Bonferroni adjustment for correction for multiple comparisons. Linear regressions were performed with GraphPad Prism (v8). For human data, paired or unpaired two-tailed *t*-tests were performed with the ggpubr R package (V.0.6.0). Patient characteristics at baseline across clinical trials were compared via Fisher’s exact test for categorical variables and the Wilcoxon rank sum test or Wilcoxon rank sum exact test for noncategorical variables via the gtsummary R package (V.1.7.2). Survival analyses for all patients in the CheckPAC trial [7] based on TGFβ-15-specific immunity at baseline were previously published [9]. Here, we assessed survival on the basis of TGFβ-15-specific immunity at baseline only for patients in the CheckPAC trial who received radiotherapy, nivolumab, and ipilimumab to enable comparison with those in the TRIPLE-R trial [8]. Survival analysis for the CheckPAC trial was performed on all 17 patients (13 with TGFβ-15^high^ and 4 with TGFβ-15^low^) for whom survival data and TGFβ-15-specific immunity data at baseline were available. Survival analysis for the TRIPLE-R trial was performed on all 22 patients (10 with TGFβ-15^high^ and 12 with TGFβ-15^low^) for whom survival data and TGFβ-15-specific immunity data at baseline were available. Overall survival (OS) and progression-free survival (PFS) were assessed via a log-rank test with the survival (V.3.5.5) and ggsurvfit (V.1.0.0) R packages. PFS was determined by investigator assessment according to the RECIST v1.1 guidelines. *p < 0.05, **p < 0.01, ***p < 0.001, ****p < 0.0001. The data are presented as the means ± SEMs unless otherwise stated.

## Supplementary information

Supplementary information

## Data Availability

Data is available upon reasonable request.
