# Supplementary material for: The antitumor activity of TGFβ-specific T cells is dependent on IL-6 signaling
Source: Cell Mol Immunol. 2024 Dec 9;22(1):111–26. doi: 10.1038/s41423-024-01238-7 (PMC11685413; doi:10.1038/s41423-024-01238-7)
Supplement: Supplementary file 1 — Supplementary information [file 41423_2024_1238_MOESM1_ESM.pdf]

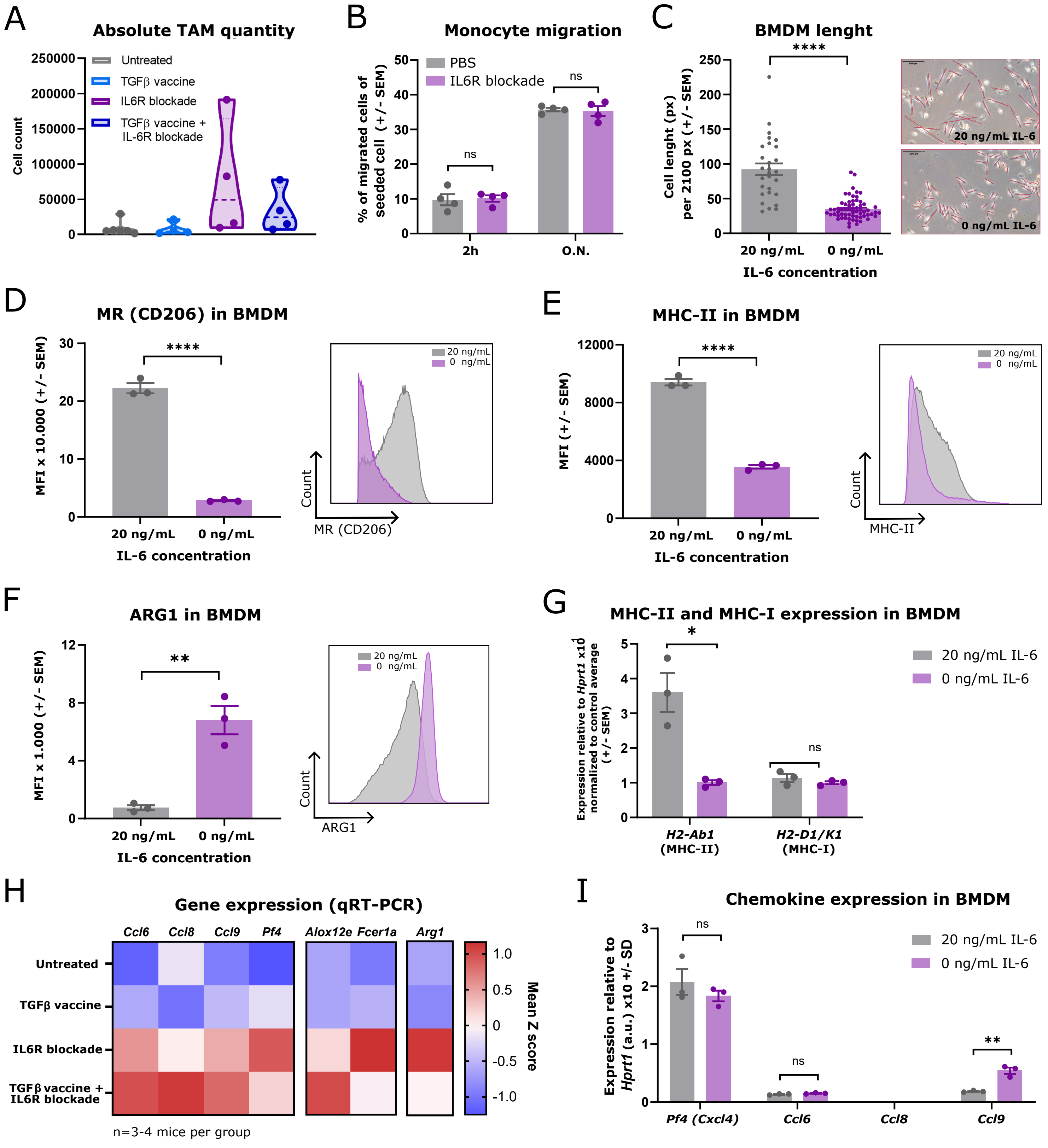

Supplementary Fig. 1. **While the lack of IL-6 signaling does not impair monocyte migration, it does affect the phenotype and chemokine-expression profile of bone marrow-derived macrophages. (A)** Absolute quantity of tumor-associated macrophages (TAM) in the tumor of Pan02-bearing mice that were treated with PBS, the TGFβ vaccine, IL-6R blockade or the combination of the TGFβ vaccine and IL-6R blockade, assayed by flow cytometry. **(B)** Percentage of monocytes isolated from the bone marrow of mice that were treated with PBS or an anti-IL-6R blocking antibody that migrated towards CCL2 as a chemoattractant compared to initial number of seeded monocytes after a 2-hour or an overnight incubation. **(C)** Cell length of bone marrow-derived macrophages (BMDM) that were differentiated from progenitors in the bone marrow in the presence (20 ng/mL) or absence of IL-6. **(D)** Mannose receptor (MR) mean fluorescence intensity (MFI) in BMDM that were differentiated from progenitors in the bone marrow in the presence (20 ng/mL) or absence of IL-6, assessed by flow cytometry. **(E)** MHC-II MFI in BMDM that were differentiated from progenitors in the bone marrow in the presence (20 ng/mL) or absence of IL-6, assessed by flow cytometry. **(F)** Arginase-1 (ARG1) MFI in BMDM that were differentiated from progenitors in the bone marrow in the presence (20 ng/mL) or absence of IL-6, assessed by flow cytometry. **(G)** *H2-Ab1* (MHC-II) and *H2-D1/K1* (MHC-I) expression relative to the housekeeping gene *Hprt1* in BMDM that were differentiated from progenitors in the bone marrow in the presence (20 ng/mL) or absence of IL-6, assessed by qRT-PCR. **(H)** *Ccl6, Ccl8, Ccl9, Pf4* (*Cxlc4*)*, Alox12e, Fcer1a* and *Arg1* expression relative to the housekeeping gene *Hprt1* in the tumor of mice that were untreated, treated with the TGFβ vaccine, an anti-IL-6R blocking antibody, or the combination of both, assessed by qRT-PCR. The average expression level per group was calculated and column mean centered (z-score). **(I)** *Pf4* (*Cxlc4*)*, Ccl6, Ccl8* and *Ccl9* expression relative to the housekeeping gene *Hprt1* in BMDM that were differentiated from progenitors in the bone marrow in the presence (20 ng/mL) or absence of IL-6, assessed by qRT-PCR. For (A-F) and (H), data presented as average +/- SEM. *p<0.05 and **p<0.01 according to an unpaired two-tailed t test.

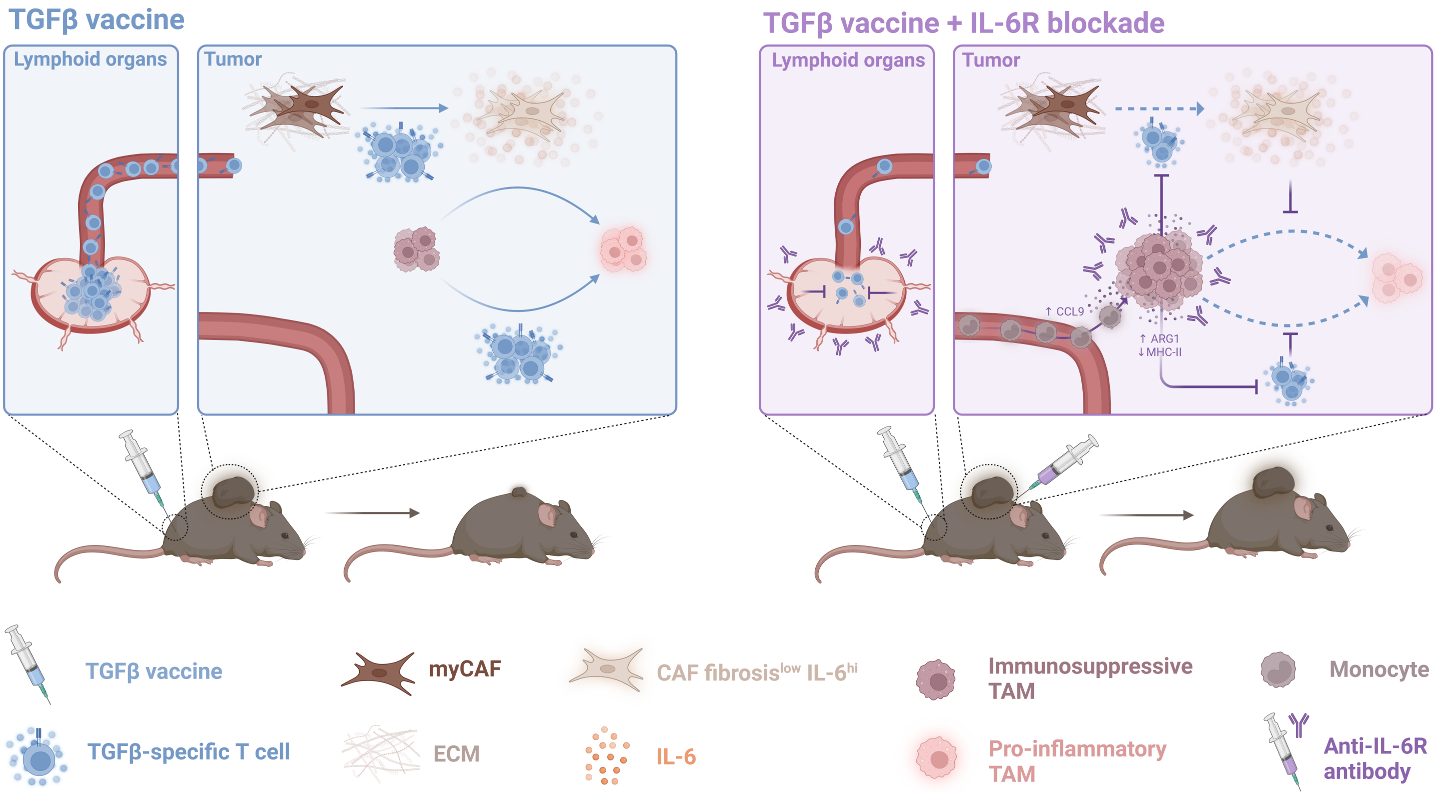

Supplementary Fig. 2. Proposed mechanisms underlying the role of IL-6 in the anti-tumor effect of the TGFβ vaccine under circumstances of vaccine-induced tumor regression. (Left) We hypothesize that in the presence of IL-6 signaling, TGFβ-specific T cells are expanded in lymphoid organs following vaccination with the TGFβ vaccine. These T cells can home to the tumor, where they can directly and indirectly target immunosuppressive tumor-associated macrophages (TAMs) and polarize them towards a pro-inflammatory phenotype, as previously described[10,14]. In addition, TGFβ-specific T cells can directly and indirectly target myofibroblastic cancer-associated fibroblasts (myCAFs), shifting their phenotype towards a reduced extracellular-matrix (ECM) depositing state, as previously described[10,11], together with a polarization towards a high IL-6 secreting phenotype, as reported here. As a result, the TME becomes less desmoplastic and less immunosuppressive, enabling the anti-tumor immunity to become more effective, leading to a control in tumor growth in a murine model of pancreatic cancer. (Right) In the absence of IL-6 signaling due to IL-6R blockade, the development of vaccine-specific $CD4^+$ TGFβ-specific T cells in lymphoid organs upon vaccination with the TGFβ vaccine is impaired, which results in a reduced infiltration of TGFβ-specific T cells in the tumor. Upon IL-6R blockade, the abundance of pro-inflammatory TAMs is reduced and TAMs become more immunosuppressive. For instance, they can express higher levels of arginase-1 (ARG1) and lower levels of major histocompatibility complex (MHC)-II, further inhibiting the anti-tumor activity of tumor-specific T cells as well as the immunomodulatory properties of TGFβ-specific T cells on CAFs and TAMs. In the absence of IL-6, TAMs secrete higher levels of CCL9, which results in the recruitment of monocytes in to the TME, which can differentiate into TAMs, thereby increasing the percentage of suppressive myeloid cells in the tumor. As a result, the anti-tumor effect of TGFβ-specific T cells in the TME is strongly suppressed, leading to a loss in tumor growth control. Created with Biorender.com.

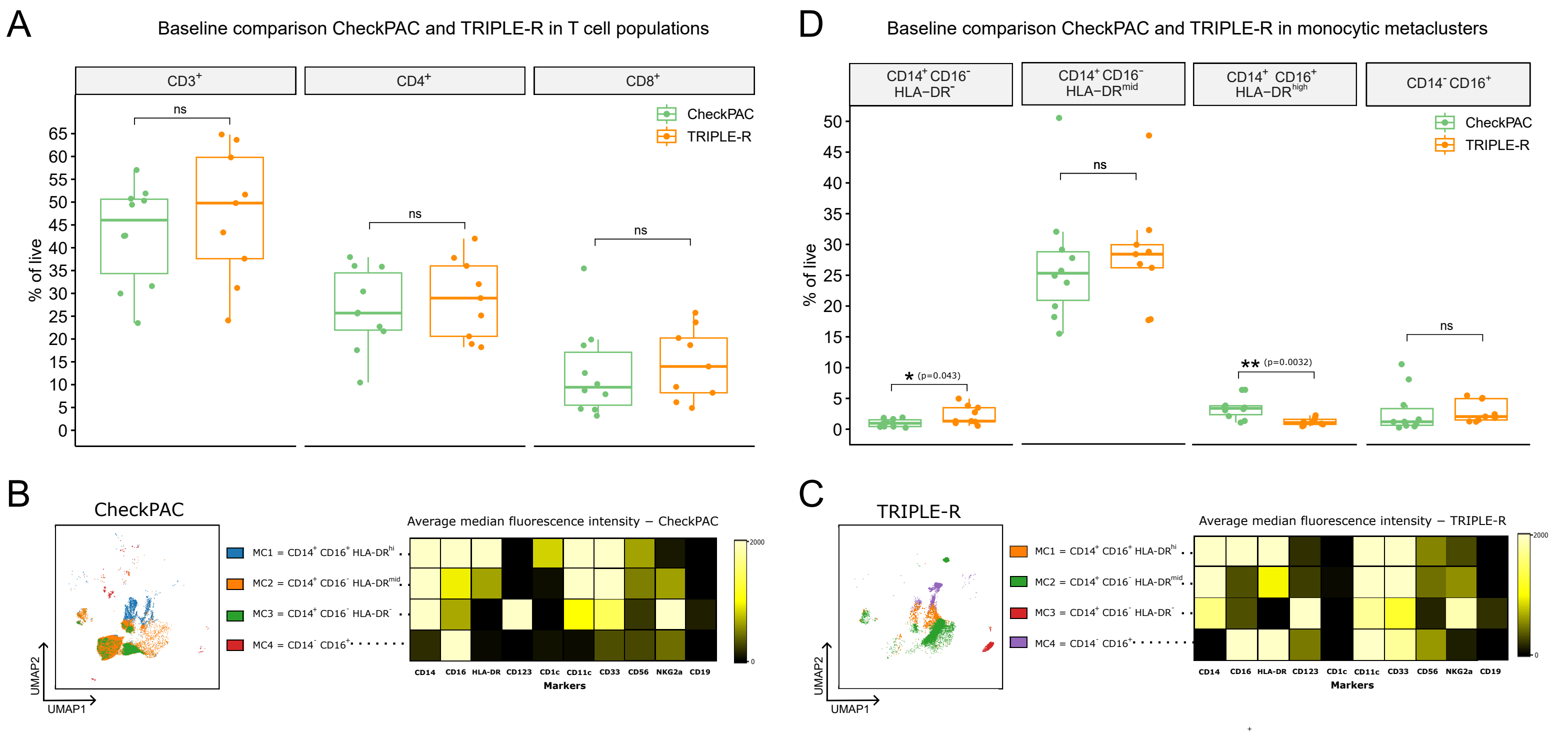

Supplementary Fig. 3. Comparison of baseline PBMC T cell and monocytic composition and identification of monocytic metaclusters using FlowSOM in patients with high TGFβ-15-specific response in the CheckPAC and TRIPLE-R trials. (A) Percentage of CD3^{+} cells, CD4^{+} T cells and CD8^{+} T cells of live cells in peripheral blood mononuclear cells (PBMCs) at baseline for patients that were treated with both immune checkpoint inhibitors (ipilimumab and nivolumab) and that had a high TGFβ-15-specific T cell response at baseline in the CheckPAC trial (n = 10 patients) and for patients with high TGFβ-15-specific T cell response in the TRIPLE-R trial (n = 9 patients). **(B, left)** UMAP displaying the four monocytic metaclusters identified using FlowSOM unsupervised clustering algorithm in Cytobank platform on the live CD3^{-} population for patients with a high TGFβ-15-specific T cell response in the CheckPAC trial that were treated with both immune checkpoint inhibitors (ipilimumab and nivolumab) (n = 10 patients). UMAP from a representative patient is shown. **(B, right)** Heatmap displaying the mean fluorescence intensity (MFI) of several myeloid markers used for the FlowSOM analysis shown in **(B, left)** across the four monocytic metaclusters, which correspond to: CD14^{+} CD16^{-} HLA-DR^{-} (HLA-DR^{-} classical monocytes), CD14^{+} CD16^{-} HLA-DR^{mid} (HLA-DR^{mid} classical monocytes), CD14^{+} CD16^{+} HLA-DR^{hi} (monocytes in an intermediate state) and CD14^{-} CD16^{+} (non-classical monocytes). **(C, left)** UMAP displaying the four monocytic metaclusters identified using FlowSOM unsupervised clustering algorithm in Cytobank platform on the live CD3^{-} population for patients with high TGFβ-15-specific T cell response in the TRIPLE-R trial (n = 9 patients). UMAP from a representative patient is shown. **(C, right)** Heatmap displaying the MFI of several myeloid markers used for the FlowSOM analysis shown in **(C, left)** across the four monocytic metaclusters, which correspond to: CD14^{+} CD16^{-} HLA-DR^{-} (HLA-DR^{-} classical monocytes), CD14^{+} CD16^{-} HLA-DR^{mid} (HLA-DR^{mid} classical monocytes), CD14^{+} CD16^{+} HLA-DR^{hi} (monocytes in an intermediate state) and CD14^{-} CD16^{+} (non-classical monocytes). **(D)** Percentage of the following monocytic metaclusters: CD14^{+} CD16^{-} HLA-DR^{-} (HLA-DR^{-} classical monocytes), CD14^{+} CD16^{-} HLA-DR^{mid} (HLA-DR^{mid} classical monocytes), CD14^{+} CD16^{+} HLA-DR^{hi} (monocytes in an intermediate state) and CD14^{-} CD16^{+} (non-classical monocytes) of live cells in PBMCs at baseline for patients that were treated with both immune checkpoint inhibitors (ipilimumab and nivolumab) and that had a high TGFβ-15-specific T cell response at baseline in the CheckPAC trial (n = 10 patients) and for patients with high TGFβ-15-specific T cell response in the TRIPLE-R trial (n = 9 patients). For (A and D), data presented in a box-and-whisker plot, with dots representing individual patients and *$p<0.05$ according to unpaired two-tailed T test.

Supplementary table 1. Differentially expressed genes in Pan02 tumors from mice treated with an anti-IL6R antibody compared to Pan02 tumors from untreated mice (cut off p-adjusted value <0.05 and absolute. log2 fold change >0.585)

UPREGULATED			
**gene**	**log2FoldChange**	**pvalue**	**padj**
Ighv5-9-1	6,09	3,93E-09	2,35E-06
Igkv3-12	5,89	1,91E-05	2,12E-03
Ighv2-5	5,50	2,75E-05	2,73E-03
Igkv6-17	4,80	6,91E-05	5,51E-03
Cyp11b1	4,49	1,70E-04	1,00E-02
Igkv9-120	4,13	2,48E-07	7,50E-05
Gsta2	4,11	2,31E-04	1,23E-02
Gzmf	4,04	5,42E-12	1,20E-08
Il13	3,82	7,74E-06	1,04E-03
Retnla	3,70	3,70E-06	5,70E-04
Duoxa2	3,63	1,08E-03	3,48E-02
Ighv11-2	3,36	9,80E-05	6,99E-03
Igkv19-93	3,31	1,36E-07	4,45E-05
Car4	3,14	6,78E-06	9,60E-04
Susd4	2,94	6,34E-05	5,35E-03
Igkv14-126	2,94	5,90E-04	2,34E-02
Igkj4	2,85	9,71E-05	6,97E-03
Igkv8-19	2,81	7,67E-04	2,73E-02
Msx3	2,79	1,07E-04	7,32E-03
Ccl24	2,76	2,32E-08	1,06E-05
Gm10354	2,70	2,52E-06	4,26E-04
Iglc2	2,63	7,21E-06	9,94E-04
Ighv6-6	2,56	9,21E-06	1,19E-03
Gm9758	2,52	6,31E-05	5,35E-03
Alox15	2,51	1,35E-06	2,82E-04
Mrgprg	2,36	4,64E-04	2,00E-02
Mucl1	2,26	6,10E-12	1,20E-08
Ighm	2,23	2,81E-06	4,70E-04
Iglv3	2,21	1,44E-04	9,00E-03
Ighv2-2	2,20	4,84E-04	2,08E-02
Igkv4-86	2,12	6,34E-04	2,44E-02
Alox12e	2,12	1,42E-04	8,95E-03
Jchain	2,11	2,12E-05	2,28E-03
Chst13	2,11	2,63E-04	1,33E-02
Cebpe	2,03	3,80E-07	1,03E-04
Aldh1a2	2,02	1,14E-08	6,19E-06
Pla2g2e	1,99	1,02E-04	7,07E-03
Tmem54	1,99	9,36E-04	3,14E-02
Igkc	1,97	6,52E-06	9,40E-04
Prg2	1,91	4,07E-04	1,83E-02
Il13ra2	1,90	5,37E-05	4,71E-03
Tdo2	1,90	1,21E-03	3,76E-02
Retnlg	1,85	7,54E-08	2,69E-05
Cyp2e1	1,83	1,48E-06	2,97E-04
Igkj2	1,83	3,14E-05	3,04E-03
Ighv1-55	1,82	9,68E-05	6,97E-03
Iglc1	1,76	1,45E-06	2,96E-04
Gm49087	1,76	9,57E-04	3,19E-02
Pla2g2d	1,74	1,86E-09	1,44E-06
Mrgprb2	1,74	8,72E-07	1,96E-04
Gata1	1,72	1,15E-03	3,62E-02
Ear2	1,72	2,07E-09	1,44E-06
Chodl	1,72	1,25E-03	3,81E-02
Mrgprb1	1,67	2,61E-07	7,60E-05
Tpsb2	1,67	2,60E-10	2,72E-07
Lrat	1,66	1,00E-04	7,03E-03
Hc	1,66	3,75E-12	1,18E-08
Ighj1	1,65	5,98E-05	5,13E-03
Rprm	1,64	2,92E-07	8,20E-05
Gm21083	1,63	5,19E-04	2,15E-02
Adora3	1,62	2,36E-08	1,06E-05
Tpsg1	1,61	1,20E-04	8,00E-03
Arg1	1,60	1,99E-17	3,13E-13
Cpa3	1,54	4,32E-09	2,42E-06
Fcer1a	1,53	1,92E-08	9,36E-06
Ly6d	1,52	3,41E-05	3,22E-03
Cma1	1,52	5,69E-08	2,13E-05
Ccl17	1,49	2,15E-10	2,41E-07
A130071D04Rik	1,47	2,66E-04	1,34E-02
Cd209e	1,47	5,61E-06	8,23E-04
Chil5	1,43	1,64E-03	4,64E-02
Mcpt4	1,42	4,80E-07	1,26E-04
Igkv12-46	1,42	9,00E-04	3,08E-02
Cd200r3	1,40	3,76E-05	3,44E-03
Ramp3	1,37	1,97E-08	9,36E-06
Tmem158	1,36	3,23E-10	3,17E-07
Nphs2	1,35	2,12E-04	1,16E-02

DOWNREGULATED			
**gene**	**log2FoldChange**	**pvalue**	**padj**
Atp7a	-0,59	3,18E-04	1,51E-02
Cand2	-0,59	1,48E-03	4,30E-02
Tln2	-0,59	1,53E-06	3,03E-04
Sorbs1	-0,60	1,25E-03	3,81E-02
Slc12a2	-0,60	1,80E-03	4,87E-02
Mast4	-0,60	2,16E-05	2,31E-03
Usp9x	-0,60	4,89E-06	7,31E-04
Faxc	-0,60	6,07E-04	2,38E-02
Rprd1a	-0,60	1,34E-05	1,61E-03
Col8a1	-0,61	6,08E-04	2,38E-02
Zfp334	-0,61	2,77E-04	1,36E-02
Syt7	-0,61	2,76E-04	1,36E-02
Amotl1	-0,62	1,12E-04	7,60E-03
Adamtsl1	-0,62	5,34E-05	4,71E-03
Akap12	-0,62	2,43E-06	4,22E-04
Cdh5	-0,62	9,35E-05	6,87E-03
Dlc1	-0,63	1,42E-04	8,95E-03
Zbtb37	-0,63	8,79E-05	6,64E-03
Lrrk2	-0,64	7,70E-06	1,04E-03
Nectin3	-0,64	5,17E-04	2,15E-02
Uprt	-0,64	9,47E-04	3,17E-02
Dysf	-0,64	1,66E-07	5,13E-05
N4bp2	-0,64	1,09E-05	1,36E-03
Filip1	-0,64	1,47E-03	4,28E-02
Robo4	-0,64	1,23E-04	8,13E-03
Pcdh12	-0,64	7,83E-04	2,78E-02
Plekhg1	-0,65	2,39E-05	2,47E-03
Syne1	-0,65	4,04E-09	2,35E-06
Pde10a	-0,65	3,48E-04	1,63E-02
Sned1	-0,65	6,00E-04	2,37E-02
Vash1	-0,65	9,96E-05	7,01E-03
Zfp521	-0,66	4,57E-04	1,99E-02
Fbn1	-0,66	2,38E-12	9,35E-09
Lcor	-0,66	5,16E-04	2,15E-02
Ecrg4	-0,67	1,51E-03	4,34E-02
Plekhh2	-0,67	1,05E-03	3,41E-02
Tmem47	-0,68	1,96E-06	3,67E-04
Prrg3	-0,68	1,65E-03	4,65E-02
Adamts5	-0,68	8,44E-04	2,90E-02
Pgap1	-0,68	6,39E-04	2,45E-02
Pdpr	-0,69	6,68E-05	5,50E-03
Atp8a1	-0,70	7,55E-06	1,03E-03
She	-0,70	1,77E-04	1,03E-02
Ddx60	-0,70	1,32E-04	8,44E-03
Tenm3	-0,70	6,66E-05	5,50E-03
Peg3	-0,70	1,30E-04	8,40E-03
Rgs5	-0,70	1,99E-04	1,11E-02
Slc2a13	-0,71	3,56E-05	3,29E-03
Rasal2	-0,71	3,18E-06	5,15E-04
Arhgap28	-0,71	3,21E-04	1,52E-02
Dst	-0,71	1,61E-10	2,10E-07
Fam171b	-0,71	6,51E-04	2,48E-02
Map3k20	-0,71	1,17E-06	2,52E-04
Pde3a	-0,72	7,12E-06	9,90E-04
Alcam	-0,73	2,09E-04	1,15E-02
Pde4dip	-0,73	1,05E-03	3,41E-02
Garem1	-0,73	3,10E-04	1,49E-02
Lgr4	-0,74	6,03E-06	8,77E-04
Prickle2	-0,74	3,06E-06	5,01E-04
Adgra3	-0,75	1,79E-05	2,03E-03
Mmrn2	-0,75	1,11E-04	7,54E-03
Flrt2	-0,75	2,26E-06	4,03E-04
Vgll3	-0,75	3,11E-05	3,03E-03
Cspg4	-0,76	1,02E-05	1,30E-03
Cyyr1	-0,77	4,98E-04	2,12E-02
Nav3	-0,77	3,65E-08	1,49E-05
Osbpl6	-0,77	1,25E-03	3,81E-02
Heyl	-0,77	8,25E-04	2,88E-02
Shroom4	-0,77	2,31E-04	1,23E-02
Lama2	-0,77	1,44E-07	4,62E-05
Timp3	-0,77	7,86E-04	2,78E-02
Ecm2	-0,78	1,19E-03	3,70E-02
Cd93	-0,78	1,43E-04	8,96E-03
Atp8b1	-0,78	1,09E-05	1,36E-03
Arhgap32	-0,79	1,51E-07	4,74E-05
Sacs	-0,79	2,47E-06	4,22E-04
Mef2c	-0,79	3,78E-04	1,75E-02

Mrgprx2	1,31	6,85E-05	5,50E-03
Tph1	1,31	8,51E-06	1,12E-03
Mgl2	1,29	1,04E-12	8,02E-09
Pdcd1lg2	1,29	3,93E-10	3,63E-07
Tpsab1	1,28	3,69E-06	5,70E-04
Edn2	1,25	1,68E-11	2,94E-08
Gm6093	1,22	6,69E-05	5,50E-03
Cck	1,21	2,49E-04	1,28E-02
Gm41724	1,20	7,08E-04	2,61E-02
Siglecf	1,18	1,49E-03	4,31E-02
Cish	1,17	2,87E-07	8,20E-05
Aif1l	1,16	2,82E-11	4,42E-08
Slc7a2	1,16	2,11E-09	1,44E-06
Plet1	1,15	1,55E-05	1,81E-03
Asgr2	1,14	1,11E-05	1,37E-03
P2rx1	1,13	1,25E-03	3,81E-02
Ms4a2	1,10	2,94E-05	2,89E-03
F7	1,08	5,14E-07	1,30E-04
Il1rl1	1,08	6,72E-07	1,60E-04
Diras2	1,08	1,70E-04	1,00E-02
Slpi	1,07	7,49E-05	5,82E-03
Nrn1	1,06	6,97E-04	2,60E-02
Foxd1	1,06	7,66E-04	2,73E-02
Ccl22	1,06	3,09E-08	1,31E-05
Cyp11a1	1,05	6,02E-04	2,37E-02
Olr1	1,04	7,09E-05	5,60E-03
Hsd11b1	1,01	7,63E-07	1,76E-04
H2-M2	1,00	2,40E-04	1,26E-02
Ccl6	0,99	2,47E-06	4,22E-04
Gata2	0,99	4,72E-06	7,13E-04
Tarm1	0,98	2,56E-07	7,60E-05
Prr15	0,97	2,30E-05	2,44E-03
Atp8b5	0,97	9,90E-04	3,27E-02
Ccr3	0,97	1,43E-03	4,16E-02
F10	0,96	3,69E-08	1,49E-05
Alox5	0,96	7,30E-07	1,71E-04
Stac2	0,94	1,42E-06	2,94E-04
Nfe2	0,94	1,41E-03	4,13E-02
Insc	0,94	1,25E-04	8,18E-03
Lyz1	0,92	8,92E-05	6,70E-03
Tnfrsf9	0,91	3,87E-08	1,49E-05
Ltc4s	0,91	9,89E-05	7,00E-03
Aqp9	0,87	6,23E-04	2,41E-02
Enpp3	0,87	9,57E-05	6,96E-03
Penk	0,86	1,73E-05	1,98E-03
F5	0,85	1,36E-03	4,05E-02
Gata3	0,83	3,58E-05	3,29E-03
Atp6v0d2	0,81	4,03E-04	1,82E-02
Gzmb	0,80	1,59E-04	9,61E-03
Ptgds	0,80	2,70E-04	1,35E-02
Dok2	0,78	2,58E-05	2,65E-03
Lgals3	0,77	1,07E-07	3,66E-05
Trf	0,75	7,61E-10	6,64E-07
Srgn	0,75	3,07E-09	1,93E-06
Ccl8	0,74	8,19E-05	6,24E-03
Ms4a4a	0,74	2,62E-05	2,67E-03
Hemk1	0,73	2,77E-04	1,36E-02
Enpp2	0,72	1,34E-06	2,82E-04
Mgarp	0,70	1,39E-03	4,09E-02
Wasf1	0,70	1,75E-03	4,78E-02
Fgfbp3	0,70	8,36E-04	2,90E-02
Ccl9	0,69	2,23E-04	1,20E-02
Hspa1b	0,67	3,84E-04	1,77E-02
Pf4	0,65	1,62E-04	9,67E-03
Pmm1	0,65	1,19E-05	1,45E-03
Wnt6	0,65	1,70E-03	4,73E-02
Bcl2a1b	0,64	5,32E-06	7,88E-04
Ptgs1	0,64	3,33E-06	5,28E-04
Gm9843	0,64	1,73E-04	1,01E-02
Fetub	0,64	1,83E-03	4,92E-02
Itga2b	0,64	3,26E-05	3,10E-03
Map4k1	0,63	7,71E-05	5,97E-03
Aldoc	0,63	3,09E-04	1,49E-02
Kit	0,62	7,33E-04	2,67E-02
Cadm4	0,62	1,77E-03	4,82E-02
Masp1	0,62	1,37E-04	8,76E-03
Bcl2a1a	0,61	1,80E-04	1,04E-02
Rubcnl	0,61	1,07E-04	7,32E-03
Syt12	0,60	1,10E-03	3,52E-02
Azin2	0,60	5,16E-05	4,61E-03
Pla1a	0,60	5,42E-05	4,73E-03
Acadm	0,60	9,90E-05	7,00E-03
Tigit	0,59	2,33E-04	1,23E-02
Cpe	0,59	1,07E-04	7,32E-03

Pcdhgc5	-0,79	7,13E-04	2,61E-02
Dcbld1	-0,79	7,58E-04	2,73E-02
Emcn	-0,80	4,61E-04	2,00E-02
Adgrl4	-0,80	1,10E-04	7,49E-03
Slit2	-0,80	3,47E-05	3,23E-03
Bnc2	-0,81	9,33E-06	1,19E-03
Itgbl1	-0,81	6,20E-04	2,40E-02
Shank3	-0,81	7,96E-07	1,81E-04
Pcdhgb6	-0,82	2,72E-05	2,73E-03
Zbed6	-0,82	5,70E-07	1,40E-04
Gm6377	-0,83	5,48E-04	2,24E-02
Syt15	-0,83	1,19E-03	3,70E-02
Htr2a	-0,83	6,49E-04	2,48E-02
Col14a1	-0,84	2,32E-04	1,23E-02
Hdgfl3	-0,84	2,03E-06	3,70E-04
Lamc2	-0,85	6,62E-04	2,50E-02
Plce1	-0,85	9,81E-08	3,43E-05
Kcnj2	-0,85	1,27E-04	8,28E-03
Serpine1	-0,85	1,28E-04	8,32E-03
Kif21a	-0,85	3,91E-04	1,79E-02
Angpt1	-0,86	4,16E-06	6,34E-04
Cacna2d1	-0,86	9,29E-05	6,87E-03
Areg	-0,86	5,63E-04	2,26E-02
Lama3	-0,87	1,70E-03	4,72E-02
Zfhx4	-0,88	1,75E-08	9,14E-06
Piezo2	-0,88	8,38E-04	2,90E-02
Rapgef5	-0,89	2,34E-05	2,45E-03
Btc	-0,89	6,92E-04	2,59E-02
Slco2a1	-0,90	2,83E-05	2,79E-03
Kif26a	-0,90	4,29E-04	1,91E-02
Tmtc1	-0,91	5,32E-07	1,33E-04
Pcdhb16	-0,92	4,70E-04	2,02E-02
Sgcd	-0,94	7,59E-04	2,73E-02
Kdr	-0,95	3,30E-06	5,28E-04
Mpdz	-0,96	2,11E-09	1,44E-06
Ret	-0,97	8,91E-06	1,17E-03
Tmem200a	-0,99	7,26E-05	5,70E-03
Zfp791	-0,99	9,51E-04	3,18E-02
Ildr2	-1,00	1,66E-06	3,25E-04
Vcan	-1,00	1,11E-07	3,72E-05
Tlcd5	-1,00	1,09E-03	3,52E-02
Kcnk3	-1,02	5,70E-04	2,28E-02
Sema6a	-1,03	3,88E-08	1,49E-05
Sbsn	-1,04	1,81E-05	2,03E-03
Olfml2a	-1,05	1,93E-06	3,66E-04
Zfp804a	-1,05	1,02E-03	3,33E-02
Nebl	-1,05	2,10E-06	3,79E-04
Atp9a	-1,05	6,83E-05	5,50E-03
Hpgd	-1,07	1,76E-04	1,02E-02
Cdh15	-1,07	1,85E-03	4,95E-02
Slc16a9	-1,09	2,50E-04	1,28E-02
Cemip	-1,10	1,94E-08	9,36E-06
Plekhh1	-1,10	8,11E-04	2,84E-02
Abcc9	-1,12	7,51E-08	2,69E-05
Ptprb	-1,14	5,87E-12	1,20E-08
Prex2	-1,17	3,23E-07	8,90E-05
Gm37233	-1,18	5,78E-04	2,31E-02
Slc4a4	-1,18	4,51E-04	1,99E-02
Cnn1	-1,18	7,37E-05	5,76E-03
Kcna5	-1,19	1,21E-03	3,75E-02
Adh1	-1,21	2,16E-04	1,17E-02
Parm1	-1,22	1,53E-12	8,02E-09
Klf8	-1,23	9,72E-05	6,97E-03
Pcdh17	-1,24	1,92E-04	1,09E-02
Adamts3	-1,25	5,40E-04	2,22E-02
Wnt16	-1,26	3,07E-04	1,49E-02
Klf12	-1,26	7,87E-04	2,78E-02
Foxp2	-1,27	5,51E-04	2,24E-02
Nox4	-1,28	1,60E-10	2,10E-07
Actg2	-1,31	1,19E-09	9,87E-07
Tmem30b	-1,34	7,25E-04	2,65E-02
Scml4	-1,34	6,87E-05	5,50E-03
Sox11	-1,47	5,12E-04	2,15E-02
Ces2g	-1,51	1,53E-05	1,79E-03
Megf10	-1,53	2,11E-05	2,28E-03
Sdk2	-1,55	1,46E-04	9,08E-03
Robo1	-1,57	1,93E-10	2,33E-07
Cxcl15	-1,61	2,32E-05	2,45E-03
Itga2	-1,67	3,44E-05	3,22E-03
Col4a5	-1,69	2,23E-04	1,20E-02
Lratd1	-1,88	1,99E-06	3,69E-04
Kcnh1	-1,89	1,01E-04	7,05E-03
Enpp6	-2,04	6,66E-06	9,51E-04
Kcnk1	-2,08	7,07E-06	9,90E-04

Supplementary table 2. Differentially expressed genes in Pan02 tumors from mice treated with the TGFβ vaccine and an anti-IL6R antibody compared to Pan02 tumors from mice that exclusively received the TGFβ vaccine (cut off p-adjusted value <0.05 and absolute log2 fold change >0.585).

UPREGULATED			
gene	log2FoldChange	pvalue	padj
Ighv1-4	9,20	4,37E-06	3,83E-04
Ighv14-4	8,14	3,40E-07	4,22E-05
Ighv2-6-8	5,88	4,42E-06	3,85E-04
Msx3	5,21	7,29E-14	3,55E-11
Igkv8-28	5,02	5,32E-08	7,96E-06
Ighg3	4,48	3,92E-05	2,52E-03
Igkv16-104	4,40	4,05E-05	2,60E-03
Ighv6-4	4,38	7,36E-04	2,45E-02
Ighv2-9	4,37	3,86E-04	1,49E-02
Ighv1-22	4,26	5,85E-04	2,04E-02
Ighv1-36	4,11	4,87E-08	7,42E-06
Ighv5-6	4,10	2,84E-04	1,16E-02
Ighv4-1	4,07	3,71E-07	4,55E-05
Ighv1-11	4,03	2,03E-04	9,03E-03
Ighv9-1	3,75	2,57E-05	1,82E-03
Igkv7-33	3,53	6,69E-04	2,26E-02
Ighv3-3	3,12	1,34E-03	3,89E-02
Igkv14-100	2,91	8,35E-04	2,71E-02
Rnase2a	2,91	2,13E-04	9,36E-03
Ighv7-1	2,90	1,60E-04	7,57E-03
Igkv8-16	2,82	1,74E-03	4,78E-02
Iglj1	2,77	1,82E-05	1,36E-03
Igkv15-103	2,70	1,08E-04	5,58E-03
Igkv5-48	2,58	2,54E-05	1,82E-03
Ighv6-6	2,49	4,88E-04	1,76E-02
Ighv3-6	2,42	1,98E-06	2,01E-04
Iglv1	2,40	3,73E-05	2,42E-03
Ighj3	2,37	2,23E-04	9,70E-03
Iglc1	2,36	1,95E-04	8,76E-03
Igkv1-135	2,32	4,38E-10	1,16E-07
Gm31378	2,31	1,12E-04	5,71E-03
Mzb1	2,30	3,49E-08	5,56E-06
Igkv8-24	2,28	1,67E-08	2,90E-06
Ighv1-19	2,23	4,09E-07	4,98E-05
Siah3	2,14	6,56E-05	3,78E-03
Ighv7-3	2,11	1,64E-10	4,63E-08
Hoxc11	2,09	1,18E-03	3,60E-02
Ighj1	2,04	2,76E-11	9,32E-09
Gm17907	1,96	1,17E-03	3,58E-02
Ighj2	1,92	2,30E-04	9,93E-03
Igkv14-126	1,87	1,12E-05	9,00E-04
Jchain	1,87	6,61E-07	7,67E-05
Retnla	1,87	2,22E-36	3,89E-32
Serpinb2	1,69	2,06E-06	2,05E-04
Igkv4-68	1,69	1,13E-03	3,50E-02
Ighv11-2	1,68	1,72E-04	7,97E-03
Igkj2	1,65	5,74E-07	6,79E-05
Ly6c2	1,65	1,86E-14	1,02E-11
Ighm	1,64	9,53E-19	8,35E-16
Golga7b	1,62	1,48E-04	7,04E-03
Alox12e	1,59	1,08E-03	3,39E-02
Grhl3	1,48	5,46E-05	3,29E-03
Pou2af1	1,47	7,43E-07	8,45E-05
Igkv5-43	1,42	4,14E-06	3,66E-04
Tmem229a	1,37	3,10E-05	2,11E-03
Mrgprx2	1,32	2,47E-05	1,78E-03
Adgrg7	1,28	5,95E-05	3,53E-03
Rbp2	1,21	1,96E-05	1,44E-03
Papln	1,20	3,57E-04	1,41E-02
Entpd3	1,19	1,46E-07	2,03E-05
Cd209d	1,13	7,27E-09	1,40E-06
Tnfsf15	1,13	3,57E-06	3,21E-04
Igkc	1,12	3,68E-04	1,45E-02
Rps29	1,11	3,78E-09	7,79E-07
Derl3	1,10	4,30E-05	2,71E-03
Rpl24	1,10	3,40E-06	3,11E-04
Krt79	1,10	5,25E-05	3,19E-03
Itga10	1,09	2,97E-05	2,06E-03
Nfe2	1,08	5,87E-04	2,04E-02
Lilra6	1,07	1,37E-04	6,69E-03
Rpl3-ps2	1,07	1,23E-03	3,69E-02
Folr2	1,06	7,17E-10	1,75E-07
Mmp27	1,03	1,33E-05	1,05E-03
Ccl6	1,03	3,50E-14	1,80E-11
Ly6d	1,02	2,33E-04	9,97E-03
Wfdc17	1,02	1,24E-08	2,27E-06
Cebpe	1,00	3,65E-04	1,44E-02
Car8	0,99	1,99E-05	1,46E-03
Cxcl3	0,98	4,11E-04	1,55E-02
Ccl9	0,96	6,89E-17	5,03E-14
Cma1	0,93	1,35E-09	3,15E-07
Cd244a	0,91	3,39E-08	5,46E-06
Gm830	0,89	1,27E-03	3,76E-02

DOWNREGULATED			
gene	log2FoldChange	pvalue	padj
Usp2	-0,59	1,71E-04	7,96E-03
Gm49990	-0,59	1,14E-03	3,51E-02
Shisa4	-0,59	2,10E-06	2,08E-04
Hoxc8	-0,59	3,39E-05	2,25E-03
Igfbp5	-0,59	6,88E-05	3,90E-03
Ehd3	-0,59	2,80E-05	1,98E-03
Nr1d1	-0,60	1,33E-06	1,40E-04
Golga4	-0,60	1,51E-08	2,71E-06
Stau2	-0,60	1,81E-04	8,26E-03
Nav2	-0,60	1,22E-05	9,69E-04
Sncg	-0,61	2,46E-04	1,04E-02
Auts2	-0,61	5,74E-05	3,43E-03
Zfp106	-0,61	2,79E-07	3,59E-05
Dysf	-0,61	9,99E-05	5,26E-03
Lrrc47	-0,62	3,38E-06	3,10E-04
Klra2	-0,62	4,41E-04	1,63E-02
1700025G04Rik	-0,62	8,12E-05	4,44E-03
Hs6st2	-0,62	1,27E-04	6,35E-03
Prkcq	-0,62	8,84E-04	2,84E-02
Cib2	-0,64	8,70E-04	2,81E-02
Klhl30	-0,64	2,91E-06	2,71E-04
Fyco1	-0,64	3,11E-07	3,89E-05
Eln	-0,65	4,55E-05	2,84E-03
Ppp1r12b	-0,65	8,42E-07	9,46E-05
Ak1	-0,66	1,28E-07	1,80E-05
Cacna2d1	-0,66	1,64E-05	1,26E-03
Ptp4a3	-0,67	6,03E-08	8,96E-06
Sertad4	-0,67	6,33E-04	2,17E-02
Mreg	-0,67	2,77E-04	1,14E-02
Amotl1	-0,67	2,30E-08	3,81E-06
Abcc9	-0,68	8,15E-05	4,45E-03
Itgbl1	-0,68	8,56E-06	7,04E-04
Chchd10	-0,68	6,56E-05	3,78E-03
Igf2	-0,69	3,65E-05	2,38E-03
Map1a	-0,69	3,35E-06	3,09E-04
Apold1	-0,69	9,92E-04	3,13E-02
Nefh	-0,71	1,03E-03	3,24E-02
Plpp7	-0,71	7,00E-04	2,35E-02
Prokr2	-0,73	5,67E-04	1,99E-02
Gm4841	-0,76	2,57E-05	1,82E-03
Ch25h	-0,77	3,98E-04	1,52E-02
Slc9a2	-0,77	1,09E-03	3,41E-02
Eda2r	-0,78	1,95E-05	1,44E-03
BC023105	-0,78	1,47E-03	4,22E-02
Gbp10	-0,78	1,27E-03	3,76E-02
Adssl1	-0,79	2,50E-11	8,77E-09
Eif3j2	-0,79	1,54E-03	4,36E-02
Gm9800	-0,80	1,66E-03	4,61E-02
Art3	-0,80	1,05E-04	5,44E-03
Gmpr	-0,81	1,85E-04	8,40E-03
Sema6a	-0,81	1,64E-05	1,26E-03
Sorbs1	-0,82	1,15E-06	1,25E-04
Gm12185	-0,82	1,20E-04	6,07E-03
Tbx3	-0,83	5,23E-04	1,87E-02
Ldhb	-0,83	2,33E-04	9,97E-03
Klhl29	-0,85	3,26E-04	1,31E-02
St6galnac5	-0,85	1,36E-04	6,68E-03
Acsl1	-0,85	2,02E-09	4,43E-07
Paqr5	-0,85	1,18E-03	3,59E-02
Pde11a	-0,85	3,35E-04	1,34E-02
Slit2	-0,86	6,91E-11	2,09E-08
Gpd1	-0,86	1,64E-06	1,70E-04
Ret	-0,86	8,55E-05	4,64E-03
Lrrn2	-0,87	2,66E-04	1,11E-02
Lepr	-0,87	3,48E-06	3,16E-04
Prg4	-0,87	1,49E-05	1,16E-03
Phtf2	-0,87	4,17E-08	6,47E-06
Dgat2	-0,88	1,02E-04	5,35E-03
Tpm1	-0,88	8,37E-09	1,58E-06
Sgcd	-0,88	4,84E-04	1,75E-02
Plekhb1	-0,89	6,51E-06	5,59E-04
Tmem38b	-0,90	5,96E-07	7,00E-05
Kcnb1	-0,90	1,17E-04	5,90E-03
Cx3cr1	-0,91	4,97E-08	7,51E-06
Itgb6	-0,91	4,73E-05	2,92E-03
Timp3	-0,91	1,09E-08	2,02E-06
Extl1	-0,92	2,02E-04	9,00E-03
Prkg1	-0,94	1,26E-09	2,98E-07
Neurl1a	-0,95	3,09E-05	2,11E-03
Gm43305	-0,95	7,27E-05	4,07E-03
Fgf13	-0,95	1,31E-03	3,82E-02
Meox1	-0,95	1,59E-08	2,79E-06
Tenm3	-0,96	4,29E-08	6,59E-06

Bdh2	0,89	1,63E-04	7,69E-03
Cxcl5	0,88	4,62E-07	5,53E-05
Hmox1	0,86	2,33E-10	6,28E-08
Rab44	0,86	9,53E-04	3,03E-02
Mrgprb1	0,84	7,47E-06	6,27E-04
Igkj1	0,84	9,56E-04	3,03E-02
Hdc	0,84	1,74E-04	8,03E-03
Stac2	0,84	8,46E-05	4,60E-03
Efhd1	0,83	2,81E-05	1,98E-03
Slfn1	0,83	2,60E-06	2,48E-04
Rnf128	0,79	1,76E-03	4,80E-02
Ighj4	0,78	7,79E-04	2,56E-02
Mcoln2	0,77	1,09E-06	1,20E-04
Krt76	0,77	3,70E-04	1,45E-02
Chga	0,76	4,38E-04	1,63E-02
Gt(ROSA)26Sor	0,75	8,33E-04	2,71E-02
Oas1g	0,74	4,66E-04	1,70E-02
Akr1c18	0,74	4,84E-04	1,75E-02
Tpsb2	0,72	6,47E-08	9,44E-06
Fat2	0,72	1,28E-04	6,36E-03
Ang	0,72	1,09E-04	5,58E-03
Cpa3	0,72	1,85E-07	2,50E-05
Vegfd	0,71	5,65E-04	1,99E-02
Rpl35a	0,71	6,73E-07	7,76E-05
Rps13	0,71	3,51E-07	4,33E-05
Shld1	0,70	1,26E-03	3,75E-02
Egln3	0,68	1,73E-07	2,35E-05
Oasl1	0,68	7,99E-06	6,60E-04
Ccl8	0,67	2,41E-07	3,18E-05
Clec3b	0,66	7,45E-06	6,27E-04
Gper1	0,65	2,68E-04	1,11E-02
Spon2	0,64	7,17E-06	6,07E-04
Hpse	0,63	8,22E-07	9,29E-05
Ero1a	0,63	9,91E-06	8,04E-04
Mcpt4	0,63	6,33E-05	3,72E-03
Bnip3	0,62	1,04E-06	1,16E-04
Gm7536	0,62	2,92E-04	1,19E-02
Tmem45a	0,62	1,66E-06	1,71E-04
Cd209e	0,62	1,17E-03	3,58E-02
Snhg8	0,61	5,00E-05	3,06E-03
Ddx60	0,61	2,40E-04	1,02E-02
Pf4	0,60	1,33E-06	1,40E-04
Ifi204	0,60	1,29E-05	1,02E-03
Fcer1a	0,60	3,51E-04	1,39E-02
Mif	0,59	4,64E-07	5,53E-05
Oas2	0,59	5,27E-04	1,88E-02
Clec10a	0,59	2,17E-04	9,47E-03
Npl	0,59	4,18E-04	1,57E-02
Selenbp1	0,59	7,37E-05	4,10E-03
Cox7c	0,59	1,11E-05	8,93E-04
Siglec1	0,59	1,71E-04	7,96E-03

Cdnf	-0,96	6,85E-05	3,90E-03
Scn2a	-0,96	6,00E-04	2,08E-02
Mn1	-0,96	2,15E-06	2,09E-04
Map3k20	-0,97	3,86E-09	7,87E-07
Casz1	-0,97	6,15E-06	5,31E-04
Sele	-0,97	2,33E-04	9,97E-03
Megf11	-0,97	1,43E-04	6,88E-03
Tmtc1	-0,98	3,06E-07	3,86E-05
Kcnj10	-0,99	1,81E-03	4,93E-02
Nol3	-0,99	1,12E-06	1,23E-04
Phka1	-1,01	1,56E-07	2,16E-05
Piezo2	-1,04	2,35E-08	3,85E-06
Filip1	-1,05	1,37E-08	2,47E-06
Atp9a	-1,07	2,59E-09	5,60E-07
Sh3rf3	-1,08	3,09E-05	2,11E-03
Dmd	-1,08	5,81E-10	1,52E-07
Hspb8	-1,08	1,02E-07	1,43E-05
Fsd1l	-1,08	7,11E-05	4,00E-03
Fbxo32	-1,09	8,10E-16	5,25E-13
Flnc	-1,09	2,39E-04	1,02E-02
Amot	-1,10	1,73E-11	6,67E-09
Dnaja4	-1,11	2,08E-08	3,50E-06
Tacc2	-1,12	7,31E-13	3,12E-10
Cilp	-1,12	2,84E-11	9,39E-09
Gfra1	-1,13	6,86E-07	7,86E-05
Pgm5	-1,14	3,54E-05	2,32E-03
Jph2	-1,14	6,87E-09	1,34E-06
Cryab	-1,15	3,94E-04	1,51E-02
Zbtb16	-1,17	7,15E-06	6,07E-04
Kcnq5	-1,17	3,45E-05	2,28E-03
Clip4	-1,18	4,06E-09	8,18E-07
Grb14	-1,19	7,12E-05	4,00E-03
Ddit4l	-1,21	1,94E-10	5,30E-08
Rrad	-1,21	3,78E-08	5,97E-06
Mef2c	-1,21	3,68E-11	1,19E-08
Clu	-1,22	2,24E-18	1,79E-15
Pgap4	-1,23	5,82E-04	2,03E-02
Me3	-1,23	6,55E-05	3,78E-03
Epha3	-1,23	8,85E-08	1,27E-05
Cyp2f2	-1,25	1,97E-05	1,44E-03
Cand2	-1,29	1,91E-11	7,12E-09
Gvin-ps6	-1,30	1,40E-04	6,79E-03
F830016B08Rik	-1,30	6,19E-11	1,90E-08
Fbxl22	-1,30	4,38E-04	1,63E-02
Camk2a	-1,31	1,65E-09	3,75E-07
Retn	-1,31	8,59E-05	4,64E-03
Alpk2	-1,33	5,45E-05	3,29E-03
Gnao1	-1,34	2,11E-06	2,08E-04
Pde4dip	-1,35	5,94E-20	6,94E-17
Eno3	-1,37	6,35E-16	4,45E-13
Perm1	-1,37	6,55E-08	9,49E-06
Unc45b	-1,38	7,77E-11	2,31E-08
Rtn2	-1,40	7,12E-14	3,55E-11
Prob1	-1,42	7,81E-14	3,70E-11
Homer2	-1,42	2,05E-06	2,05E-04
Tcea3	-1,46	2,87E-12	1,20E-09
Mustn1	-1,48	2,93E-04	1,19E-02
Prr33	-1,51	3,40E-09	7,08E-07
Rorc	-1,53	2,40E-07	3,18E-05
H19	-1,54	6,36E-04	2,18E-02
Nexn	-1,54	3,87E-04	1,49E-02
Fgf23	-1,54	1,08E-04	5,57E-03
Gm37829	-1,54	1,33E-04	6,59E-03
Fndc5	-1,56	8,05E-09	1,53E-06
Tpbgl	-1,58	6,45E-08	9,44E-06
Reep1	-1,59	3,55E-06	3,20E-04
Cfd	-1,61	1,28E-04	6,37E-03
Ache	-1,62	1,45E-06	1,52E-04
Hspb1	-1,64	6,63E-04	2,24E-02
Tpm2	-1,67	2,85E-05	2,00E-03
Thbs4	-1,70	1,21E-03	3,66E-02
Slc38a4	-1,72	1,94E-09	4,29E-07
Lmcd1	-1,73	2,67E-04	1,11E-02
Fhl1	-1,74	3,75E-05	2,42E-03
Rbm20	-1,74	3,18E-05	2,15E-03
Speg	-1,77	8,96E-19	8,26E-16
Cnksr1	-1,77	2,77E-11	9,32E-09
Atp1b2	-1,80	6,53E-22	9,54E-19
Pfkm	-1,80	1,43E-26	6,26E-23
Tuba8	-1,81	7,02E-13	3,07E-10
Hmcn2	-1,81	4,60E-04	1,69E-02
Sync	-1,83	1,17E-10	3,35E-08
Limch1	-1,85	2,32E-25	8,12E-22
Tlcd4	-1,89	1,23E-06	1,32E-04
Mlf1	-1,89	6,62E-10	1,66E-07
Usp13	-1,89	2,14E-15	1,29E-12
Tmem38a	-1,91	6,31E-22	9,54E-19
Ppp1r1a	-1,92	1,98E-06	2,01E-04
Myh14	-1,93	3,22E-14	1,71E-11

Kcnh1	-1,94	8,82E-05	4,74E-03
Akap6	-1,94	6,57E-10	1,66E-07
Synpo2	-1,96	2,19E-08	3,66E-06
Sbk2	-1,96	2,01E-08	3,43E-06
Car3	-1,97	5,76E-05	3,43E-03
Wfdc1	-1,98	6,81E-09	1,34E-06
Tceal5	-1,98	8,90E-08	1,27E-05
Mapt	-2,02	1,75E-11	6,67E-09
Fabp3	-2,02	1,37E-09	3,15E-07
Coq8a	-2,03	2,13E-06	2,09E-04
Kcnj12	-2,04	2,02E-08	3,43E-06
Kbtbd13	-2,04	1,03E-04	5,37E-03
Rragd	-2,05	2,99E-20	4,03E-17
Pkia	-2,05	2,62E-23	5,74E-20
Ank1	-2,08	7,36E-16	4,96E-13
Egf	-2,11	3,08E-09	6,58E-07
Kcnma1	-2,13	7,96E-11	2,32E-08
Adipoq	-2,14	3,50E-12	1,42E-09
Togaram2	-2,24	4,16E-07	5,02E-05
Ppp1r14c	-2,25	2,02E-11	7,22E-09
Asb14	-2,25	3,01E-06	2,79E-04
Acacb	-2,26	2,26E-24	5,89E-21
Myom1	-2,28	4,27E-05	2,71E-03
Ndrg2	-2,30	1,65E-07	2,25E-05
Wnk2	-2,31	1,67E-09	3,75E-07
Srpk3	-2,37	2,51E-07	3,29E-05
Tshr	-2,40	1,64E-04	7,69E-03
Lrrc39	-2,41	1,25E-06	1,32E-04
Asb2	-2,42	2,03E-28	1,19E-24
Ppp1r3c	-2,45	3,12E-23	6,07E-20
Hspb6	-2,45	1,55E-08	2,74E-06
Bves	-2,48	2,73E-06	2,59E-04
Abcb4	-2,51	1,02E-14	5,77E-12
Plin5	-2,57	1,82E-04	8,28E-03
Card14	-2,58	4,48E-09	8,92E-07
Ablim2	-2,58	4,77E-15	2,79E-12
Sptb	-2,58	2,66E-07	3,45E-05
Popdc2	-2,59	5,65E-11	1,77E-08
Mlxipl	-2,59	3,20E-09	6,76E-07
Yipf7	-2,59	2,96E-07	3,75E-05
Wipf3	-2,60	2,93E-07	3,75E-05
Fsd2	-2,61	1,01E-18	8,41E-16
Synm	-2,64	2,38E-13	1,10E-10
Slc2a4	-2,65	4,06E-22	7,11E-19
Capn3	-2,65	1,05E-06	1,17E-04
Ky	-2,68	4,79E-20	5,99E-17
Uckl1os	-2,68	1,93E-06	1,98E-04
Ctxn3	-2,69	2,46E-06	2,36E-04
Gm20597	-2,71	7,05E-05	3,98E-03
Mrln	-2,78	8,86E-05	4,74E-03
Pgam2	-2,79	2,47E-06	2,36E-04
Slc25a34	-2,83	2,89E-06	2,71E-04
Clcn1	-2,96	7,12E-10	1,75E-07
Sox6	-2,99	1,54E-19	1,58E-16
Slc8a3	-3,04	6,51E-12	2,59E-09
Mlip	-3,13	4,40E-17	3,35E-14
Kcnc1	-3,16	3,08E-13	1,38E-10
Zfp641	-3,18	5,65E-11	1,77E-08
Gm29773	-3,18	1,77E-05	1,34E-03
Stac3	-3,20	2,35E-24	5,89E-21
Scn4b	-3,23	6,77E-36	5,93E-32
Sec14l5	-3,23	3,95E-08	6,18E-06
Tmem233	-3,28	8,08E-20	8,84E-17
Phkg1	-3,31	2,90E-19	2,82E-16
Lrrc30	-3,47	8,18E-10	1,96E-07
Gm8424	-3,53	5,48E-05	3,29E-03
Nctc1	-3,58	6,43E-10	1,66E-07
Pla2g4e	-3,69	5,48E-05	3,29E-03
Dhrs7c	-3,70	9,76E-16	6,11E-13
Asb15	-4,19	1,87E-10	5,19E-08
Tigd4	-4,19	1,90E-04	8,60E-03
Esrrg	-4,39	1,95E-11	7,13E-09
St8sia5	-4,85	2,38E-08	3,86E-06
Casr	-6,89	1,08E-08	2,01E-06

Supplementary table 3. List of the 28 genes that were identified as differentially upregulated genes in both comparisons, in Pan02 tumors from mice treated with an anti-IL6R antibody compared to Pan02 tumors from untreated mice (Supplementary table 1) and in Pan02 tumors from mice treated with the TGFβ vaccine and an anti-IL6R antibody compared to Pan02 tumors from mice that exclusively received the TGFβ vaccine (Supplementary table 2). Cut off p-adjusted value <0.05 and absolute log2 fold change >0.585.

gene
Alox12e
Ccl6
Ccl8
Ccl9
Cd209e
Cebpe
Cma1
Cpa3
Fcer1a
Ighj1
Ighm
Ighv11-2
Ighv6-6
Igkc
Igkj2
Igkv14-126
Iglc1
Jchain
Ly6d
Mcpt4
Mrgprb1
Mrgprx2
Msx3
Nfe2
Pf4
Retnla
Stac2
Tpsb2

Supplementary table 4. Gene Ontology (GO) enrichment analysis for biological processes associated with the 28 common differentially upregulated genes listed in Supplementary table 3.

ID	Description	Immune related	Category	Count	%	p.adjust	pvalue	geneID
GO:0008037	humoral immune response	Yes	B cell function and immunoglobulins	7	6,51	1,0E-05	2,9E-08	Jchain/Pf4/Igkc/Ccl8/Ighm/Ighv11-2/Iglc1
GO:0001991	immunoglobulin mediated immune response	Yes	B cell function and immunoglobulins	5	4,65	2,5E-04	5,9E-06	Fcer1a/Igkc/Ighm/Ighv11-2/Iglc1
GO:0006959	B cell mediated immunity	Yes	B cell function and immunoglobulins	5	4,65	2,5E-04	6,4E-06	Fcer1a/Igkc/Ighm/Ighv11-2/Iglc1
GO:0050851	humoral immune response mediated by circulating immunoglobulin	Yes	B cell function and immunoglobulins	4	3,72	3,0E-04	8,6E-06	Igkc/Ighm/Ighv11-2/Iglc1
GO:0070098	B cell receptor signaling pathway	Yes	B cell function and immunoglobulins	4	3,72	3,0E-04	1,1E-05	Igkc/Ighm/Ighv11-2/Iglc1
GO:1990868	positive regulation of B cell activation	Yes	B cell function and immunoglobulins	4	3,72	6,6E-04	3,9E-05	Igkc/Ighm/Ighv11-2/Iglc1
GO:1990869	regulation of B cell activation	Yes	B cell function and immunoglobulins	4	3,72	1,6E-03	1,3E-04	Igkc/Ighm/Ighv11-2/Iglc1
GO:0030595	adaptive immune response based on somatic recombination of immune receptor	Yes	B cell function and immunoglobulins	5	4,65	1,6E-03	1,5E-04	Fcer1a/Igkc/Ighm/Ighv11-2/Iglc1
GO:0002443	B cell activation	Yes	B cell function and immunoglobulins	4	3,72	6,4E-03	1,0E-03	Igkc/Ighm/Ighv11-2/Iglc1
GO:0002279	extracellular matrix disassembly	Yes	ECM	2	1,86	7,4E-03	1,2E-03	Cma1/Tpsb2
GO:1990849	leukocyte mediated immunity	Yes	Immune activation and immune processess	7	6,51	2,1E-04	2,9E-06	Fcer1a/Igkc/Mrgprb1/Mrgprx2/Ighm/Ighv11-2/Iglc1
GO:0006956	complement activation, classical pathway	Yes	Immune activation and immune processess	4	3,72	2,5E-04	4,6E-06	Igkc/Ighm/Ighv11-2/Iglc1
GO:0006911	complement activation	Yes	Immune activation and immune processess	4	3,72	3,4E-04	1,3E-05	Igkc/Ighm/Ighv11-2/Iglc1
GO:0050867	lymphocyte mediated immunity	Yes	Immune activation and immune processess	5	4,65	1,6E-03	1,4E-04	Fcer1a/Igkc/Ighm/Ighv11-2/Iglc1
GO:0043547	cell recognition	Yes	Immune activation and immune processess	4	3,72	1,6E-03	1,5E-04	Igkc/Ighm/Ighv11-2/Iglc1
GO:0045055	antigen receptor-mediated signaling pathway	Yes	Immune activation and immune processess	4	3,72	2,0E-03	2,0E-04	Igkc/Ighm/Ighv11-2/Iglc1
GO:0002429	leukocyte degranulation	Yes	Immune activation and immune processess	3	2,79	2,2E-03	2,4E-04	Fcer1a/Mrgprb1/Mrgprx2
GO:0002757	positive regulation of leukocyte activation	Yes	Immune activation and immune processess	5	4,65	2,2E-03	2,6E-04	Fcer1a/Igkc/Ighm/Ighv11-2/Iglc1
GO:2000756	immune response-activating signal transduction	Yes	Immune activation and immune processess	4	3,72	2,8E-03	3,6E-04	Igkc/Ighm/Ighv11-2/Iglc1
GO:0051781	immune response-activating cell surface receptor signaling pathway	Yes	Immune activation and immune processess	4	3,72	2,8E-03	3,6E-04	Igkc/Ighm/Ighv11-2/Iglc1
GO:0045661	immune response-regulating cell surface receptor signaling pathway	Yes	Immune activation and immune processess	4	3,72	3,0E-03	4,2E-04	Igkc/Ighm/Ighv11-2/Iglc1
GO:0032465	activation of immune response	Yes	Immune activation and immune processess	4	3,72	7,1E-03	1,2E-03	Igkc/Ighm/Ighv11-2/Iglc1
GO:0072676	positive regulation of lymphocyte activation	Yes	Immune activation and immune processess	4	3,72	8,8E-03	1,6E-03	Igkc/Ighm/Ighv11-2/Iglc1
GO:0002366	immune response-regulating signaling pathway	Yes	Immune activation and immune processess	4	3,72	1,2E-02	2,5E-03	Igkc/Ighm/Ighv11-2/Iglc1
GO:0002275	leukocyte activation involved in immune response	Yes	Immune activation and immune processess	3	2,79	3,0E-02	7,0E-03	Fcer1a/Mrgprb1/Mrgprx2
GO:1901983	cell activation involved in immune response	Yes	Immune activation and immune processess	3	2,79	3,0E-02	7,3E-03	Fcer1a/Mrgprb1/Mrgprx2
GO:0045576	lymphocyte chemotaxis	Yes	Lymphoid cell chemotaxis/migration	3	2,79	6,9E-04	4,2E-05	Ccl8/Ccl9/Ccl6
GO:0043299	leukocyte chemotaxis	Yes	Lymphoid cell chemotaxis/migration	4	3,72	2,1E-03	2,1E-04	Pf4/Ccl8/Ccl9/Ccl6
GO:0097529	lymphocyte migration	Yes	Lymphoid cell chemotaxis/migration	3	2,79	2,8E-03	3,7E-04	Ccl8/Ccl9/Ccl6
GO:0002696	cell chemotaxis	Yes	Lymphoid cell chemotaxis/migration	4	3,72	4,2E-03	6,3E-04	Pf4/Ccl8/Ccl9/Ccl6
GO:0032418	leukocyte migration	Yes	Lymphoid cell chemotaxis/migration	4	3,72	8,5E-03	1,5E-03	Pf4/Ccl8/Ccl9/Ccl6
GO:0070555	mononuclear cell migration	Yes	Lymphoid cell chemotaxis/migration	3	2,79	9,6E-03	1,8E-03	Ccl8/Ccl9/Ccl6
GO:0071346	icosanoid metabolic process	Yes	Metabolism	2	1,86	3,4E-02	8,5E-03	Fcer1a/Alox12e
GO:0050864	neutrophil chemotaxis	Yes	Myeloid cell chemotaxis/migration	4	3,72	3,0E-04	9,7E-06	Pf4/Ccl8/Ccl9/Ccl6
GO:0002460	neutrophil migration	Yes	Myeloid cell chemotaxis/migration	4	3,72	4,8E-04	2,4E-05	Pf4/Ccl8/Ccl9/Ccl6
GO:0002449	granulocyte chemotaxis	Yes	Myeloid cell chemotaxis/migration	4	3,72	4,8E-04	2,4E-05	Pf4/Ccl8/Ccl9/Ccl6
GO:0071347	granulocyte migration	Yes	Myeloid cell chemotaxis/migration	4	3,72	8,1E-04	5,4E-05	Pf4/Ccl8/Ccl9/Ccl6
GO:0060326	monocyte chemotaxis	Yes	Myeloid cell chemotaxis/migration	3	2,79	9,0E-04	6,3E-05	Ccl8/Ccl9/Ccl6
GO:0034341	myeloid leukocyte migration	Yes	Myeloid cell chemotaxis/migration	4	3,72	2,2E-03	2,6E-04	Pf4/Ccl8/Ccl9/Ccl6
GO:0042113	mast cell degranulation	Yes	Myeloid immunity	3	2,79	1,1E-03	7,7E-05	Fcer1a/Mrgprb1/Mrgprx2
GO:0060142	mast cell mediated immunity	Yes	Myeloid immunity	3	2,79	1,1E-03	8,4E-05	Fcer1a/Mrgprb1/Mrgprx2
GO:0002253	mast cell activation involved in immune response	Yes	Myeloid immunity	3	2,79	1,2E-03	9,3E-05	Fcer1a/Mrgprb1/Mrgprx2
GO:0022617	mast cell activation	Yes	Myeloid immunity	3	2,79	2,1E-03	2,2E-04	Fcer1a/Mrgprb1/Mrgprx2
GO:0001990	myeloid cell activation involved in immune response	Yes	Myeloid immunity	3	2,79	3,0E-03	4,2E-04	Fcer1a/Mrgprb1/Mrgprx2
GO:0032467	myeloid leukocyte mediated immunity	Yes	Myeloid immunity	3	2,79	3,7E-03	5,4E-04	Fcer1a/Mrgprb1/Mrgprx2
GO:0071356	myeloid leukocyte activation	Yes	Myeloid immunity	3	2,79	2,2E-02	4,7E-03	Fcer1a/Mrgprb1/Mrgprx2
GO:0002263	phagocytosis, recognition	Yes	Phagocytosis	4	3,72	2,5E-04	6,1E-06	Igkc/Ighm/Ighv11-2/Iglc1
GO:0050886	phagocytosis, engulfment	Yes	Phagocytosis	4	3,72	4,3E-04	1,8E-05	Igkc/Ighm/Ighv11-2/Iglc1
GO:0070372	plasma membrane invagination	Yes	Phagocytosis	4	3,72	4,8E-04	2,4E-05	Igkc/Ighm/Ighv11-2/Iglc1
GO:0006690	membrane invagination	Yes	Phagocytosis	4	3,72	5,8E-04	3,1E-05	Igkc/Ighm/Ighv11-2/Iglc1
GO:0003073	phagocytosis	Yes	Phagocytosis	5	4,65	6,6E-04	3,9E-05	Igkc/Cebpe/Ighm/Ighv11-2/Iglc1
GO:0070371	regulation of syncytium formation by plasma membrane fusion	Yes	Phagocytosis	2	1,86	6,5E-03	1,0E-03	Ccl8/Nfe2
GO:0045445	syncytium formation by plasma membrane fusion	Yes	Phagocytosis	2	1,86	1,9E-02	3,9E-03	Ccl8/Nfe2
GO:0043087	cell-cell fusion	Yes	Phagocytosis	2	1,86	1,9E-02	3,9E-03	Ccl8/Nfe2
GO:0099024	response to chemokine	Yes	Response to cytokine/chemokine	4	3,72	2,1E-04	2,5E-06	Pf4/Ccl8/Ccl9/Ccl6
GO:0071621	chemokine-mediated signaling pathway	Yes	Response to cytokine/chemokine	4	3,72	2,1E-04	1,3E-06	Pf4/Ccl8/Ccl9/Ccl6
GO:1990266	cellular response to chemokine	Yes	Response to cytokine/chemokine	4	3,72	2,1E-04	2,5E-06	Pf4/Ccl8/Ccl9/Ccl6
GO:0010324	cellular response to interleukin-1	Yes	Response to cytokine/chemokine	3	2,79	1,6E-03	1,5E-04	Ccl8/Ccl9/Ccl6
GO:0050871	response to interleukin-1	Yes	Response to cytokine/chemokine	3	2,79	2,2E-03	2,7E-04	Ccl8/Ccl9/Ccl6
GO:0006909	cellular response to interferon-gamma	Yes	Response to cytokine/chemokine	3	2,79	3,0E-03	4,2E-04	Ccl8/Ccl9/Ccl6
GO:0048247	response to interferon-gamma	Yes	Response to cytokine/chemokine	3	2,79	5,0E-03	7,6E-04	Ccl8/Ccl9/Ccl6
GO:0097530	cellular response to tumor necrosis factor	Yes	Response to cytokine/chemokine	3	2,79	8,1E-03	1,4E-03	Ccl8/Ccl9/Ccl6
GO:0002548	cytokine-mediated signaling pathway	Yes	Response to cytokine/chemokine	4	3,72	9,5E-03	1,7E-03	Pf4/Ccl8/Ccl9/Ccl6
GO:0043303	response to tumor necrosis factor	Yes	Response to cytokine/chemokine	3	2,79	9,9E-03	1,9E-03	Ccl8/Ccl9/Ccl6
GO:0042742	positive regulation of cytokinesis	Yes	Response to cytokine/chemokine	2	1,86	7,9E-03	1,4E-03	Mrgprb1/Mrgprx2
GO:0002448	regulation of cytokinesis	Yes	Response to cytokine/chemokine	2	1,86	2,7E-02	6,4E-03	Mrgprb1/Mrgprx2
GO:0006958	regulation of systemic arterial blood pressure by circulatory renin-angiotensin	No	biological processess	2	1,86	2,0E-03	1,9E-04	Cpa3/Mcpt4
GO:0016064	regulation of systemic arterial blood pressure by renin-angiotensin	No	biological processess	2	1,86	3,8E-03	5,7E-04	Cpa3/Mcpt4
GO:0006910	regulation of systemic arterial blood pressure by hormone	No	biological processess	2	1,86	7,7E-03	1,3E-03	Cpa3/Mcpt4
GO:0019724	regulation of systemic arterial blood pressure mediated by a chemical signal	No	biological processess	2	1,86	1,0E-02	1,9E-03	Cpa3/Mcpt4
GO:0019730	regulation of myoblast differentiation	No	biological processess	2	1,86	2,5E-02	5,8E-03	Ccl8/Ccl9
GO:0002455	endocrine process	No	biological processess	2	1,86	3,2E-02	7,7E-03	Cpa3/Mcpt4
GO:0030593	regulation of systemic arterial blood pressure	No	biological processess	2	1,86	3,7E-02	9,5E-03	Cpa3/Mcpt4
GO:0050853	myoblast differentiation	No	biological processess	2	1,86	4,1E-02	1,0E-02	Ccl8/Ccl9
GO:0002768	antimicrobial humoral response	No	microbial	4	3,72	3,0E-04	1,1E-05	Jchain/Pf4/Ccl8/Ighm
GO:0043410	defense response to bacterium	No	microbial	5	4,65	8,0E-04	5,2E-05	Jchain/Igkc/Ighm/Ighv11-2/Iglc1
GO:0002444	antibacterial humoral response	No	microbial	2	1,86	1,0E-02	2,0E-03	Jchain/Ighm
GO:0003081	antimicrobial humoral immune response mediated by antimicrobial peptide	No	microbial	2	1,86	2,0E-02	4,3E-03	Pf4/Ccl8
GO:0050900	lysosome localization	No	other	3	2,79	2,2E-03	2,7E-04	Fcer1a/Mrgprb1/Mrgprx2
GO:0051251	vacuolar localization	No	other	3	2,79	2,2E-03	2,7E-04	Fcer1a/Mrgprb1/Mrgprx2
GO:0019221	positive regulation of cell activation	No	other	5	4,65	2,6E-03	3,3E-04	Fcer1a/Igkc/Ighm/Ighv11-2/Iglc1
GO:0071674	positive regulation of MAPK cascade	No	other	5	4,65	3,2E-03	4,6E-04	Fcer1a/Ccl8/Ccl9/Ccl6/Ighm
GO:0006887	exocytosis	No	other	4	3,72	9,7E-03	1,8E-03	Fcer1a/Mrgprb1/Mrgprx2/Ccl8
GO:0034612	positive regulation of ERK1 and ERK2 cascade	No	other	3	2,79	1,3E-02	2,7E-03	Ccl8/Ccl9/Ccl6
GO:0003044	syncytium formation	No	other	2	1,86	1,9E-02	4,1E-03	Ccl8/Nfe2
GO:0019731	positive regulation of GTPase activity	No	other	3	2,79	2,3E-02	5,0E-03	Ccl8/Ccl9/Ccl6
GO:0002764	regulated exocytosis	No	other	3	2,79	2,5E-02	5,4E-03	Fcer1a/Mrgprb1/Mrgprx2
GO:0070374	positive regulation of cell division	No	other	2	1,86	2,5E-02	5,6E-03	Mrgprb1/Mrgprx2
GO:0000768	regulation of peptidyl-lysine acetylation	No	other	2	1,86	2,5E-02	5,7E-03	Msx3/Nfe2
GO:0140253	regulation of protein acetylation	No	other	2	1,86	3,0E-02	7,2E-03	Msx3/Nfe2
GO:0006949	regulation of ERK1 and ERK2 cascade	No	other	3	2,79	3,2E-02	7,8E-03	Ccl8/Ccl9/Ccl6
GO:0061844	ERK1 and ERK2 cascade	No	other	3	2,79	3,7E-02	9,5E-03	Ccl8/Ccl9/Ccl6
GO:0002274	regulation of GTPase activity	No	other	3	2,79	4,4E-02	1,1E-02	Ccl8/Ccl9/Ccl6

Supplementary table 5. Gene Ontology (GO) enrichment analysis for biological processes associated with Pan02 tumors from mice treated with an anti-IL6R antibody compared to Pan02 tumors from untreated mice (Supplementary table 1).

ID	Description	Immune related	Category	Count	%	p.adjust	pvalue	geneID
GO:0045765	regulation of angiogenesis	Yes	Angiogenesis	8	5,3	3,5E-02	2,8E-03	Hc/Ccl24/Gata2/Alox5/Lgals3/Cma1/Ccr3/Enpp2
GO:0045766	positive regulation of angiogenesis	Yes	Angiogenesis	6	4	3,7E-02	3,0E-03	Hc/Ccl24/Gata2/Lgals3/Cma1/Ccr3
GO:1904018	positive regulation of vasculature development	Yes	Angiogenesis	6	4	3,7E-02	3,0E-03	Hc/Ccl24/Gata2/Lgals3/Cma1/Ccr3
GO:1901342	regulation of vasculature development	Yes	Angiogenesis	8	5,3	3,7E-02	3,1E-03	Hc/Ccl24/Gata2/Alox5/Lgals3/Cma1/Ccr3/Enpp2
GO:0001936	regulation of endothelial cell proliferation	Yes	Angiogenesis	6	4	1,9E-02	1,3E-03	Ccl24/Gata2/Alox5/Arg1/Aldh1a2/Ccr3
GO:2000352	negative regulation of endothelial cell apoptotic process	Yes	Angiogenesis	3	2	2,8E-02	2,1E-03	Gata3/Gata2/Il13
GO:0001935	endothelial cell proliferation	Yes	Angiogenesis	6	4	3,0E-02	2,3E-03	Ccl24/Gata2/Alox5/Arg1/Aldh1a2/Ccr3
GO:0019724	B cell mediated immunity	Yes	B cell function and immunoglobulins	11	7,3	3,4E-05	8,0E-07	Fcer1a/Susd4/Hc/Il13ra2/Igkc/Ighm/Ighv2-2/Ighv11-2/Ighv1-55/Iglc1/Iglc2
GO:0050853	B cell receptor signaling pathway	Yes	B cell function and immunoglobulins	7	4,6	6,6E-04	2,3E-05	Igkc/Ighm/Ighv2-2/Ighv11-2/Ighv1-55/Iglc1/Iglc2
GO:0050871	positive regulation of B cell activation	Yes	B cell function and immunoglobulins	8	5,3	6,6E-04	2,3E-05	Igkc/Il13/Ighm/Ighv2-2/Ighv11-2/Ighv1-55/Iglc1/Iglc2
GO:0050864	regulation of B cell activation	Yes	B cell function and immunoglobulins	8	5,3	4,2E-03	2,2E-04	Igkc/Il13/Ighm/Ighv2-2/Ighv11-2/Ighv1-55/Iglc1/Iglc2
GO:0042113	B cell activation	Yes	B cell function and immunoglobulins	9	6	2,5E-02	1,8E-03	Kit/Igkc/Il13/Ighm/Ighv2-2/Ighv11-2/Ighv1-55/Iglc1/Iglc2
GO:0002455	humoral immune response mediated by circulating immunoglobulin	Yes	B cell function and immunoglobulins	9	6	7,0E-06	1,1E-07	Susd4/Hc/Igkc/Ighm/Ighv2-2/Ighv11-2/Ighv1-55/Iglc1/Iglc2
GO:0002460	adaptive immune response based on somatic recombination of immune rec	Yes	B cell function and immunoglobulins	16	10,6	7,3E-06	1,1E-07	Il1rl1/Fcer1a/Susd4/Gata3/Hc/Il13ra2/Igkc/Arg1/Gzmb/Ighm/Ighv2-2/Ighv11-2/Ighv1-55/Iglc1/Iglc2/H2-M2
GO:0016064	immunoglobulin mediated immune response	Yes	B cell function and immunoglobulins	11	7,3	3,1E-05	6,9E-07	Fcer1a/Susd4/Hc/Il13ra2/Igkc/Ighm/Ighv2-2/Ighv11-2/Ighv1-55/Iglc1/Iglc2
GO:0002377	immunoglobulin production	Yes	B cell function and immunoglobulins	12	7,9	4,4E-05	1,2E-06	Il13ra2/Igkv14-126/Igkv9-120/Igkv19-93/Igkv4-86/Igkv12-46/Igkv8-19/Igkv6-17/Igkv3-12/Il13/Ighm/Iglv3
GO:0031640	killing of cells of another organism	Yes	Cell killing	5	3,3	1,0E-03	4,1E-05	Lyz1/Ccl22/Ccl17/Lgals3/Ccl8
GO:0001906	cell killing	Yes	Cell killing	9	6	1,3E-03	5,8E-05	Lyz1/Arg1/Ccl22/Ccl17/Lgals3/Gzmb/Il13/Ccl8/H2-M2
GO:0006956	complement activation	Yes	Complement	10	6,6	2,0E-06	2,1E-08	Susd4/Hc/Igkc/Ighm/Ighv2-2/Ighv11-2/Ighv1-55/Iglc1/Iglc2/Masp1
GO:0006958	complement activation, classical pathway	Yes	Complement	9	6	2,1E-06	2,7E-08	Susd4/Hc/Igkc/Ighm/Ighv2-2/Ighv11-2/Ighv1-55/Iglc1/Iglc2
GO:0006957	complement activation, alternative pathway	Yes	Complement	2	1,3	4,6E-02	4,2E-03	Susd4/Hc
GO:0022617	extracellular matrix disassembly	Yes	ECM	3	2	4,3E-02	3,7E-03	Cma1/Tpsab1/Tpsb2
GO:0017157	regulation of exocytosis	Yes	Exocytosis	10	6,6	7,3E-04	2,8E-05	Fcer1a/Il13ra2/Gata1/Adora3/Gata2/Il13/P2rx1/Nrn1/Syt12/Ms4a2
GO:0007205	protein kinase C-activating G protein-coupled receptor signaling pathway	Yes	Exocytosis	4	2,6	1,2E-03	4,9E-05	Edn2/Cish/Cck/Ms4a2
GO:1903532	positive regulation of secretion by cell	Yes	Exocytosis	9	6	2,4E-02	1,7E-03	Fcer1a/Gata1/Adora3/Edn2/Gata2/Lgals3/Cck/Il13/Ms4a2
GO:0051047	positive regulation of secretion	Yes	Exocytosis	9	6	3,8E-02	3,1E-03	Fcer1a/Gata1/Adora3/Edn2/Gata2/Lgals3/Cck/Il13/Ms4a2
GO:0045921	positive regulation of exocytosis	Yes	Exocytosis	6	4	3,5E-03	1,8E-04	Fcer1a/Gata1/Adora3/Gata2/Il13/Ms4a2
GO:0045055	regulated exocytosis	Yes	Exocytosis	16	10,6	9,3E-08	6,2E-10	Fcer1a/Ptgds/Il13ra2/Gata1/Adora3/Kit/Gata2/Tph1/Mrgprb1/Mrgprx2/Mrgprb2/Il13/P2rx1/Nrn1/Syt12/Ms4a2
GO:0006887	exocytosis	Yes	Exocytosis	17	11,3	2,1E-06	2,5E-08	Fcer1a/Ptgds/Il13ra2/Gata1/Adora3/Kit/Gata2/Tph1/Mrgprb1/Mrgprx2/Mrgprb2/Il13/Ccl8/P2rx1/Nrn1/Syt12/Ms4a2
GO:0006959	humoral immune response	Yes	Immune activation and immune processess	20	13,2	1,7E-11	1,6E-14	Susd4/Gata3/Slpi/Hc/Jchain/Pf4/Igkc/Alox5/Ccl22/Ccl17/Lgals3/Trf/Ccl8/Ighm/Ighv2-2/Ighv11-2/Ighv1-55/Iglc1/Iglc2/Masp1
GO:0002440	production of molecular mediator of immune response	Yes	Immune activation and immune processess	17	11,3	2,0E-06	2,1E-08	Fcer1a/Gata3/Prg2/Il13ra2/Kit/Igkv14-126/Igkv9-120/Igkv19-93/Igkv4-86/Igkv12-46/Igkv8-19/Igkv6-17/Igkv3-12/Arg1/Il13/Ighm/Iglv3
GO:0002263	cell activation involved in immune response	Yes	Immune activation and immune processess	15	9,9	2,0E-06	2,2E-08	Fcer1a/Gata3/Ptgds/Il13ra2/Gata1/Adora3/Kit/Gata2/Mrgprb1/Mrgprx2/Mrgprb2/Enpp3/Lgals3/Il13/Ms4a2
GO:0002697	regulation of immune effector process	Yes	Immune activation and immune processess	17	11,3	6,9E-06	1,0E-07	Fcer1a/Susd4/Gata3/Prg2/Il13ra2/Gata1/Adora3/Kit/Gata2/Enpp3/Arg1/Lgals3/Il13/Ighm/Masp1/H2-M2/Ms4a2
GO:0008037	cell recognition	Yes	Immune activation and immune processess	11	7,3	3,9E-05	9,3E-07	Igkc/Lgals3/Ighm/Ighv2-2/Ighv11-2/Ighv1-55/Iglc1/Iglc2/Fetub/Masp1/Hspa1b
GO:0050727	regulation of inflammatory response	Yes	Immune activation and immune processess	14	9,3	4,1E-05	1,0E-06	Il1rl1/Fcer1a/Gata3/Duoxa2/Adora3/Pla2g2d/Ccl24/Alox5/Enpp3/Slc7a2/Cma1/Il13/Alox15/Cd200r3
GO:0002699	positive regulation of immune effector process	Yes	Immune activation and immune processess	13	8,6	4,2E-05	1,1E-06	Fcer1a/Gata3/Il13ra2/Gata1/Adora3/Kit/Gata2/Enpp3/Arg1/Il13/Masp1/H2-M2/Ms4a2
GO:0050867	positive regulation of cell activation	Yes	Immune activation and immune processess	16	10,6	4,3E-05	1,1E-06	Il1rl1/Fcer1a/Gata3/Gata1/Adora3/Gata2/Igkc/Il13/Ighm/Ighv2-2/Ighv11-2/Ighv1-55/Iglc1/Iglc2/Pdcd1lg2/Ms4a2
GO:0002768	immune response-regulating cell surface receptor signaling pathway	Yes	Immune activation and immune processess	12	7,9	6,6E-05	2,0E-06	Gata3/Kit/Igkc/Lgals3/Ighm/Ighv2-2/Ighv11-2/Ighv1-55/Iglc1/Iglc2/Masp1/Ms4a2
GO:0002683	negative regulation of immune system process	Yes	Immune activation and immune processess	15	9,9	1,5E-04	4,8E-06	Il1rl1/Susd4/Prg2/Hc/Il13ra2/Pla2g2d/Gata2/Tarm1/Enpp3/Arg1/Lgals3/Alox15/Tigit/Masp1/Pdcd1lg2
GO:0002253	activation of immune response	Yes	Immune activation and immune processess	12	7,9	7,9E-04	3,0E-05	Susd4/Gata3/Hc/Igkc/Lgals3/Ighm/Ighv2-2/Ighv11-2/Ighv1-55/Iglc1/Iglc2/Masp1
GO:0002429	immune response-activating cell surface receptor signaling pathway	Yes	Immune activation and immune processess	10	6,6	1,2E-03	4,8E-05	Gata3/Igkc/Lgals3/Ighm/Ighv2-2/Ighv11-2/Ighv1-55/Iglc1/Iglc2/Masp1
GO:0002757	immune response-activating signal transduction	Yes	Immune activation and immune processess	10	6,6	1,2E-03	5,0E-05	Gata3/Igkc/Lgals3/Ighm/Ighv2-2/Ighv11-2/Ighv1-55/Iglc1/Iglc2/Masp1
GO:0050851	antigen receptor-mediated signaling pathway	Yes	Immune activation and immune processess	9	6	1,8E-03	8,0E-05	Gata3/Igkc/Lgals3/Ighm/Ighv2-2/Ighv11-2/Ighv1-55/Iglc1/Iglc2
GO:0002532	production of molecular mediator involved in inflammatory response	Yes	Immune activation and immune processess	6	4	2,5E-03	1,2E-04	Adora3/Chil5/Alox5/Tarm1/Slc7a2/P2rx1
GO:0002698	negative regulation of immune effector process	Yes	Immune activation and immune processess	7	4,6	2,9E-03	1,4E-04	Susd4/Prg2/Il13ra2/Enpp3/Arg1/Lgals3/Masp1
GO:0050777	negative regulation of immune response	Yes	Immune activation and immune processess	8	5,3	3,3E-03	1,6E-04	Il1rl1/Susd4/Il13ra2/Enpp3/Arg1/Lgals3/Alox15/Masp1
GO:0002764	immune response-regulating signaling pathway	Yes	Immune activation and immune processess	12	7,9	4,1E-03	2,2E-04	Gata3/Kit/Igkc/Lgals3/Ighm/Ighv2-2/Ighv11-2/Ighv1-55/Iglc1/Iglc2/Masp1/Ms4a2
GO:0002700	regulation of production of molecular mediator of immune response	Yes	Immune activation and immune processess	8	5,3	4,5E-03	2,5E-04	Fcer1a/Gata3/Prg2/Il13ra2/Kit/Arg1/Il13/Ighm
GO:0032944	regulation of mononuclear cell proliferation	Yes	Immune activation and immune processess	8	5,3	1,9E-02	1,2E-03	Tnfrsf9/Pla2g2d/Tarm1/Arg1/Lgals3/Il13/Ighm/Pdcd1lg2
GO:0002820	negative regulation of adaptive immune response	Yes	Immune activation and immune processess	4	2,6	2,2E-02	1,5E-03	Il1rl1/Susd4/Arg1/Alox15
GO:0002819	regulation of adaptive immune response	Yes	Immune activation and immune processess	7	4,6	3,1E-02	2,4E-03	Il1rl1/Fcer1a/Susd4/Gata3/Arg1/Alox15/H2-M2
GO:0031349	positive regulation of defense response	Yes	Immune activation and immune processess	8	5,3	3,7E-02	3,0E-03	Il1rl1/Fcer1a/Adora3/Penk/Ccl24/Arg1/Cck/Masp1
GO:0031348	negative regulation of defense response	Yes	Immune activation and immune processess	7	4,6	3,8E-02	3,1E-03	Susd4/Gata3/Alox5/Enpp3/Arg1/Cck/Il13
GO:0002443	leukocyte mediated immunity	Yes	Leukocyte/lymphocyte function	25	16,6	1,0E-10	2,5E-13	Fcer1a/Susd4/Gata3/Ptgds/Hc/Il13ra2/Gata1/Adora3/Kit/Gata2/Igkc/Mrgprb1/Mrgprx2/Mrgprb2/Arg1/Gzmb/Il13/Ighm/Ighv2-2/Ighv11-2/Ighv1-55/Iglc1/Iglc2/H2-M2/Ms4
GO:0043299	leukocyte degranulation	Yes	Leukocyte/lymphocyte function	12	7,9	2,3E-09	8,7E-12	Fcer1a/Ptgds/Il13ra2/Gata1/Adora3/Kit/Gata2/Mrgprb1/Mrgprx2/Mrgprb2/Il13/Ms4a2
GO:0002366	leukocyte activation involved in immune response	Yes	Leukocyte/lymphocyte function	15	9,9	2,0E-06	1,9E-08	Fcer1a/Gata3/Ptgds/Il13ra2/Gata1/Adora3/Kit/Gata2/Mrgprb1/Mrgprx2/Mrgprb2/Enpp3/Lgals3/Il13/Ms4a2
GO:0043300	regulation of leukocyte degranulation	Yes	Leukocyte/lymphocyte function	7	4,6	2,3E-05	4,2E-07	Fcer1a/Il13ra2/Gata1/Adora3/Gata2/Il13/Ms4a2
GO:0043302	positive regulation of leukocyte degranulation	Yes	Leukocyte/lymphocyte function	6	4	2,4E-05	4,5E-07	Fcer1a/Gata1/Adora3/Gata2/Il13/Ms4a2
GO:0002449	lymphocyte mediated immunity	Yes	Leukocyte/lymphocyte function	15	9,9	2,6E-05	5,4E-07	Fcer1a/Susd4/Gata3/Hc/Il13ra2/Igkc/Arg1/Gzmb/Ighm/Ighv2-2/Ighv11-2/Ighv1-55/Iglc1/Iglc2/H2-M2
GO:0002696	positive regulation of leukocyte activation	Yes	Leukocyte/lymphocyte function	16	10,6	2,9E-05	6,1E-07	Il1rl1/Fcer1a/Gata3/Gata1/Adora3/Gata2/Igkc/Il13/Ighm/Ighv2-2/Ighv11-2/Ighv1-55/Iglc1/Iglc2/Pdcd1lg2/Ms4a2
GO:0002703	regulation of leukocyte mediated immunity	Yes	Leukocyte/lymphocyte function	12	7,9	1,2E-04	3,9E-06	Fcer1a/Susd4/Gata3/Il13ra2/Gata1/Adora3/Kit/Gata2/Arg1/Il13/H2-M2/Ms4a2
GO:0070663	regulation of leukocyte proliferation	Yes	Leukocyte/lymphocyte function	10	6,6	2,1E-03	9,6E-05	Tnfrsf9/Pla2g2d/Kit/Tarm1/Enpp3/Arg1/Lgals3/Il13/Ighm/Pdcd1lg2
GO:0002695	negative regulation of leukocyte activation	Yes	Leukocyte/lymphocyte function	8	5,3	4,4E-03	2,4E-04	Il13ra2/Pla2g2d/Tarm1/Enpp3/Arg1/Lgals3/Tigit/Pdcd1lg2
GO:0050868	negative regulation of T cell activation	Yes	Leukocyte/lymphocyte function	6	4	1,3E-02	7,9E-04	Pla2g2d/Tarm1/Arg1/Lgals3/Tigit/Pdcd1lg2
GO:0070661	leukocyte proliferation	Yes	Leukocyte/lymphocyte function	10	6,6	1,3E-02	8,3E-04	Tnfrsf9/Pla2g2d/Kit/Tarm1/Enpp3/Arg1/Lgals3/Il13/Ighm/Pdcd1lg2
GO:0050670	regulation of lymphocyte proliferation	Yes	Leukocyte/lymphocyte function	8	5,3	1,7E-02	1,1E-03	Tnfrsf9/Pla2g2d/Tarm1/Arg1/Lgals3/Il13/Ighm/Pdcd1lg2
GO:0051251	positive regulation of lymphocyte activation	Yes	Leukocyte/lymphocyte function	10	6,6	1,7E-02	1,1E-03	Gata3/Igkc/Il13/Ighm/Ighv2-2/Ighv11-2/Ighv1-55/Iglc1/Iglc2/Pdcd1lg2
GO:1903038	negative regulation of leukocyte cell-cell adhesion	Yes	Leukocyte/lymphocyte function	6	4	1,9E-02	1,3E-03	Pla2g2d/Tarm1/Arg1/Lgals3/Tigit/Pdcd1lg2
GO:0070664	negative regulation of leukocyte proliferation	Yes	Leukocyte/lymphocyte function	5	3,3	2,2E-02	1,6E-03	Pla2g2d/Tarm1/Enpp3/Arg1/Pdcd1lg2
GO:0051250	negative regulation of lymphocyte activation	Yes	Leukocyte/lymphocyte function	6	4	3,3E-02	2,6E-03	Pla2g2d/Tarm1/Arg1/Lgals3/Tigit/Pdcd1lg2
GO:0042130	negative regulation of T cell proliferation	Yes	Leukocyte/lymphocyte function	4	2,6	4,3E-02	3,8E-03	Pla2g2d/Tarm1/Arg1/Pdcd1lg2
GO:2000551	regulation of T-helper 2 cell cytokine production	Yes	Leukocyte/lymphocyte function	2	1,3	4,6E-02	4,2E-03	Gata3/Arg1
GO:0030595	leukocyte chemotaxis	Yes	Lymphoid cell chemotaxis/migration	15	9,9	7,1E-08	4,4E-10	Hc/Edn2/Kit/Pf4/Ccl24/Alox5/F7/Ccl22/Ccl17/Lgals3/Ccr3/Ccl8/Ccl9/Ccl6/Retnlg
GO:0048247	lymphocyte chemotaxis	Yes	Lymphoid cell chemotaxis/migration	6	4	1,1E-04	3,3E-06	Ccl24/Ccl22/Ccl17/Ccl8/Ccl9/Ccl6
GO:0050900	leukocyte migration	Yes	Lymphoid cell chemotaxis/migration	17	11,3	1,4E-06	1,2E-08	Gata3/Hc/Adora3/Edn2/Kit/Pf4/Ccl24/Alox5/F7/Ccl22/Ccl17/Lgals3/Ccr3/Ccl8/Ccl9/Ccl6/Retnlg
GO:0071674	mononuclear cell migration	Yes	Lymphoid cell chemotaxis/migration	10	6,6	7,3E-05	2,2E-06	Gata3/Ccl24/Alox5/Ccl22/Ccl17/Lgals3/Ccl8/Ccl9/Ccl6/Retnlg

GO:0072676	lymphocyte migration	Yes	Lymphoid cell chemotaxis/migration	7	4,6	6,7E-04	2,4E-05	Gata3/Ccl24/Ccl22/Ccl17/Ccl8/Ccl9/Ccl6
GO:0006636	unsaturated fatty acid biosynthetic process	Yes	Metabolism	4	2,6	5,8E-03	3,2E-04	Ptgds/Ptgs1/Edn2/Alox15
GO:0043651	linoleic acid metabolic process	Yes	Metabolism	3	2	6,1E-03	3,4E-04	Alox5/Alox12e/Alox15
GO:0046394	carboxylic acid biosynthetic process	Yes	Metabolism	9	6	6,7E-03	3,9E-04	Fcer1a/Ptgds/Ptgs1/Edn2/Alox5/Aldh1a2/Ltc4s/Alox12e/Alox15
GO:0016053	organic acid biosynthetic process	Yes	Metabolism	9	6	6,8E-03	4,0E-04	Fcer1a/Ptgds/Ptgs1/Edn2/Alox5/Aldh1a2/Ltc4s/Alox12e/Alox15
GO:0008211	glucocorticoid metabolic process	Yes	Metabolism	3	2	1,1E-02	6,5E-04	Hsd11b1/Cyp11a1/Cyp11b1
GO:0006691	leukotriene metabolic process	Yes	Metabolism	3	2	1,2E-02	7,5E-04	Fcer1a/Alox5/Ltc4s
GO:0001516	prostaglandin biosynthetic process	Yes	Metabolism	3	2	2,4E-02	1,7E-03	Ptgds/Ptgs1/Edn2
GO:0046457	prostanoid biosynthetic process	Yes	Metabolism	3	2	2,4E-02	1,7E-03	Ptgds/Ptgs1/Edn2
GO:0006690	icosanoid metabolic process	Yes	Metabolism	9	6	9,6E-06	1,6E-07	Fcer1a/Ptgds/Ptgs1/Edn2/Alox5/Cyp2e1/Ltc4s/Alox12e/Alox15
GO:0120254	olefinic compound metabolic process	Yes	Metabolism	9	6	2,4E-05	4,6E-07	Ptgs1/Lrat/Alox5/Cyp2e1/Cyp11a1/Aldh1a2/Alox12e/Alox15/Cyp11b1
GO:0046456	icosanoid biosynthetic process	Yes	Metabolism	6	4	5,7E-05	1,5E-06	Fcer1a/Ptgds/Ptgs1/Edn2/Alox5/Ltc4s
GO:0033559	unsaturated fatty acid metabolic process	Yes	Metabolism	7	4,6	3,7E-04	1,3E-05	Ptgds/Ptgs1/Edn2/Alox5/Cyp2e1/Alox12e/Alox15
GO:0042759	long-chain fatty acid biosynthetic process	Yes	Metabolism	4	2,6	6,6E-04	2,4E-05	Alox5/Ltc4s/Alox12e/Alox15
GO:0019369	arachidonic acid metabolic process	Yes	Metabolism	5	3,3	7,1E-04	2,7E-05	Ptgs1/Alox5/Cyp2e1/Alox12e/Alox15
GO:0001676	long-chain fatty acid metabolic process	Yes	Metabolism	6	4	1,8E-03	8,3E-05	Ptgs1/Alox5/Cyp2e1/Ltc4s/Alox12e/Alox15
GO:0019370	leukotriene biosynthetic process	Yes	Metabolism	3	2	2,5E-03	1,1E-04	Fcer1a/Alox5/Ltc4s
GO:0006704	glucocorticoid biosynthetic process	Yes	Metabolism	3	2	3,0E-03	1,5E-04	Hsd11b1/Cyp11a1/Cyp11b1
GO:0072330	monocarboxylic acid biosynthetic process	Yes	Metabolism	8	5,3	3,5E-03	1,7E-04	Ptgds/Ptgs1/Edn2/Alox5/Aldh1a2/Ltc4s/Alox12e/Alox15
GO:0006633	fatty acid biosynthetic process	Yes	Metabolism	7	4,6	3,5E-03	1,8E-04	Ptgds/Ptgs1/Edn2/Alox5/Ltc4s/Alox12e/Alox15
GO:0019372	lipoxygenase pathway	Yes	Metabolism	3	2	1,5E-03	6,3E-05	Alox5/Alox12e/Alox15
GO:0071621	granulocyte chemotaxis	Yes	Myeloid cell chemotaxis/migration	10	6,6	6,4E-06	9,1E-08	Edn2/Pf4/Ccl24/Ccl22/Ccl17/Lgals3/Ccr3/Ccl8/Ccl9/Ccl6
GO:0030593	neutrophil chemotaxis	Yes	Myeloid cell chemotaxis/migration	9	6	8,5E-06	1,4E-07	Edn2/Pf4/Ccl24/Ccl22/Ccl17/Lgals3/Ccl8/Ccl9/Ccl6
GO:0048245	eosinophil chemotaxis	Yes	Myeloid cell chemotaxis/migration	4	2,6	2,9E-04	9,7E-06	Ccl24/Lgals3/Ccr3/Ccl8
GO:0097530	granulocyte migration	Yes	Myeloid cell chemotaxis/migration	10	6,6	2,9E-05	6,3E-07	Edn2/Pf4/Ccl24/Ccl22/Ccl17/Lgals3/Ccr3/Ccl8/Ccl9/Ccl6
GO:1990266	neutrophil migration	Yes	Myeloid cell chemotaxis/migration	9	6	4,0E-05	1,0E-06	Edn2/Pf4/Ccl24/Ccl22/Ccl17/Lgals3/Ccl8/Ccl9/Ccl6
GO:0072677	eosinophil migration	Yes	Myeloid cell chemotaxis/migration	4	2,6	6,6E-04	2,4E-05	Ccl24/Lgals3/Ccr3/Ccl8
GO:0002548	monocyte chemotaxis	Yes	Myeloid cell chemotaxis/migration	7	4,6	2,3E-05	4,2E-07	Ccl24/Ccl22/Ccl17/Lgals3/Ccl8/Ccl9/Ccl6
GO:0097529	myeloid leukocyte migration	Yes	Myeloid cell chemotaxis/migration	13	8,6	4,9E-06	6,6E-08	Hc/Edn2/Kit/Pf4/Ccl24/Ccl22/Ccl17/Lgals3/Ccr3/Ccl8/Ccl9/Ccl6/Retnlg
GO:0002279	mast cell activation involved in immune response	Yes	Myeloid immunity	13	8,6	1,1E-11	5,4E-15	Fcer1a/Ptgds/Il13ra2/Gata1/Adora3/Kit/Gata2/Mrgprb1/Mrgprx2/Mrgprb2/Enpp3/Il13/Ms4a2
GO:0043303	mast cell degranulation	Yes	Myeloid immunity	12	7,9	5,4E-11	7,8E-14	Fcer1a/Ptgds/Il13ra2/Gata1/Adora3/Kit/Gata2/Mrgprb1/Mrgprx2/Mrgprb2/Il13/Ms4a2
GO:0002448	mast cell mediated immunity	Yes	Myeloid immunity	12	7,9	6,1E-11	1,2E-13	Fcer1a/Ptgds/Il13ra2/Gata1/Adora3/Kit/Gata2/Mrgprb1/Mrgprx2/Mrgprb2/Il13/Ms4a2
GO:0045576	mast cell activation	Yes	Myeloid immunity	13	8,6	1,0E-10	2,9E-13	Fcer1a/Ptgds/Il13ra2/Gata1/Adora3/Kit/Gata2/Mrgprb1/Mrgprx2/Mrgprb2/Enpp3/Il13/Ms4a2
GO:0033006	regulation of mast cell activation involved in immune response	Yes	Myeloid immunity	8	5,3	1,9E-07	1,3E-09	Fcer1a/Il13ra2/Gata1/Adora3/Gata2/Enpp3/Il13/Ms4a2
GO:0043304	regulation of mast cell degranulation	Yes	Myeloid immunity	7	4,6	2,0E-06	1,9E-08	Fcer1a/Il13ra2/Gata1/Adora3/Gata2/Il13/Ms4a2
GO:0033003	regulation of mast cell activation	Yes	Myeloid immunity	8	5,3	2,0E-06	2,1E-08	Fcer1a/Il13ra2/Gata1/Adora3/Gata2/Enpp3/Il13/Ms4a2
GO:0033008	positive regulation of mast cell activation involved in immune response	Yes	Myeloid immunity	6	4	2,1E-06	2,6E-08	Fcer1a/Gata1/Adora3/Gata2/Il13/Ms4a2
GO:0043306	positive regulation of mast cell degranulation	Yes	Myeloid immunity	6	4	2,1E-06	2,6E-08	Fcer1a/Gata1/Adora3/Gata2/Il13/Ms4a2
GO:0033005	positive regulation of mast cell activation	Yes	Myeloid immunity	6	4	1,2E-05	2,2E-07	Fcer1a/Gata1/Adora3/Gata2/Il13/Ms4a2
GO:0033007	negative regulation of mast cell activation involved in immune response	Yes	Myeloid immunity	2	1,3	4,1E-02	3,5E-03	Il13ra2/Enpp3
GO:0002275	myeloid cell activation involved in immune response	Yes	Myeloid immunity	13	8,6	1,5E-09	5,0E-12	Fcer1a/Ptgds/Il13ra2/Gata1/Adora3/Kit/Gata2/Mrgprb1/Mrgprx2/Mrgprb2/Enpp3/Il13/Ms4a2
GO:0002444	myeloid leukocyte mediated immunity	Yes	Myeloid immunity	13	8,6	3,0E-09	1,6E-11	Fcer1a/Ptgds/Il13ra2/Gata1/Adora3/Kit/Gata2/Mrgprb1/Mrgprx2/Mrgprb2/Arg1/Il13/Ms4a2
GO:0002274	myeloid leukocyte activation	Yes	Myeloid immunity	16	10,6	5,2E-08	3,0E-10	Il1rl1/Fcer1a/Ptgds/Il13ra2/Gata1/Adora3/Edn2/Kit/Gata2/Mrgprb1/Mrgprx2/Mrgprb2/Enpp3/Slc7a2/Il13/Ms4a2
GO:0002886	regulation of myeloid leukocyte mediated immunity	Yes	Myeloid immunity	8	5,3	6,2E-06	8,6E-08	Fcer1a/Il13ra2/Gata1/Adora3/Gata2/Arg1/Il13/Ms4a2
GO:0032418	lysosome localization	Yes	Phagocytosis	12	7,9	3,0E-09	1,5E-11	Fcer1a/Ptgds/Il13ra2/Gata1/Adora3/Kit/Gata2/Mrgprb1/Mrgprx2/Mrgprb2/Il13/Ms4a2
GO:1990849	vacuolar localization	Yes	Phagocytosis	12	7,9	3,0E-09	1,5E-11	Fcer1a/Ptgds/Il13ra2/Gata1/Adora3/Kit/Gata2/Mrgprb1/Mrgprx2/Mrgprb2/Il13/Ms4a2
GO:0006911	phagocytosis, engulfment	Yes	Phagocytosis	9	6	2,6E-05	5,3E-07	Gata2/Igkc/Alox15/Ighm/Ighv2-2/Ighv11-2/Ighv1-55/Iglc1/Iglc2
GO:0006910	phagocytosis, recognition	Yes	Phagocytosis	8	5,3	3,1E-05	7,1E-07	Igkc/Ighm/Ighv2-2/Ighv11-2/Ighv1-55/Iglc1/Iglc2/Masp1
GO:0099024	plasma membrane invagination	Yes	Phagocytosis	9	6	4,0E-05	1,0E-06	Gata2/Igkc/Alox15/Ighm/Ighv2-2/Ighv11-2/Ighv1-55/Iglc1/Iglc2
GO:0010324	membrane invagination	Yes	Phagocytosis	9	6	6,0E-05	1,7E-06	Gata2/Igkc/Alox15/Ighm/Ighv2-2/Ighv11-2/Ighv1-55/Iglc1/Iglc2
GO:0006909	phagocytosis	Yes	Phagocytosis	11	7,3	7,4E-04	2,8E-05	Gata2/Igkc/Cebpe/Alox15/Ighm/Ighv2-2/Ighv11-2/Ighv1-55/Iglc1/Iglc2/Masp1
GO:0060326	cell chemotaxis	Yes	Response to cytokine/chemokine	16	10,6	3,7E-07	2,8E-09	Hc/Edn2/Kit/Pf4/Ccl24/Alox5/F7/Ccl22/Ccl17/Lgals3/Ccr3/Ccl8/Ccl9/Ccl6/Enpp2/Retnlg
GO:0070098	chemokine-mediated signaling pathway	Yes	Response to cytokine/chemokine	7	4,6	3,1E-05	6,7E-07	Pf4/Ccl24/Ccl22/Ccl17/Ccl8/Ccl9/Ccl6
GO:1990868	response to chemokine	Yes	Response to cytokine/chemokine	7	4,6	6,5E-05	1,9E-06	Pf4/Ccl24/Ccl22/Ccl17/Ccl8/Ccl9/Ccl6
GO:1990869	cellular response to chemokine	Yes	Response to cytokine/chemokine	7	4,6	6,5E-05	1,9E-06	Pf4/Ccl24/Ccl22/Ccl17/Ccl8/Ccl9/Ccl6
GO:0071346	cellular response to interferon-gamma	Yes	Response to cytokine/chemokine	7	4,6	8,0E-04	3,1E-05	Ccl24/Arg1/Ccl22/Ccl17/Ccl8/Ccl9/Ccl6
GO:0071347	cellular response to interleukin-1	Yes	Response to cytokine/chemokine	6	4	9,7E-04	3,9E-05	Ccl24/Ccl22/Ccl17/Ccl8/Ccl9/Ccl6
GO:0034341	response to interferon-gamma	Yes	Response to cytokine/chemokine	7	4,6	2,5E-03	1,2E-04	Ccl24/Arg1/Ccl22/Ccl17/Ccl8/Ccl9/Ccl6
GO:0070555	response to interleukin-1	Yes	Response to cytokine/chemokine	6	4	2,5E-03	1,2E-04	Ccl24/Ccl22/Ccl17/Ccl8/Ccl9/Ccl6
GO:0019221	cytokine-mediated signaling pathway	Yes	Response to cytokine/chemokine	11	7,3	6,4E-03	3,6E-04	Il13ra2/Edn2/Kit/Pf4/Ccl24/Arg1/Ccl22/Ccl17/Ccl8/Ccl9/Ccl6
GO:0032653	regulation of interleukin-10 production	Yes	Response to cytokine/chemokine	4	2,6	2,3E-02	1,6E-03	Prg2/Il13/Tigit/Pdcd1lg2
GO:0032613	interleukin-10 production	Yes	Response to cytokine/chemokine	4	2,6	2,5E-02	1,8E-03	Prg2/Il13/Tigit/Pdcd1lg2
GO:0001818	negative regulation of cytokine production	Yes	Response to cytokine/chemokine	8	5,3	3,3E-02	2,6E-03	Il1rl1/Gata3/Prg2/Srgn/Arg1/Il13/Tigit/Pdcd1lg2
GO:0002718	regulation of cytokine production involved in immune response	Yes	Response to cytokine/chemokine	5	3,3	4,2E-02	3,6E-03	Fcer1a/Gata3/Prg2/Kit/Arg1
GO:0032689	negative regulation of interferon-gamma production	Yes	Response to cytokine/chemokine	3	2	4,3E-02	3,7E-03	Il1rl1/Gata3/Pdcd1lg2
GO:0032467	positive regulation of cytokinesis	Yes	Response to cytokine/chemokine	3	2	4,7E-02	4,3E-03	Mrgprb1/Mrgprx2/Mrgprb2
GO:0071356	cellular response to tumor necrosis factor	Yes	Response to cytokine/chemokine	6	4	3,1E-02	2,4E-03	Ccl24/Ccl22/Ccl17/Ccl8/Ccl9/Ccl6
GO:0034612	response to tumor necrosis factor	Yes	Response to cytokine/chemokine	6	4	4,5E-02	4,0E-03	Ccl24/Ccl22/Ccl17/Ccl8/Ccl9/Ccl6
GO:0045987	positive regulation of smooth muscle contraction	No	Biological processes	3	2	3,2E-02	2,5E-03	Ptgs1/Edn2/Kit

GO:0031214	biomineral tissue development	No	Biological processes	6	4	3,4E-02	2,7E-03	Wnt6/Gata1/Alox5/Srgn/Alox15/Nfe2
GO:0097067	cellular response to thyroid hormone stimulus	No	Biological processes	2	1,3	3,6E-02	2,9E-03	Gata1/Kit
GO:1903367	positive regulation of fear response	No	Biological processes	2	1,3	3,6E-02	2,9E-03	Penk/Cck
GO:0110148	biomineralization	No	Biological processes	6	4	4,1E-02	3,5E-03	Wnt6/Gata1/Alox5/Srgn/Alox15/Nfe2
GO:0034650	cortisol metabolic process	No	Biological processes	2	1,3	4,1E-02	3,5E-03	Cyp11a1/Cyp11b1
GO:0060215	primitive hemopoiesis	No	Biological processes	2	1,3	4,1E-02	3,5E-03	Gata1/Gata2
GO:0001990	regulation of systemic arterial blood pressure by hormone	No	Biological processes	3	2	4,5E-02	4,0E-03	Cpa3/Edn2/Mcpt4
GO:0030282	bone mineralization	No	Biological processes	5	3,3	4,5E-02	4,0E-03	Gata1/Alox5/Srgn/Alox15/Nfe2
GO:0019229	regulation of vasoconstriction	No	Biological processes	4	2,6	4,9E-02	4,5E-03	Ptgs1/Edn2/Alox5/P2rx1
GO:0007599	hemostasis	No	Biological processes	8	5,3	2,1E-03	9,5E-05	F5/Gata1/Pf4/Tph1/F7/F10/P2rx1/Tpsab1
GO:0035162	embryonic hemopoiesis	No	Biological processes	4	2,6	2,5E-03	1,2E-04	Gata3/Gata1/Kit/Gata2
GO:0001820	serotonin secretion	No	Biological processes	3	2	3,6E-03	1,9E-04	Fcer1a/Lgals3/P2rx1
GO:0042445	hormone metabolic process	No	Biological processes	8	5,3	4,0E-03	2,1E-04	Hsd11b1/Gata3/Duoxa2/Lrat/Cpe/Cyp11a1/Aldh1a2/Cyp11b1
GO:0030279	negative regulation of ossification	No	Biological processes	4	2,6	6,7E-03	3,9E-04	Gata1/Tph1/Srgn/Nfe2
GO:0030502	negative regulation of bone mineralization	No	Biological processes	3	2	8,2E-03	4,8E-04	Gata1/Srgn/Nfe2
GO:0007596	blood coagulation	No	Biological processes	7	4,6	8,8E-03	5,3E-04	F5/Gata1/Pf4/F7/F10/P2rx1/Tpsab1
GO:0050817	coagulation	No	Biological processes	7	4,6	9,7E-03	5,8E-04	F5/Gata1/Pf4/F7/F10/P2rx1/Tpsab1
GO:0070168	negative regulation of biomineral tissue development	No	Biological processes	3	2	1,9E-02	1,2E-03	Gata1/Srgn/Nfe2
GO:0050886	endocrine process	No	Biological processes	5	3,3	1,9E-02	1,2E-03	Gata3/Cpa3/Edn2/Mcpt4/Foxd1
GO:0110150	negative regulation of biomineralization	No	Biological processes	3	2	2,0E-02	1,4E-03	Gata1/Srgn/Nfe2
GO:0070167	regulation of biomineral tissue development	No	Biological processes	5	3,3	2,2E-02	1,5E-03	Wnt6/Gata1/Alox5/Srgn/Nfe2
GO:0110149	regulation of biomineralization	No	Biological processes	5	3,3	2,3E-02	1,6E-03	Wnt6/Gata1/Alox5/Srgn/Nfe2
GO:0050878	regulation of body fluid levels	No	Biological processes	9	6	2,5E-02	1,8E-03	F5/Gata1/Adora3/Pf4/Tph1/F7/F10/P2rx1/Tpsab1
GO:0034754	cellular hormone metabolic process	No	Biological processes	5	3,3	2,6E-02	1,9E-03	Hsd11b1/Lrat/Cyp11a1/Aldh1a2/Cyp11b1
GO:0006940	regulation of smooth muscle contraction	No	Biological processes	4	2,6	3,0E-02	2,3E-03	Ptgs1/Edn2/Kit/P2rx1
GO:0019730	antimicrobial humoral response	No	Microbial	9	6	9,6E-06	1,6E-07	Slpi/Jchain/Pf4/Ccl22/Ccl17/Lgals3/Trf/Ccl8/Ighm
GO:0042742	defense response to bacterium	No	Microbial	13	8,6	5,8E-05	1,6E-06	Slpi/Prg2/Jchain/Igkc/Lyz1/Ear2/Trf/Ighm/Ighv2-2/Ighv11-2/Ighv1-55/Iglc1/Iglc2
GO:0061844	antimicrobial humoral immune response mediated by antimicrobial peptide	No	Microbial	5	3,3	5,8E-03	3,2E-04	Pf4/Ccl22/Ccl17/Lgals3/Ccl8
GO:0019731	antibacterial humoral response	No	Microbial	4	2,6	1,2E-02	7,0E-04	Slpi/Jchain/Trf/Ighm
GO:1903305	regulation of regulated secretory pathway	No	Other	10	6,6	6,0E-05	1,7E-06	Fcer1a/Il13ra2/Gata1/Adora3/Gata2/Il13/P2rx1/Nrn1/Syt12/Ms4a2
GO:0051656	establishment of organelle localization	No	Other	15	9,9	1,2E-04	4,0E-06	Fcer1a/Ptgds/Il13ra2/Gata1/Mgarp/Adora3/Kit/Gata2/Insc/Mrgprb1/Mrgprx2/Mrgprb2/Wasf1/Il13/Ms4a2
GO:1903307	positive regulation of regulated secretory pathway	No	Other	6	4	3,2E-04	1,1E-05	Fcer1a/Gata1/Adora3/Gata2/Il13/Ms4a2
GO:0019835	cytolysis	No	Other	4	2,6	4,5E-04	1,6E-05	Hc/Lyz1/Gzmf/Gzmb
GO:0070374	positive regulation of ERK1 and ERK2 cascade	No	Other	9	6	1,3E-03	5,4E-05	Ccl24/Ccl22/Ccl17/Ccl8/Alox15/Ramp3/Ccl9/Ccl6/Fgfbp3
GO:0070371	ERK1 and ERK2 cascade	No	Other	11	7,3	1,8E-03	8,2E-05	Ccl24/Ccl22/Ccl17/Trf/Ccr3/Ccl8/Alox15/Ramp3/Ccl9/Ccl6/Fgfbp3
GO:0043410	positive regulation of MAPK cascade	No	Other	13	8,6	4,1E-03	2,2E-04	Fcer1a/Kit/Ccl24/Map4k1/Ccl22/Ccl17/Ccl8/Alox15/Ramp3/Ccl9/Ccl6/Ighm/Fgfbp3
GO:0050866	negative regulation of cell activation	No	Other	8	5,3	8,2E-03	4,9E-04	Il13ra2/Pla2g2d/Tarm1/Enpp3/Arg1/Lgals3/Tigit/Pdcd1lg2
GO:0050731	positive regulation of peptidyl-tyrosine phosphorylation	No	Other	7	4,6	1,3E-02	8,1E-04	Fcer1a/Gata1/Kit/Cck/Il13/Ighm/Enpp2
GO:0006837	serotonin transport	No	Other	3	2	1,4E-02	8,6E-04	Fcer1a/Lgals3/P2rx1
GO:0070372	regulation of ERK1 and ERK2 cascade	No	Other	9	6	1,4E-02	9,1E-04	Ccl24/Ccl22/Ccl17/Ccl8/Alox15/Ramp3/Ccl9/Ccl6/Fgfbp3
GO:0015844	monoamine transport	No	Other	5	3,3	1,8E-02	1,2E-03	Fcer1a/Ptgs1/Lgals3/P2rx1/Syt12
GO:0050730	regulation of peptidyl-tyrosine phosphorylation	No	Other	8	5,3	1,9E-02	1,3E-03	Fcer1a/Gata1/Kit/Cadm4/Cck/Il13/Ighm/Enpp2
GO:0015837	amine transport	No	Other	5	3,3	2,7E-02	2,0E-03	Ptgs1/Arg1/Aqp9/Cck/Syt12
GO:0007162	negative regulation of cell adhesion	No	Other	8	5,3	4,5E-02	3,9E-03	Pla2g2d/Tarm1/Arg1/Lgals3/Plet1/Enpp2/Tigit/Pdcd1lg2
GO:0018108	peptidyl-tyrosine phosphorylation	No	Other	8	5,3	4,6E-02	4,1E-03	Fcer1a/Gata1/Kit/Cadm4/Cck/Il13/Ighm/Enpp2
GO:0018212	peptidyl-tyrosine modification	No	Other	8	5,3	4,7E-02	4,3E-03	Fcer1a/Gata1/Kit/Cadm4/Cck/Il13/Ighm/Enpp2
GO:0042060	wound healing	No	Other	12	7,9	1,3E-03	5,7E-05	F5/Gata1/Pf4/Gata2/Alox5/Cadm4/F7/F10/Plet1/Alox15/P2rx1/Tpsab1
GO:0009611	response to wounding	No	Other	13	8,6	4,5E-03	2,4E-04	F5/Hc/Gata1/Pf4/Gata2/Alox5/Cadm4/F7/F10/Plet1/Alox15/P2rx1/Tpsab1

Supplementary table 6. Clinical parameters at baseline for patients in the CheckPAC and TRIPLE-R clinical trials with a high TGFβ-15-specific T cell response at baseline. f: female, m: male, ECOG: Eastern Cooperative Oncology Group, CRP: C-reactive protein. P-values were calculated using Fisher's exact test for categorical variables and Wilcoxon rank sum test or Wilcoxon rank sum exact test as appropriate. No significant differences in any clinical parameter at baseline were detected when comparing both trials.

**Characteristic**	**N**	**CheckPAC**, N = 13[1]	**TRIPLE-R**, N = 10[1]	**p-value**[2]
Sex	23			>0.9
f		5 (38%)	4 (40%)	
m		8 (62%)	6 (60%)	
Age	23	66 (64, 69)	61 (49, 65)	0.2
ECOG Performance score	23			0.4
0		8 (62%)	8 (80%)	
1		5 (38%)	2 (20%)	
CRP (mg/L)	23			0.4
≤ 10		6 (46%)	7 (70%)	
> 10		7 (54%)	3 (30%)	
Leukocytes (10^9 cells/mL)	23	7.00 (6.20, 9.00)	7.40 (6.00, 7.98)	0.8
Lymphocytes (10^9 cells/mL)	23	1.40 (0.80, 1.70)	1.35 (0.73, 1.78)	>0.9
Neutrophil-to-lymphocyte ratio	23	3.4 (2.1, 4.7)	3.5 (2.5, 6.3)	0.8
Albumin (g/L)	23			0.7
≤ 36		3 (23%)	4 (40%)	
> 36		10 (77%)	6 (60%)	
Haemoglobin (mM)	23	7.40 (7.10, 8.20)	7.50 (7.33, 7.93)	0.5
Prior treatments	23			0.3
≤ 2		9 (69%)	9 (90%)	
> 2		4 (31%)	1 (10%)	

[1] n (%); Median (IQR)

[2] Fisher's exact test; Wilcoxon rank sum test; Wilcoxon rank sum exact test
